# Serum metabolite signatures of cardiac function and morphology in individuals from a population-based cohort

**DOI:** 10.1186/s40364-024-00578-w

**Published:** 2024-03-05

**Authors:** Juliane Maushagen, Nuha Shugaa Addin, Christopher Schuppert, Cavin K. Ward-Caviness, Johanna Nattenmüller, Jerzy Adamski, Annette Peters, Fabian Bamberg, Christopher L. Schlett, Rui Wang-Sattler, Susanne Rospleszcz

**Affiliations:** 1 Institute of Epidemiology, Helmholtz Munich, Neuherberg, Germany; 2 Chair of Epidemiology, Institute for Medical Information Processing, Biometry, and Epidemiology (IBE), Medical Faculty, Ludwig- Maximilians-Universität (LMU), München, Germany; 3 Pettenkofer School of Public Health, Munich, Germany; 4 Department of Diagnostic and Interventional Radiology, Faculty of Medicine, Medical Center - University of Freiburg, University of Freiburg, Freiburg, Germany; 5 Center for Public Health and Environmental Assessment, U.S. EPA, Chapel Hill, NC USA; 6 Institute of Experimental Genetics, Helmholtz Zentrum München, German Research Center for Environmental Health, Ingolstädter Landstraße 1, 85764 Neuherberg, Germany; 7 Department of Biochemistry, Yong Loo Lin School of Medicine, National University of Singapore, 8 Medical Drive, 117597 Singapore, Singapore; 8 Institute of Biochemistry, Faculty of Medicine, University of Ljubljana, Vrazov trg 2, 1000 Ljubljana, Slovenia; 9 German Center for Diabetes Research, DZD, Neuherberg, Germany; 10 German Center for Cardiovascular Disease Research (DZHK), Munich Heart Alliance, Munich, Germany; 11 Institute of Translational Genomics, Helmholtz Munich, Neuherberg, Germany

**Keywords:** Metabolites, Imaging, Cardiac function, Cardiac morphology, Left ventricle, Cardiovascular disease, Population-based

## Abstract

**Background:**

Changes in serum metabolites in individuals with altered cardiac function and morphology may exhibit information about cardiovascular disease (CVD) pathway dysregulations and potential CVD risk factors. We aimed to explore associations of cardiac function and morphology, evaluated using magnetic resonance imaging (MRI) with a large panel of serum metabolites.

**Methods:**

Cross-sectional data from CVD-free individuals from the population-based KORA cohort were analyzed. Associations between 3T-MRI-derived left ventricular (LV) function and morphology parameters (e.g., volumes, filling rates, wall thickness) and markers of carotid plaque with metabolite profile clusters and single metabolites as outcomes were assessed by adjusted multinomial logistic regression and linear regression models.

**Results:**

In 360 individuals (mean age 56.3 years; 41.9% female), 146 serum metabolites clustered into three distinct profiles that reflected high-, intermediate- and low-CVD risk. Higher stroke volume (relative risk ratio (RRR): 0.53, 95%-CI [0.37; 0.76], p-value < 0.001) and early diastolic filling rate (RRR: 0.51, 95%-CI [0.37; 0.71], p-value < 0.001) were most strongly protectively associated against the high-risk profile compared to the low-risk profile after adjusting for traditional CVD risk factors. Moreover, imaging markers were associated with 10 metabolites in linear regression. Notably, negative associations of stroke volume and early diastolic filling rate with acylcarnitine C5, and positive association of function parameters with lysophosphatidylcholines, diacylphosphatidylcholines, and acylalkylphosphatidylcholines were observed. Furthermore, there was a negative association of LV wall thickness with alanine, creatinine, and symmetric dimethylarginine. We found no significant associations with carotid plaque.

**Conclusions:**

Serum metabolite signatures are associated with cardiac function and morphology even in individuals without a clinical indication of CVD.

**Supplementary Information:**

The online version contains supplementary material available at 10.1186/s40364-024-00578-w.

## Introduction

Cardiovascular diseases (CVD), such as heart failure (HF) and coronary artery disease, are among the leading causes of morbidity and mortality worldwide [1]. The prevalence of CVD is expected to increase by more than 40% between 2015 and 2035, and the number of deaths is expected to increase 2.8-fold between 2000 and 2050 (in US) [2]. Early detection of individuals at risk and subsequent intervention, e.g., lifestyle modifications, offers an opportunity for the prevention of disease progression and clinical events. Thus, there are ongoing efforts to identify novel biomarkers, based on pathophysiological processes within cardiovascular metabolism, that could help to identify high-risk individuals early.

Biomarkers based on high-throughput metabolomics can help to describe CVD pathways [3] and metabolites measured in serum or plasma have already been linked to both incident and prevalent CVD [4–6]. Metabolites from different chemical groups such as acylcarnitines, amino acids, biogenic amines, dicarboxylacylcarnitines, and lipids have been found to be associated with incident CVD [4]. Combinations of metabolites including the amino acid alanine can predict incidence of major cardiovascular events [7] and different metabolite panels can distinguish between HF subtypes [8]. Moreover, metabolites as intermediate products link genotypes to CVD phenotypes. For example, a genetic risk score of pyroglutamine, dihydroxy docosatrienoic acid, and hydroxy (iso)leucine has been associated with an increased risk for HF [9]. Furthermore, metabolites were identified as mediators for cardiometabolic risk factors in a comprehensive genome-wide association study (GWAS) [10].

Taken together, there is great interest to exploit metabolomics to reveal pathophysiological pathways of CVD. The Absolute*IDQ™* p180 kit (BIOCRATES Life Sciences AG, Innsbruck, Austria) is a high-throughput tool for basic, clinical, and epidemiological research, and its interlaboratory reproducibility has been assessed [11]. The panel has been used for investigations of metabolomics and CVD in cohort and case-control studies. Metabolites of the panel were associated with a higher risk for coronary heart disease [12], improved MI risk prediction [13], or distinguished between HF subtypes [8]. However, to maximize the relevance of metabolomics for prevention or intervention it is necessary to evaluate these metabolites already at the stage of subclinical alterations in cardiac function and morphology before overt CVD has developed. For this type of investigation, population-based studies comprising individuals at all stages of cardiac dysfunction with data on markers of cardiac function and morphology derived by medical imaging are optimal. In the Framingham Heart Study, phosphatidylcholines and diacylglycerols were associated with markers of cardiac morphology as derived by echocardiography [14]. Likewise, in the EpiHealth Study, several metabolites were associated with cardiac markers as measured by echocardiography [15]. However, the gold standard to assess cardiac function and morphology as well as carotid plaques is magnetic resonance imaging (MRI) as it enables a detailed assessment of volumes, tissue, diffuse myocardial fibrosis and plaque composition [16]; hence an in-depth quantification by MRI will provide a more robust determination of cardiac function and a better understanding of underlying pathways.

In the current study, we thus aim to explore the association of cardiac function and morphology, as derived by MRI, with a panel of serum metabolites in a sample from a population-based cohort without known CVD or chronic kidney disease. Our objective is to determine whether there are distinct metabolite signatures of left ventricular (LV) function or morphology or carotid plaque burden at a preclinical stage, and whether these signatures can be mapped to known underlying pathways.

## Methods

### Study sample

The sample is a cross-sectional substudy from the Cooperative Health Research in the Region of Augsburg (KORA) FF4 survey. The FF4 study, N = 2279, is the second follow-up of the population-based KORA S4 study (1999/2001, N = 4261). Of these, 400 individuals in FF4 aged between 39 and 73 years underwent whole body MRI imaging. Participants were included in the MRI subsample if they were not older than 73 years and had no validated/self-reported stroke, myocardial infarction, or revascularization. Individuals were excluded in case of any MRI contraindication or impaired renal function. All MRI participants underwent a comprehensive interview and physical examination within three months before the MRI exam, as described in detail elsewhere [17]. The KORA FF4 study was approved by the Bavarian Medical Association and the MRI substudy by the ethics committee of the Ludwig-Maximilians University Munich. All studies are in accordance with the declaration of Helsinki.

### Main exposure: Left ventricular function and morphology

For cardiovascular imaging, a 3 Tesla scanner (MAGNETOM, Skyra; Siemens AG, Healthcare Sector, Erlangen, Germany) was used in combination with an 18-channel body coil and the table-mounted spine matrix coil. Details on the MRI protocol were described previously [17]. For the examination of LV function and morphology, cine steady-state free precession (cine-SSFP) sequences in the short-axis and the long-axis 4-chamber views were used. The semi-automatically analyses of the cine-SSFP sequences were performed with cvi42 software (Circle Cardiovascular Imaging, Calgary, Canada). The LV wall thickness (mm) at end-diastole was assessed semiautomatically by manually marking the apex and base in the 4-chamber view, followed by an automatic endocardium-epicardium border detection in the short-axis images and manual correction, if needed [17, 18]. According to AHA 16-segment model, the mean LV wall thickness was divided into basal (1-6), mid-cavity (7-12), apical (13-16), lateral (5, 11, 12, 16), septal (2, 3, 8, 9, 14), anterior (1, 7, 13), and inferior segments (4, 10, 15) [19].

An in-house software was used to derive estimates of LV volume changes over time: early diastolic filling rate (pfr1, ml/s) and late diastolic filling rate (pfr2, ml/s), as well as peak systolic ejection rate (per, ml/s) [20]. Further LV parameters included were stroke volume (SV, ml), cardiac output (CO, ml*bpm), ejection fraction (EF, %), end-diastolic and end-systolic LV mass (g) as well as end-diastolic (EDV) and end-systolic volume (ESV, ml). Additionally, the presence of late gadolinium enhancement (LGE) was assessed using the fast low-angle shot inversion recovery sequences in short-axis and 4-chamber views [17].

All image analyses were conducted according to standardized guidelines [21] and by two blinded, and independent readers in cardiac imaging, who were unaware of the participants’ clinical data. Measurements showed intraclass correlation coefficients > 0.9 [18].

### Secondary exposure: carotid plaques

Presence and composition of carotid plaques were assessed on black-blood T1-weighted sequences in 14 slice locations in the internal and common carotid artery. A semi-automatic software (CASCADE; University of Washington Seattle, WA) was used to classify plaques as type I, III, IV/V, VI, and VII, following the guidelines for MRI measurement [22, 23]. Presence of plaque was defined as plaque type above I. Furthermore, the normalized wall index, and the plaque index were calculated. The normalized wall index was calculated by dividing the wall area by the total vessel area. The plaque index was derived from the assessment of plaque composition and divided into the three categories of normal to diffuse thickness (type I), plaques to complex plaques (type III) or fibrotic plaque (type IV/V, VI and VII).

### Additional exposures

As additional exposures, we analyzed hypertension and self-reported angina pectoris. The SCORE2 risk score of 10-year CVD [24] was used as a marker of subclinical CVD. SCORE2 was calculated for all individuals, including those with diabetes.

### Outcome: targeted metabolomics

The KORA study is a deeply phenotyped population-based cohort and comprises a large panel of -omics measurements that were assessed independently of the current MRI study.

Participants were examined between June 2013 and September 2014 at the KORA study center using standardized measurements including questionnaires and laboratory assessment of blood samples [25]. Blood samples were collected at the study center after at least 8 h of fasting and samples were prepared as described elsewhere [26]. Between February 2019 and October 2019 serum metabolites were measured in 2,218 samples from KORA FF4 study using a targeted metabolomics approach [27]. The Absolute*IDQ™* p180 kit (BIOCRATES Life Sciences AG, Innsbruck, Austria) was used for quantification. The samples were distributed randomly across 29 kit plates. For quality assurance, each plate included five identical pooled EDTA-plasma reference samples from Sera Laboratories International Ltd. (Hull, United Kingdom). These reference samples were used to monitor technical variability and ensure consistent quantification across plates. Supplementary Table 1 of additional file 1 shows the number of samples from the current study and reference samples per plate. We implemented a rigorous quality control (QC) protocol, excluding any metabolite that met any of the following criteria: (1) a coefficient of variance (CV) was ≥ 25% across the 145 reference samples, (2) a limit of detection (LOD) ≥ 50% of the metabolite concentrations on any given plate, where the LOD was defined as 3 times the median of 3 PBS zero samples per plate, or (3) a non-detectable rate was ≥ 50% across all plates [28]. Out of 188 targeted metabolites, 146 met the QC criteria and were adjusted for plate-specific normalization factors (NFs). The NF for each metabolite was calculated by dividing the mean concentration of the 5 reference samples per plate by the overall mean concentration of the 145 reference samples [29]. Supplementary Table 2 of additional file 1 shows all analyzed metabolites and their respective abbreviations, whereas Supplementary Table 3 of additional file 1 shows the CVs of the reference samples. Afterwards, the metabolite concentrations were natural log (+ 1) transformed and scaled per plate (mean = 0, SD = 1) to further minimize plate effects. Supplementary Table 4 of additional file 1 shows the mean concentrations of the study sample before and after plate-standardization. Metabolites comprised groups of amino acids, biogenic amines, carnitines, lysophosphatidylcholines (lyso-PC), sphingomyelins (SM), diacylphosphatidylcholines (diacyl-PC), acylalkylphosphatidylcholines (acyl-alkyl-PC) and hexoses (Supplementary Table 2).

### Clinical data

Participants’ age, sex, body mass index (BMI), physical activity (active vs. not active), smoking status (never, ex- and current smoker), alcohol consumption, blood pressures, diabetes status, blood lipid profile, and medication intake were assessed during the visit at the study center by standardized examinations and interviews conducted by trained staff, as previously described [17]. Hypertension was defined as systolic blood pressure ≥ 140 mmHg, diastolic blood pressure ≥ 90 mmHg, or current antihypertensive medication intake under the awareness of having hypertension. Based on oral glucose tolerance test (OGTT) and physician-validated self-reporting, diabetes status was categorized according to WHO criteria. Total cholesterol, high density lipoprotein (HDL), low density lipoprotein (LDL), and triglycerides were measured by enzymatic, colorimetric methods [18]. High sensitivity C-reactive protein (hsCRP) concentrations were measured by particle-enhanced immunonephelometry.

### Statistical methods

### Descriptive analysis

MRI-derived variables, metabolites, and demographic characteristics are given as mean and standard deviation for continuous variables and as absolute and relative frequency for categorical variables. Where appropriate, MRI variables were indexed for body surface area according to Du Bois [30], to take potential confounding effects of body size into account. Differences between groups were quantified by one-way ANOVA and χ^2^-test, as applicable.

### Metabolite profiles

To identify distinct serum metabolite profiles, we used an unsupervised clustering approach on the scaled metabolite panel for the main sample and the secondary sample. For k-means clustering, the optimal number of clusters was based on Calinski-Harabasz Criterion, silhouette plot, and the elbow-method. Details on the methods are described in Additional file 1 (Supplemental Information 1, Supplementary Table 5, and Supplementary Figs. 1–2). Well-separated and compact clusters were then obtained by the Hartigan-Wong algorithm. To test the stability of clusters, we replicated cluster segregation for the main exposure sample with agglomerative hierarchical clustering and calculated Jaccard indices for all clusters.

### Pathway analysis

Differences in metabolic pathways between clusters, reflecting different metabolite profiles, contribute to the understanding of which metabolic pathways may be involved in CVD pathology. Therefore, we performed pathway analysis using MetaboAnalyst 5.0 [31, 32]. Pathway analysis is based on the Kyoto Encyclopedia of Genes and Genomes (KEGG) for homo sapiens database with 80 possible pathways. MetaboAnalyst5.0 combines the three steps of (1) enrichment analysis using Fisher’s exact test, (2) topology analysis using relative-betweenness centrality, and (3) importance measure. For pathway analysis, only those metabolites were considered that were significantly different between the clusters identified in the step “Metabolite Profiles” above. A metabolite was considered to be significantly different between all three clusters if the three Bonferroni-corrected p-values from the three one-way ANOVAs (Cluster 1 vs. Cluster 2, Cluster 1 vs. Cluster 3, Cluster 2 vs. Cluster 3) were all below 0.05. False-discovery corrected p-values ≤ 0.05 are considered to indicate significantly enriched pathways. However, results need to be interpreted with caution because a conservative, nonspecific background-set was used [33].

### Association between cardiac function and morphology and serum metabolites

To identify associations between markers of cardiac function and morphology and metabolite clusters, multinomial logistic regression models were fitted after checking the independence of irrelevant alternatives (IIA) assumption by Hausman-McFadden test. Effect estimates are given as relative risk ratios (RRR) with corresponding 95% confidence intervals (CI). We used a nested model approach for the confounder adjustment strategy with a basic and a full model. The basic model was adjusted for age and sex, and the full model was further adjusted for diabetes, systolic blood pressure, total cholesterol, and smoking status. These established risk factors were chosen as confounders based on prior clinical knowledge [34]. As a sensitivity analysis, we additionally adjusted for hsCRP in addition to the full model. Main exposures were MRI-derived parameters of LV function and morphology, secondary exposures were MRI-derived parameters of carotid plaque, and additional exposures were the non-imaging CVD risk factor hypertension, as well as SCORE2 and presence of angina pectoris as markers of subclinical CVD. All continuous exposures were scaled (mean subtracted and divided by standard deviation) before modeling to interpret results as the effect of an increase by one standard deviation.

To identify associations between markers of cardiac function and morphology and individual metabolites, linear regressions were fitted with the same adjustments as above, and effect estimates are given as beta coefficients and associated 95% CI. P-values were Bonferroni-corrected for the number of metabolites, 146.

All statistical analyses were conducted in R version 4.1.1. P-values ≤ 0.05 were considered statistically significant.

## Results

### Study sample

**Fig. 1:**
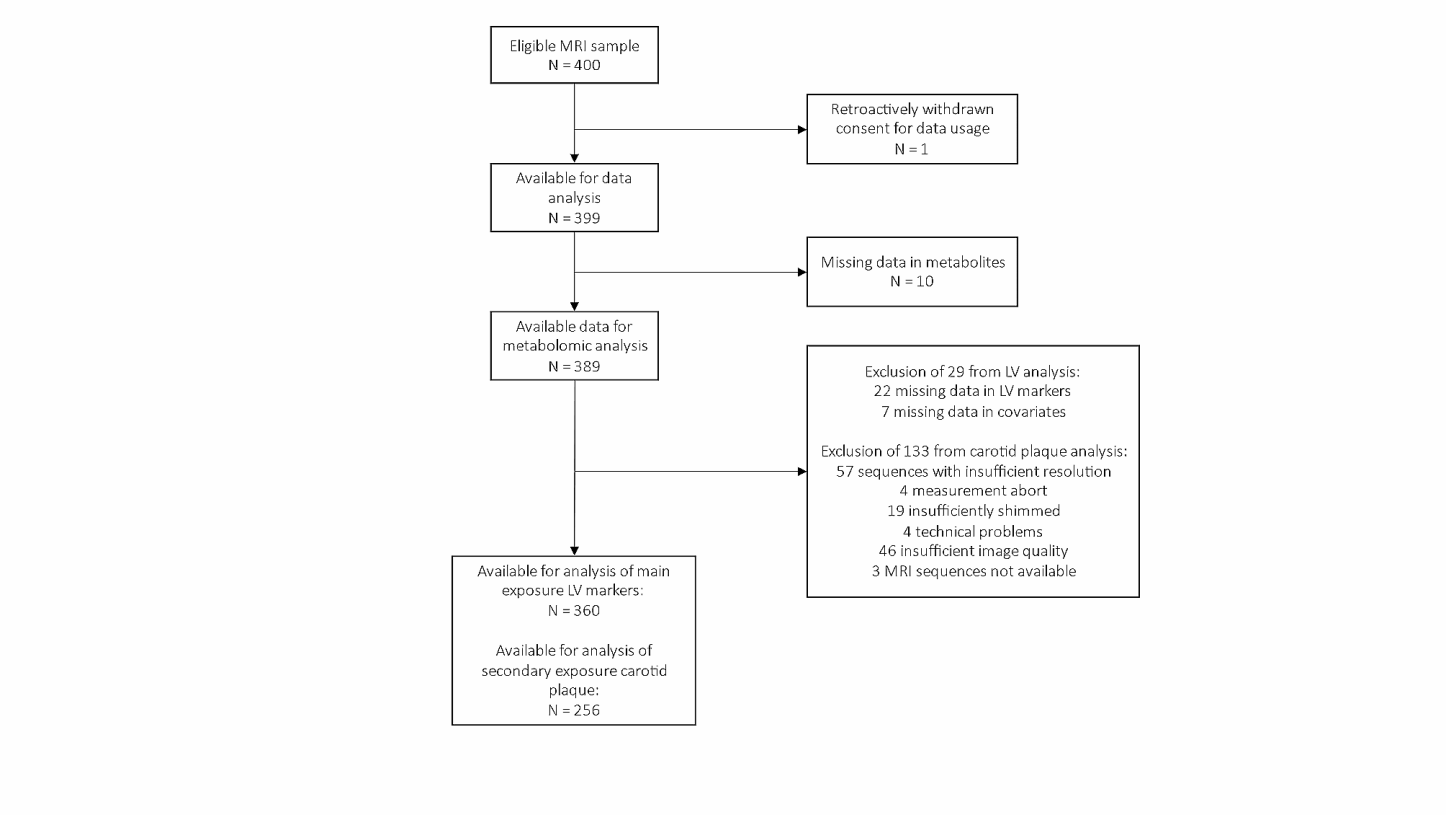
Flowchart of the sample sizes for the main exposure and secondary exposure data sets

The main analytical sample comprised 360 individuals with complete data on LV function and morphology (Fig. 1). The secondary sample contained 256 individuals with complete data on carotid plaques (Fig. 1). This difference in sample size was due to the larger number of individuals without valid plaque measurements. Individuals without complete carotid plaque measures were more likely to be female, had a higher BMI, and were more likely to have prediabetes. Furthermore, several metabolite concentrations differed significantly (Additional file 1: Supplementary Table 6). In the main sample, 151 (41.9%) individuals were female, and the mean age was 56.3 years (Table 1). The average CVD risk within 10 years as estimated by SCORE2 was 5.2%.

**Table 1:** Characteristics of participants of the main exposure sample. Continuous variables are reported as mean (SD) and categorical variables as absolute frequency (percentage)

Variable	Total *N* = 360
Age (years)	56.3 (9.2)
Female sex	151 (41.9%)
BMI (kg/m^2^)	28.0 (4.8)
Regularly physically active	215 (59.7%)
Smoking status	
Currently smoking	72 (20%)
Ex - Smoker	156 (43.3%)
Never - Smoker	132 (36.7%)
Alcohol consumption (g/day)	18.3 (24.0)
Diabetes status (based on OGTT)	
Normoglycemic	221 (61.4%)
Prediabetes	97 (26.9%)
Type 2 diabetes	42 (11.7%)
Fasting glucose (mg/dl)	103.8 (22.6)
Hypertension	115 (32.0%)
Systolic blood pressure (mmHg)	120.3 (16.7)
Diastolic blood pressure (mmHg)	75.4 (10.0)
Angina pectoris	23 (6.4%)
Total cholesterol (mg/dl)	217.68 (36.4)
HDL cholesterol (mg/dl)	61.59 (17.1)
LDL cholesterol (mg/dl)	139.8 (32.9)
Triglycerides (mg/dl)	130.7 (85.2)
hsCRP (mg/L) *	1.15 (0.6; 2.5)
Antidiabetic drugs	24 (6.7%)
Antihypertensive drugs	84 (23.3%)
Lipid lowering drugs	37 (10.3%)
Anticoagulant drugs	6 (1.7%)
Antiplatelet drugs	13 (3.6%)
SCORE2, %	5.2 (3.8)

Continuous variables are reported as mean (SD) and categorical variables as absolute frequency (percentage).

*Based on 359 individuals; reported as median, 1st and 3rd quantile.

*Abbreviations* BMI = Body mass index; OGTT = Oral glucose tolerance test; HDL = High-density lipoprotein; LDL = low-density lipoprotein; hsCRP = High-sensitivity C-reactive protein.

Average values of MRI parameters of LV function and morphology were within the non-pathological range, e.g., mean EF was 69.3% (SD = 7.8) and mean SV and pfr1 were 45.1ml/m^2^ (SD = 9.5) and 227.2 ml/s/m^2^ (SD = 8.6), respectively (Table 2). LGE was present in 20 (5.6%) individuals and average LV wall thickness was 4.8 mm/m^2^ (SD = 0.7) (Table 2).

**Table 2:** MRI-derived parameters of participants in the main sample. Continuous variables are reported as mean (SD) and categorical variables as absolute frequency (proportion)

Variable	Total *N* = 360
End diastolic volume (ml/m^2^)	65.7 (14.9)
End systolic volume (ml/m^2^)	20.6 (8.6)
Stroke volume (ml/m^2^)	45.1(9.5)
Cardiac output (ml/min/m^2^)	2973.1 (590)
Ejection fraction (%)	69.3 (7.8)
Cardiac mass, diastolic (g/m^2^)	70.9 (13.8)
Cardiac mass, systolic (g/m^2^)	72.0 (15.4)
Late Gadolinium Enhancement	20 (5.6%)
Early diastolic filling rate (ml/s)	227.2 (116.4)
Late diastolic filling rate (ml/s)	227.5 (110.4)
Peak ejection rate (ml/s)	357.8 (133.5)
**LV wall thickness per segment**	
All segments (mm/m^2^)	4.8 (0.7)
Basal (mm/m^2^)	5.1 (0.7)
Mid (mm/m^2^)	4.9 (0.8)
Apical (mm/m^2^)	4.9 (0.8)
Lateral (mm/m^2^)	5.0 (0.7)
Septal (mm/m^2^)	4.7 (0.7)
Anterior (mm/m^2^)	4.8 (0.9)
Inferior (mm/m^2^)	4.8 (0.7)

Continuous variables are reported as mean (SD) and categorical variables as absolute frequency (proportion).

*Abbreviations* LV = Left ventricle

### Metabolite profiles

Unsupervised clustering with k-means on the metabolite panel revealed three distinct clusters including 116 (32%, Cluster 1), 106 (29%, Cluster 2), and 138 (38%, Cluster 3) participants, respectively. Clusters showed high stability with Jaccard indices > 0.6, indicating a valid grouping of the data (Additional file 1: Supplementary Table 10). Agglomerative hierarchical clustering could reproduce Cluster 3 well, however Clusters 1 and 2 were only partly replicated (Additional file 1: Supplementary Table 11). Moreover, the lower cluster stability in hierarchical clustering (< 0.6 for Clusters 1 and 2, Additional file 1: Supplementary Table 10) indicated that k-means clustering better captured the underlying data structure. We thus continued with these clusters.

Metabolite concentrations differed significantly between at least two clusters for 145 out of 146 metabolites and differed significantly between all three clusters for 68 out of 146 metabolites (Additional file 1: Supplementary Table 7, Supplementary Fig. 3A-E). For these 68 metabolites, pathway analysis was performed later on.

Cluster 1 was characterized by the highest concentrations of alanine and the majority of further amino acids, and biogenic amines, carnitine, and acylcarnitines including C5, hexoses, and lyso-PCs, however showing intermediate concentrations for lyso-PC 17:0, high to intermediate concentrations of diacyl-PCs, and intermediate concentrations of acyl-alkyl-PCs and hydroxysphingomyelines (Additional file 1: Supplementary Table 7). Individuals in Cluster 2 exhibited intermediate values for the majority of metabolites; however, concentrations of amino acids including alanine, short-chain carnitines (C2-C5) and hexose were lowest while the concentrations of all acyl-alkyl-PCs, lyso-PC 17:0 and hydroxysphingomyelines were highest of all clusters (Additional file 1: Supplementary Table 7). Cluster 3 generally showed the lowest concentrations of metabolites, apart from amines including alanine, short-chain carnitines, and hexoses (Additional file 1: Supplementary Table 7).

**Fig. 2:**
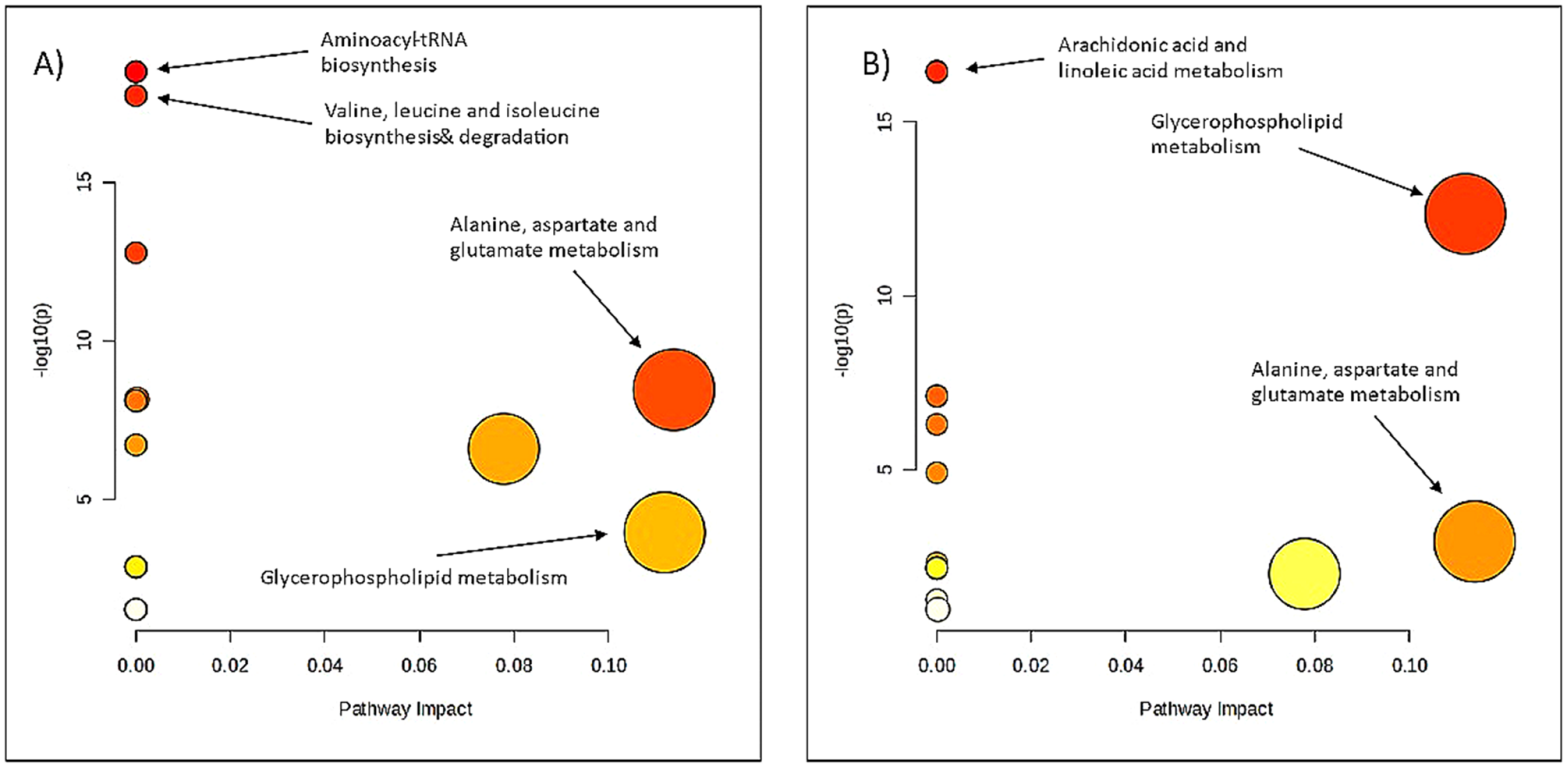
Pathway analysis of 68 metabolites that differed significantly between the three clusters using MetaboAnalyst. **A**: Cluster 1 compared to Cluster 2; **B**: Cluster 3 compared to cCluster 2. The x-axis shows the pathway impact of the topology analysis and the y-axis the -log10 p-value of the enrichment analysis. The color indicates the -log10 p-value where white indicates a low value and red a high value; the greater the circle size the higher the pathway impact.

Pathway analysis of the 68 metabolites that had significantly different concentrations between the three clusters showed that relative to Cluster 2, the glycerophospholipid metabolism, alanine, aspartate, and glutamate metabolism had the highest impacts in both Clusters 1 and 3 with an impact factor > 0.1 (Fig. 2, Additional file 1: Supplementary Table 8). Additionally, aminoacyl-tRNA biosynthesis, valine, leucine, and isoleucine biosynthesis and degradation were the most enriched pathways in Cluster 1 and arachidonic acid and linoleic acid metabolism in Cluster 3. However, these pathways did not show a significant topology impact and the compounds found were only one per pathway (Additional file 1: Supplementary Table 8).

In the secondary exposure sample, three clusters of the sizes 80 (31%), 98 (39%), and 76 (30%) individuals respectively were found (Additional file 1: Supplementary Table 9). Concentrations differed significantly in 138 metabolites between at least two clusters and in 59 metabolites between all three clusters. Cluster 1 showed most amines with lowest concentrations, as well as carnitine and acylcarnitines. Lyso-PCs, diacyl-PCs were mostly intermediate whereas acyl-alkyl-PCs were mostly highest. Cluster 2 was characterized by mostly lowest concentrations of all metabolites and Cluster 3 mostly highest or intermediate concentrations.

### Clinical characteristics of metabolite profiles

In the main data set, Cluster 1 had the highest average age (58.8 years), highest proportion of men, highest prevalence of hypertension, as well as highest levels of LDL cholesterol, triglycerides, alcohol consumption, and CVD risk as measured by SCORE 2 (Table 3). Cluster 2 was mainly characterized by the high proportion of women (73.6%) and the most favorable cardiometabolic risk profile, as seen by a low prevalence of hypertension and diabetes, high HDL levels, and a high prevalence of physical activity (Table 3). Cluster 3 had the lowest average age (54.3 years) but the highest prevalence of diabetes, lipid-lowering medication, and former smoking; moreover, physical activity was lowest in cluster 3 and CVD risk as measured by SCORE 2 intermediate of all clusters (Table 3). Based on these characteristics, Cluster 1 can be labeled as the high CVD risk cluster and Cluster 3 as the intermediate CVD risk cluster in our sample.

**Table 3:** Demographic characteristics and MRI variables of the clusters based on metabolites

Characteristic	Cluster 1 *N* = 116	Cluster 2 *N* = 106	Cluster 3 *N* = 138	p value
Sociodemographic variable				
Age (years)	58.8 (8.7)	56.2 (9.1)	54.3 (9.2)	< 0.001
Female sex	25 (21.6%)	78 (73.6%)	48 (34.8%)	< 0.001
BMI (kg/m2)	28.8 (4.3)	25.8 (4.5)	28.9 (4.9)	< 0.001
Regularly physically active	68 (58.6%)	79 (74.5%)	68 (49.3%)	< 0.001
Alcohol consumption (g/day)	26.7 (30.9)	10.6 (12.9)	17.1 (21.7)	< 0.001
Smoking status				0.415
Currently smoking	24 (20.7%)	23 (21.7%)	25 (18.1%)	
Ex- smoker	49 (42.2%)	39 (36.8%)	68 (49.3%)	
Never-smoker	43 (37.1%)	44 (41.5%)	45 (32.6%)	
Diabetes status				< 0.001
Normoglycemic	60 (51.7%)	82 (77.4%)	79 (57.2%)	
Prediabetes	41 (35.3%)	20 (18.9%)	36 (26.1%)	
Diabetes	15 (12.9%)	*n* < 5	23 (16.7%)	
Fasting glucose (mg/dl)	105.8 (22.8)	96.5 (11.92)	107.8 (27.15)	< 0.001
Hypertension	46 (39.7%)	19 (17.9%)	50 (36.2%)	< 0.001
Systolic blood pressure (mmHg)	125.5 (15.6)	113.0 (14.8)	121.6 (17.1)	< 0.001
Diastolic blood pressure (mmHg)	77.14 (8.8)	71.7 (8.7)	76.70 (11.1)	< 0.001
Angina pectoris	7 (6.0%)	11 (10.4%)	5 (3.6%)	0.100
Total cholesterol (mg/dl)	229.8 (34.8)	230.1 (35.6)	198.0 (29.2)	< 0.001
HDL cholesterol (mg/dl)	59.1 (13.9)	73.7 (17.5)	54.4 (14.1)	< 0.001
LDL cholesterol (mg/dl)	150.24 (32.1)	145.01 (34.42)	127.0 (28.1)	< 0.001
Triglycerides (mg/dl)	159.0 (109.2)	97.6 (51.5)	132.2 (73.8)	< 0.001
hsCRP*	1.29 (0.73; 2.9)	0.91 (0.42; 2.1)	1.35 (0.72; 2.55)	0.350
Antidiabetic drugs	6 (5.2%)	*n* < 5	15 (10.9%)	0.033
Antihypertensive drugs	33 (28.4%)	17 (16.0%)	34 (24.6%)	0.083
Lipid lowering drugs	9 (7.8%)	6 (5.7%)	22 (15.9%)	0.018
Anticoagulant drugs	5 (4.3%)	*n* < 5	*n* < 5	0.024
Antiplatelet drugs	8 (6.9%)	*n* < 5	*n* < 5	0.051
Heart rate (bpm) ^†^	69.8 (12.6)	63.5 (10.0)	67.2 (10.0)	< 0.001
SCORE2, %	5.9 (0.5–22.3)	3.2 (0.2–15.7)	4.00 (0.4–14.0)	< 0.001
MRI characteristics				
End diastolic volume in LV (ml/m2)	62.4 (14.2)	68.6 (15.00)	66.2 (15.1)	0.007
End systolic volume in LV (ml/m2)	19.6 (8.2)	20.6 (7.5)	21.5 (9.6)	0.194
Stroke volume (ml/m2)	42.8 (9.2)	48.12 (10.4)	44.7 (8.5)	< 0.001
Cardiac output of LV (ml/min/m2)	2920.7 (625.9)	3018.3 (640.8)	2982.4 (514.6)	0.457
Ejection fraction (%)	69.3 (8.4)	70.5 (7.1)	68.4 (7.8)	0.113
Peak ejection rate (ml/s)	347.0 (140.9)	368.1 (132.4)	358.9 (128.1)	0.497
Cardiac mass, diastolic (g/m2)	73.9 (14.0)	66.4 (12.9)	71.9 (13.4)	< 0.001
Cardiac mass, systolic (g/m2)	75.8 (15.4)	65.7 (14.3)	73.6 (14.8)	< 0.001
Late Gadolinium Enhancement	8 (6.8%)	*n* < 5	8 (5.8%)	0.981
Early diastolic filling rate (ml/s)	193.6 (102.5)	264.0 (128.8)	227.2 (109.4)	< 0.001
Late diastolic filling rate (ml/s))	234.5 (101.0)	230.2 (115.8)	219.6 (113.9)	0.540
LV wall thickness				
All segments (mm/m2)	4.9 (0.7)	4.7 (0.7)	4.7 (0.6)	0.023
Basal (mm/m2)	5.2 (0.8)	5.0 (0.7)	5.0 (0.7)	0.029
Mid (mm/m2)	5.1 (0.9)	4.7 (0.8)	4.9 (0.7)	0.003
Apical (mm/m2)	4.3 (0.8)	4.3 (0.8)	4.2 (0.7)	0.370
Lateral (mm/m2)	5.1 (0.8)	5.0 (0.7)	4.9 (0.6)	0.119
Septal (mm/m2)	5.0 (0.8)	4.6 (0.7)	4.8 (0.7)	< 0.001
Anterior (mm/m2)	4.9 (0.9)	4.7 (0.9)	4.7 (0.8)	0.047
Inferior (mm/m2)	4.9 (0.7)	4.71 (0.7)	4.8 (0.7)	0.128

^*^Reported as median, 1st and 3rd quantile; ^†^Based on 333 participants; Abbreviations: BMI = Body mass index, HDL = High-density lipoprotein, LDL = low density lipoprotein, hsCRP = High-sensitivity C-reactive protein, LV = Left ventricle

Values of cardiac function and morphology markers differed significantly between clusters (Table 3). Cluster 1 had lowest average EDV, ESV, SV, and EF, highest cardiac mass, and wall thickness, as well as lowest peak ejection rate and lowest pfr1. Interestingly, the late filling rate through atrial contraction was higher than the early filling rate (234.5 ml/s vs. 193.6 ml/s) and highest of all clusters. Cluster 2 had highest average SV and EF, lowest cardiac mass, and wall thickness, as well as highest peak ejection rate and pfr1. Values in Cluster 3 were mainly intermediate between those in Cluster 1 and 2 (Table 3).

The clinical characteristics within the secondary data set were not as clearly differentiated as in the main data set (Additional file 1: Supplementary Table 9). Although there was a differential distribution of cardiometabolic risk factors and CVD risk (e.g., SCORE2 = 4.1% in Cluster 1 vs. 5.0% in Cluster 2 vs. 6.6% in Cluster 3), resulting in nominal differences in plaque prevalence (e.g., prevalence of any plaque = 21.2% in Cluster 1 vs. 18.4% in Cluster 2 vs. 25% in Cluster 3), prevalence and characteristics of carotid plaque were not significantly different between clusters (Additional file 1: Supplementary Table 9).

### Association between cardiac function and morphology and metabolites

In all multinomial logistic regression models in the main exposure sample, Cluster 2 was used as reference group. The base model, adjusted for age and sex, showed 9 associations of 5 cardiac function and morphology markers, as well as SCORE2 and hypertension to be significantly associated with metabolite Cluster 1 and 3 membership. The fully adjusted model showed 9 associations of 5 markers, including two non-MRI-derived exposures (Table 4). Increased LV function (e.g., higher EDV, SV, CO, pfr1) exhibited a protective effect, resulting in a decreased risk of membership to the high- and intermediate- CVD risk cluster. SV showed the strongest protective effects. An increase of one SD decreased the relative risk (RR) of cluster membership to the high- and intermediate-risk cluster by 47.2% and 49.3%, respectively. Furthermore, pfr1 showed strong protective effects. An increase by one SD decreased the RR of belonging to the high- and intermediate-risk cluster by 48.8% and 41.1%, respectively. The detailed RRRs, 95% CI, and p-values are provided in Table 4. Moreover, one SD elevated SCORE2 increased the RR of belonging to the high-risk cluster by 2.2-fold and to the intermediate-risk cluster by 3.5-fold.

**Table 4:** Multinomial logistic regression of subclinical CVD markers and cluster membership

	Cluster	RR ratio	95% CI	P value
MRI marker				
End-diastolic volume in LV (ml)	1	**0.557**	**0.391; 0.793**	**0.001**
	3	**0.633**	**0.453; 0.886**	**0.008**
End-systolic volume in LV (ml)	1	0.729	0.516; 1.029	0.072
	3	0.921	0.678; 1.253	0.602
Stroke volume (ml)	1	**0.528**	**0.368; 0.757**	**< 0.001**
	3	**0.507**	**0.354; 0.727**	**< 0.001**
Cardiac output of LV (ml/min)	1	**0.742**	**0.563; 0.979**	**0.035**
	3	**0.643**	**0.490; 0.842**	**0.001**
Ejection fraction of LV (%)	1	1.009	0.725; 1.406	0.956
	3	0.806	0.579; 1.122	0.201
Peak ejection rate (ml/s)	1	**0.737**	**0.547; 0.995**	**0.046**
	3	0.740	0.546; 1.003	0.052
Cardiac mass, diastolic (g)	1	0.911	0.633; 1.312	0.617
	3	0.884	0.608; 1.287	0.521
Cardiac mass, systolic (g)	1	0.976	0.656; 1.452	0.906
	3	1.013	0.673; 1.526	0.950
Late Gadolinium Enhancement	1	0.720	0.165; 3.139	0.662
	3	1.388	0.312; 6.17	0.667
Early diastolic filling rate (ml/s)	1	**0.512**	**0.370; 0.708**	**< 0.001**
	3	**0.589**	**0.434; 0.799**	**< 0.001**
Late diastolic filling rate (ml/s)	1	0.998	0.73; 1.365	0.992
	3	0.923	0.672; 1.268	0.621
All segments (mm/m^2^)	1	1.015	0.720; 1.432	0.931
	3	1.033	0.724; 1.474	0.858
Basal segment (mm/m^2^)	1	1.036	0.746; 1.439	0.833
	3	1.002	0.71; 1.412	0.993
Mid segment (mm/m^2^)	1	1.141	0.804; 1.618	0.460
	3	1.161	0.807; 1.671	0.422
Apical segment (mm/m^2^)	1	0.834	0.604; 1.151	0.270
	3	0.907	0.657; 1.252	0.552
Lateral segment (mm/m^2^)	1	0.929	0.672; 1.284	0.6560
	3	0.856	0.614; 1.194	0.360
Septal segment (mm/m^2^)	1	1.143	0.801; 1.63	0.461
	3	1.158	0.801; 1.676	0.435
Anterior segment (mm/m^2^)	1	0.982	0.696; 1.386	0.918
	3	1.047	0.734; 1.491	0.801
Inferior segment (mm/m2)	1	1.040	0.744; 1.456	0.817
	3	1.175	0.837; 1.65	0.350
**Other markers**				
SCORE2	1	**2.202**	**1.146;4.234**	**0.018**
	3	**3.529**	**1.67; 7.455**	**0.001**
Hypertension	1	**2.152**	**1.02; 4.538**	**0.044**
	3	**2.605**	**1.214; 5.593**	**0.014**
Angina Pectoris	1	0.847	0.27; 2.657	0.776
	3	0.298	0.083; 1.066	0.063

All models were adjusted for age, sex, diabetes, systolic blood pressure, total cholesterol, and smoking status. CI = 95% confidence interval; RR = relative risk; LV = Left ventricle. Continuous exposures are scaled and relative risk ratios are given per standard deviation

Additional adjustment for hsCRP did not substantially affect the results (Additional file 1: Supplementary Table 12).

Multinomial logistic regression for the secondary exposures of carotid plaque did not show any significant results (Additional file 1: Supplementary Table 13).

**Fig. 3:**
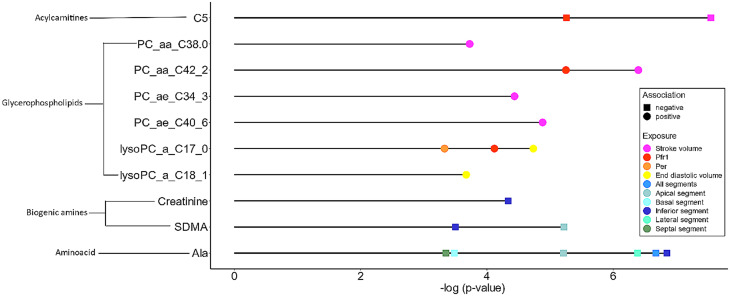
Significant associations from multiple linear regression of LV function and morphology markers and metabolites Results are shown for the fully adjusted model and significance was defined as a Bonferroni corrected p-value ≤ 0.05. The x axis shows the negative log p-value and the y axis the metabolites. Ala = alanine, SDMA = symmetric dimethylarginine, Per = peak ejection rate, pfr1 = early diastolic filling rate

Analyzing single metabolites as outcomes in linear regression, there were 49 significant associations in the base model and 23 significant associations in the full model after Bonferroni correction; most of them were glycerophospholipids (Fig. 3, Additional file 1: Supplementary Table 14). Additional adjustment for hsCRP did not substantially affect the results (Additional file 1: Supplementary Information 2, Supplementary Table 15).

Increased SV was associated with higher diacyl-PCs (PC_aa_C38:0, PCaa_C42:2) and acyl-alkyl-PC (PC_ae_C34:3, Pcae_C40:6). Higher pfr1 was associated with increased levels of diacyl-PC PC_aa_C42:0 and lyso-PC lyso-PC_a_C17:0. Both SV and pfr1 were associated with lower levels of acylcarnitine C5. Moreover, higher EDV was associated with increased lyso-PC (lyso-PC_a_C17:0, lyso-PC_a_C18:1), and also peak ejection rate was associated with lyso-PC_a_C17:0. Increased LV wall thickness in five different segments was associated with lower levels of alanine, symmetric dimethylarginine (SDMA), and creatinine (Fig. 3, Additional file 1: Supplementary Table 14).

Additionally, of the non-MRI-derived exposures, hypertension was associated with the lyso-PC lyso-PC_a_C17:0 and acyl-alkyl-PC PC_ae_C42:2. Furthermore, increased SCORE2 was associated with the acyl-alkyl-PC PC_ae_C38:0 (Additional file 1: Supplementary Table 14).

Presence and characteristics of plaque showed no significant associations with single metabolite concentrations after Bonferroni correction.

## Discussion

CVD biomarkers based on serum metabolomics have great potential to elucidate underlying pathways and may assist in CVD prediction and risk stratification. However, studies assessing metabolite signatures of early changes in cardiac function and morphology are scarce. Thus, we analyzed the association of MRI-derived markers of cardiac function and morphology as well as carotid plaques with targeted serum metabolites in a sample from a population-based cohort without prior CVD or renal impairment. We found that circulating metabolites clustered into three distinct profiles that reflected high-, intermediate- and low-CVD risk as measured by SCORE2. Less favorable markers of LV function (low EDV, SV, CO, pfr1) were associated with higher risk clusters. Moreover, markers of LV function were associated with several glycerophospholipids and the short-chain acylcarnitine C5, whereas markers of LV morphology were associated with amines (alanine, creatine, and SDMA). The associations between non-MRI-derived markers, such as the SCORE2 risk score, and metabolites, similar to the associations seen with MRI-derived markers, suggest that metabolites may play a role in cardiovascular disease pathways reflected in changes of cardiac function and morphology.

### Association of MRI markers with metabolite profiles (cluster membership)

Increased concentrations of several metabolites have been found to be associated with an increased risk for CVD; for instance, based on principal component analysis, a factor containing branched-chain amino acids was associated with increased risk of coronary artery disease, and a factor containing acylcarnitines was associated with increased risk for HF [35]. This is consistent with the gradual increase of most metabolite concentrations according to SCORE2 CVD risk reflected in our clusters. For example, branched-chain amino acids and acylcarnitines were highest in the high-risk cluster.

The cardiac function markers in multinomial logistic regression are related. Cardiovascular impairments that would alter these functional markers are involved in the pathophysiology of heart failure as hemodynamic measures of the pump function. A lower SV, as a measure of subclinical CVD, is associated with higher risk for incident heart failure, independently of other subclinical CVD markers [36]. Additionally, a decreased early diastolic filling rate is associated with HF and shows a negative prognosis for HF [37]. These associations are consistent with our findings that individuals with better LV function are less likely to show unfavorable metabolite profiles.

### Acylcarnitines

The heart requires large amounts of adenosine triphosphate (ATP) for myocyte contraction and relaxation [38]. Oxidative glucose metabolism using glucose, lactate, and ketone bodies as substrates, fatty acid (FA) oxidation using acylcarnitines, or branched chain amino acid metabolism are metabolized for energy synthesis. Substrates are transferred to cell mitochondria and transformed into acyl coenzyme A to be further metabolized in the tricarboxylic acid (TCA) cycle. The use of substrate for energy production depends on the physiological needs. In HF, ATP synthesis is shifted to glycolysis (more ATP per molecule under low oxygen conditions) resulting in decreased mitochondrial uptake of fatty acids [38]. Impaired cardiac function before heart failure shows a lower FA uptake as well, indicating ineffective β-oxidation [39], contributing to pathology of CVDs [40] and leading to decreased contractile LV function [5, 41]. Acylcarnitines serve as carriers for FA transportation into the mitochondria. Hence, in HF, serum acylcarnitine concentrations increase as β-oxidation is reduced [38, 39]. These mechanisms support the association of acylcarnitine C5, as ester of FAs, with cardiac function markers as surrogate for contractile LV function. Long-chain acylcarnitines have been suggested as diagnostic parameter as plasma concentrations reflect those in heart tissue [42]. Long-chain acylcarnitine (C18:2) distinguishes between HF with preserved or reduced EF [8]. Among other acylcarnitines, C5 predicts major cardiac events in elderly people [7]. Decreased short-chain acylcarnitines are associated with improvement in systolic function in patients with acute HF after recovery from the acute condition [43]. This association further confirms the direction of the association of C5 found in the current study. The association of acylcarnitines with subclinical CVD is supported by other studies as well, for example, higher values of medium and long-chain acylcarnitines (C7, C9, C16) are associated with LV diastolic dysfunction (septal or lateral velocity) in women with or at risk for HIV infection [44]. Interestingly, other studies have predominantly found associations of long-chain acylcarnitines (C16, C18, C18:1) with heart failure [45], long-chain acylcarnitines (C16, C18:1, C18:2, C18, and C26) with LV remodeling index [46] and long-chain acylcarnitines (C2:0, C8:0-OH, C12:0, C12:1, C14:0, C14:1, C16:0, C16:1, C18:1) with higher risk for atrial fibrillation [47]. We hypothesize that this association was not visible in our study due to the specific characteristic of our sample, where all participants were free of CVD and presented with cardiac morphology and function in the non-pathological range. Therefore, we might hypothesize that the stage of subclinical CVD is not reflected by metabolite signatures including long-chain acylcarnitines, but that these are characteristic of a later, more advanced stage of CVD.

### Glycerophospholipids

Consistent with the impact of glycerophospholipid metabolism, as found in pathway analysis, 6 different glycerophospholipids showed 9 positive associations with cardiac function parameters in our study. Consistent with the positive association of favorable cardiac function markers, inverse associations of lyso-PCs and PCs with HF were found [8, 14] or inverse associations of other glycerophospholipids with subclinical CVD [15]. In another study from the population-based KORA cohort, circulating lyso-PC_17:0 was associated with a reduced risk for myocardial infarction (MI) [13]. In contrast, diacyl-PC_38:0 has been reported to be associated with CVD mortality [48], whereas in our study it was positively associated with favorable cardiac function markers. The same study found a protective association of acyl-alkyl-PC_40:6 and lyso-PCs with mortality [48]. A recent review on lipidomics illustrated that unsaturated or monosaturated PCs are associated with higher risk of CVD, while polyunsaturated PCs show inverse associations [49]. In our study, 3 out of 4 PCs that were positively associated with function markers contained polyunsaturated fatty acids (Fig. 2). Although studies confirm inverse associations of lyso-PCs and PCs with HF or coronary artery disease, there is controversy about the biological mechanisms for these protective effects that are not yet understood [50]. One suggested pathway by Ward-Caviness et al. is that lower lyso-PC concentrations lead to increased inflammation and oxidative stress. Due to lyso-PCs influencing the synthesis of antioxidative enzymes, lower concentrations of lyso-PC result in higher oxidative stress and enhance inflammation [13]. This mechanism might explain involvement of lyso-PCs to endothelial function. Another pathway suggests that lower 1,2-diacylglycerol (DAG) concentrations, derivatized with different fatty acids including phosphatidylcholines, are associated with cardiomyopathy in an animal model [51]. Lower 1,2-DAG might lead to a decreased activation of phosphokinase C, resulting in lower calcium channel activity and therefore in reduced contractility. This pathway would support our findings since higher concentrations of glycerophospholipids were associated with an improved contractile function as represented as stroke volume. Hydrolysis of phosphatidylcholine splits into lyso-PC and one fatty acid by phospholipase A_2_ (PLA_2_), which is also found in cardiac tissue. In an in vitro study of cardiac myoblast H9c2 cells, lyso-PC was found to increase arachidonate and Ca^2+^ concentrations leading to an activation of phosphokinase C. This, in turn, activated intracellular PLA_2_ [52]. Another study [53] tackled the paradoxical findings for lyso-PCs, suggesting a feedback loop of lyso-PCs inhibiting plasma secretory phospholipase A_2_ (sPLA_2_) as its own product in the context of sepsis. It is suggested that higher lyso-PCs inhibit sPLA_2_ and therefore prevent further pro-inflammatory processes induced by sPLA_2_. Associations of lysoPCs and sphingomyelins with incident coronary heart disease have been hypothesized to be partly mediated through traditional cardiovascular risk factors and markers of inflammation and oxidative stress, but there was no evidence for causality [54]. In conclusion, although the results from our study support the positive association between favorable cardiac function and circulating glycerophospholipids, more studies are needed to further evaluate their suitability as biomarkers of alterations in cardiac function before the onset of CVD.

### Amino acids and hypertrophy / heart failure

The amino acid alanine was associated with five different LV wall segments and the mean LV wall thickness in our analysis. Increased alanine concentrations predict major adverse cardiac events in high-risk individuals free of HF [7]. In contrast, lower concentrations of alanine have been found to predict major adverse events in HF patients [55]. The latter is partly consistent with our finding of lower alanine concentrations to be associated with increased LV wall thickness, indicating precursors of left ventricular hypertrophy (LVH). Pathological LVH is the consequence of chronic pressure overload as myocardial growth stimuli and neurohormones are activated leading to heart failure or cardiac events [56]. A study assessing the transition from LVH to HF in rats found decreased alanine in rats with HF and an impact of the pentose phosphate pathway [57]. Alanine is furthermore suggested as cardioprotective as part of carnosine in combination with histidine [58] acting as antioxidant [59]. Although alanine has been found to be prognostic for cardiac events [7], it is still unclear if it is also predictive of early alterations in morphology and function, and the causality of the association still needs to be investigated.

Our results extend the current evidence on the role of metabolomics in CVD by illustrating that serum metabolite signatures reflect not only overt CVD, but already early alterations of cardiac function and morphology. Interestingly, we have not found any associations with carotid plaque burden. We hypothesize that the plaque burden was not high enough at this sample size to observe alterations in serum metabolites. Metabolite profiles within different plaque types, e.g. stable and vulnerable plaques, have been shown to exhibit different characteristics [60], but is still unclear how this can be reflected by circulating metabolites in serum. Another limitation of the current study is that there were no measures of coronary artery calcification. Thus, future studies with larger sample sizes are needed to investigate the relation between plaques in preclinical condition and metabolites. This is especially relevant since a main pathway that causally relates increased amino acids with CVD seems to work via plaque rupture and thrombus formation, mediated via glucose-regulating and neuroendocrine pathways [61].

The small sample size limited us further to conduct sex- or age-stratified analyses or to include interaction terms in the models. It is important to note that our sample, although originating from a population-based cohort, represents a selected subsample that already underwent two follow-up visits, and had no CVD or contraindications to MRI. Generalizability to the general population is therefore somewhat limited. The use of an unspecific background set in the pathway analyses is an additional limitation, which should be taken into account by future studies assessing enriched pathways as the type one error rate can increase. Moreover, although we have used an established targeted metabolite panel, it is by no means comprehensive, and it will be crucial to repeat our analyses on further metabolite panels. For example, our panel did not provide extensive coverage of polar metabolites, which would be interesting to study since they constitute the majority of metabolites in some interesting pathways, e.g. central carbon metabolism.

Although our findings are an important contribution to our understanding of disease pathways and a potential stepstone for initiating statistical models for risk stratification, it is crucial to note that in this study we did not evaluate if metabolites are predictive of cardiac function or morphology. Our current cross-sectional sample is not adequate to set up a meaningful prediction model, since for prediction of subclinical disease parameters we would need larger samples due to (1) the smaller effect sizes in subclinical vs. overt CVD, (2) the need for separate training and validation data. Thus, we do not make any claims about the predictive ability of serum metabolites on cardiac function and morphology, or causal relations between them. To assess causality, longitudinal data as well as appropriate statistical tools like Mendelian Randomization are needed and should be investigated in future studies.

## Conclusion

In conclusion, our findings illustrate that changes in metabolites occur at early subclinical disease stages and provide evidence on underlying pathophysiological mechanisms. However, more prospective studies are needed to assess the ability of serum metabolites for early prediction of individuals at risk.

### Electronic supplementary material

Below is the link to the electronic supplementary material.

Supplementary Material 1

Supplementary Material 2

## Data Availability

The informed consent given by KORA study participants does not cover data posting in public databases. However, data are available upon request by means of a project agreement. Requests should be sent to kora.passt@helmholtz-muenchen.de and are subject to approval by the KORA Board.

## Abbreviations

CVD: Cardiovascular disease
HF: Heart failure
NF: Normalization factor
GWAS: Genome-wide association study
KORA: Cooperative Health Research in the Region of Augsburg
LV: Left ventricle
pfr1: Early diastolic filling rate
pfr2: Late diastolic filling rate
per: Peak ejection rate
SV: Stroke volume
CO: Cardiac output
EF: Ejection fraction
EDV: End diastolic volume
ESV: End systolic volume
LGE: Late gadolinium enhancement
lysoPC: Lysophosphatidylcholines
SM: Sphingomyelins
diacyl-PC: Diacylphosphatidylcholines
acylalkylPC: Acylalkylphosphatidylcholines
OGTT: Oral glucose tolerance test
SDMA: Symmetric dimethylarginine
ATP: Adenosine triphosphate
FA: Fatty acid
DAG: 1,2-diacylglycerol
PLA2: Phospholipase A2
