# Supplementary material for: Serum metabolite signatures of cardiac function and morphology in individuals from a population-based cohort
Source: Biomark Res. 2024 Mar 5;12:31. doi: 10.1186/s40364-024-00578-w (PMC10916302; doi:10.1186/s40364-024-00578-w)
Supplement: Supplementary file 1 — Supplementary Material 1 [file 40364_2024_578_MOESM1_ESM.docx]

**Additional file 1 to**

**Serum metabolite signatures of cardiac function and morphology in individuals from a population-based cohort**

Juliane Maushagen^1,2,3^, Nuha Shugaa Addin^1,2,3^, Christopher Schuppert^4^, Cavin Ward-Caviness^5^, Johanna Nattenmüller^4^, Jerzy Adamski^6,7,8^, Annette Peters^1,2,9,10^, Fabian Bamberg^4^, Christopher L Schlett^4^, Rui Wang-Sattler^9,11^, Susanne Rospleszcz^1,2,4,10^

[**Supplementary Table 1** Number of samples and reference samples per plate for the MRI substudy. 3](#_Toc157451946)

[**Supplementary Table 2** Metabolite abbreviations 3](#_Toc157451947)

[**Supplementary Table 3** Coefficients of Variance (CV) of all metabolites in the *AbsoluteIDQ^TM^* p180 kit (BIOCRATES Life Sciences AG, Innsbruck, Austria) 6](#_Toc157451948)

[**Supplementary Table 4** Concentrations of the log (+1)-transformed non-standardized and plate-standardized concentrations of all metabolites given as mean (standard deviation). 10](#_Toc157451949)

[**Supplementary Table 5** Calinski-Harabasz index for 3-8 clusters in the main exposure (left ventricle parameters) data set. According to the highest index at 54.60 three clusters fit best to the data. 14](#_Toc157451950)

[**Supplementary Table 6** Description of individuals included in or excluded from the secondary exposure (carotid plaque) data set. Mean (SD) was calculated for continuous variables and frequency (proportion) for categorical values. P-value from t-test or χ^2^test, where applicable. 15](#_Toc157451951)

[**Supplementary Table 7** Metabolite concentrations (µmol/L) per cluster of the main exposure (left ventricle parameters) data set. ANOVA was used to test for differences between clusters. A p-value ≤ 0.05 was considered significant and is marked bold. 19](#_Toc157451952)

[**Supplementary Table 8** Pathway analyses in the main exposure data set. 24](#_Toc157451953)

[**Supplementary Table 9** Clinical characteristics, imaging markers, and metabolite concentrations according to metabolite profile clusters in the secondary data set (carotid plaque). Continuous variables are presented as mean (SD) and categorical variables as frequency (proportion). Significance is defined as a p-value ≤ 0.05 and tested using ANOVA or χ2 Test, where applicable. 25](#_Toc157451954)

[**Supplementary Table 10** Jaccard clusters indices for the clusters for the k-means clusters of the main exposure and secondary exposure data set, as well as for the hierarchical clustering of the main exposure data set. 28](#_Toc157451955)

[**Supplementary Table 11** Comparison of k-means clusters with agglomerative hierarchical clusters; Ward algorithm. The intersection of individuals in the same clusters are shown as absolute numbers. The percentages refer to the proportion of individuals from the k-means clustering who were also assigned to a group by the hierarchical clustering (intersection / N of k-means clusters). 29](#_Toc157451956)

[**Supplementary Table 12** Results of the multinomial logistic regression additionally adjusted for hsCRP for the main data set. Cluster 2 was used as reference. A p-value ≤0.05 is considered as significant. RR = Relative risk; CI = Confidence interval 29](#_Toc157451957)

[**Supplementary Table 13** Results of the multinomial logistic regression for the secondary exposure. All models were adjusted for age, sex, diabetes, hypertension, total cholesterol, and smoking status. Reference = Cluster 1. RR = relative risk; CI = confidence interval 30](#_Toc157451958)

[**Supplementary Table 14** Significant results of multivariable linear regression for the main data set. Adjustment for age, sex, diabetes status, systolic blood pressure, and smoking status. Significance was defined as a Bonferroni corrected p-value ≤ 0.05 30](#_Toc157451959)

[**Supplementary Table 15** Results of the full adjusted linear regression with hsCRP as additional covariable. Significance is defined as a p-value ≤ 0.05 31](#_Toc157451960)

[**Supplementary Figure 1** Within cluster sum of squares method. The y axis shows the number of clusters and the y axis the WSS. The optimal number of clusters is where the WSS difference is much anymore. The graph shows potential number of clusters at 3 – 4 cluster. 14](https://hmgu-my.sharepoint.com/personal/julianelisa_maushagen_helmholtz-munich_de/Documents/MRI_metabolomics_project/journal_submission/BMC/major%20revision/maushagen_supplement_30Jan.docx#_Toc157586045)

[**Supplementary Figure 2** Graphical assessment of the silhouette method with the number of clusters on the x axis and the average silhouette coefficient on the y axis. This method indicates 2 clusters fit best to the data. 15](https://hmgu-my.sharepoint.com/personal/julianelisa_maushagen_helmholtz-munich_de/Documents/MRI_metabolomics_project/journal_submission/BMC/major%20revision/maushagen_supplement_30Jan.docx#_Toc157586046)

[**Supplementary Figures 3A -E** show the concentrations of metabolites (µmol/L) per cluster. The x axis shows the clusters and the y-axis shows the concentration in the scale of the metabolite. 3A) Aminoacids, 3B) carnitine and acylcarnitines 3C) acylalkylphosphatidylcholine, 3D) lysophosphatidylcholine and diacylphosphatidylcholine, 3E) sphingomyelin and hexoses 22](https://hmgu-my.sharepoint.com/personal/julianelisa_maushagen_helmholtz-munich_de/Documents/MRI_metabolomics_project/journal_submission/BMC/major%20revision/maushagen_supplement_30Jan.docx#_Toc157586047)

**Supplemental Information 1** Information on determination of the number of clusters……………………...13

**Supplemental Information 2** hsCRP as additional covariable in the linear regression…...………………….31

**Supplementary Table 1** Number of samples and reference samples per plate for the MRI substudy.

| **Plate** | **Number of samples of the MRI substudy** | **Number of reference samples** |
| --- | --- | --- |
| Plate 1 | 10 | 5 |
| Plate 2 | 8 | 5 |
| Plate 3 | 11 | 5 |
| Plate 4 | 7 | 5 |
| Plate 5 | 16 | 5 |
| Plate 6 | 9 | 5 |
| Plate 7 | 5 | 5 |
| Plate 8 | n < 5 | 5 |
| Plate 9 | 11 | 5 |
| Plate 10 | 8 | 5 |
| Plate 11 | 9 | 5 |
| Plate 12 | 8 | 5 |
| Plate 13 | 10 | 5 |
| Plate 14 | 15 | 5 |
| Plate 15 | 17 | 5 |
| Plate 16 | 16 | 5 |
| Plate 17 | 16 | 5 |
| Plate 18 | 24 | 5 |
| Plate 19 | 18 | 5 |
| Plate 20 | 20 | 5 |
| Plate 21 | 19 | 5 |
| Plate 22 | 21 | 5 |
| Plate 23 | 12 | 5 |
| Plate 24 | 14 | 5 |
| Plate 25 | 7 | 5 |
| Plate 26 | 12 | 5 |
| Plate 27 | 11 | 5 |
| Plate 28 | 14 | 5 |
| Plate 29 | 9 | 5 |

**Supplementary Table 2** Metabolite abbreviations

| Metabolite group | Abbreviation | Biochemical name |
| --- | --- | --- |
| Carnitines | C0 | Carnitine |
|  | C2 | Acetylcarnitine |
|  | C3 | Propionylcarnitine |
|  | C3_DC__C4_OH_ | Hydroxypropionylcarnitine |
|  | C4 | Butyrylcarnitine |
|  | C4_1 | Butenylcarnitine |
|  | C5 | Valerylcarnitine |
|  | C7_DC | Pimelylcarnitine |
|  | C8 | Octanoylcarnitine |
|  | C9 | Nonaylcarnitine |
|  | C10 | Decanoylcarnitine |
|  | C10_1 | Decenoylcarnitine |
|  | C12 | Dodecanoylcarnitine |
|  | C12_1 | Dodecenoylcarnitine |
|  | C14 | Tetradecanoylcarnitine |
|  | C14_1 | Tetradecenoylcarnitine |
|  | C14_1_OH | Hydroxytetradecenoylcarnitine |
|  | C14_2 | Tetradecadienylcarnitine |
|  | C14_2_OH | Hydroxytetradecadienylcarnitine |
|  | C16 | Hexadecanylcarnitine |
|  | C16_1_OH | Hydroxyhexadecenoylcarnitine |
|  | C16_2 | Hexadecadienylcarnitine |
|  | C18 | Octadecanoylcarnitine |
|  | C18_1 | Octadecenoylcarnitine |
|  | C18_2 | Octadecadienylcarnitine |
| Amino acids | Ala | Alanine |
|  | Arg | Arginine |
|  | Asn | Asparagine |
|  | Asp | Aspartate |
|  | Cit | Citrulline |
|  | Gln | Glutamine |
|  | Glu | Glutamate |
|  | Gly | Glycine |
|  | His | Histidine |
|  | Ile | Isoleucine |
|  | Leu | Leucine |
|  | Lys | Lysine |
|  | Met | Methionine |
|  | Orn | Ornithine |
|  | Phe | Phenylalanine |
|  | Pro | Proline |
|  | Ser | Serine |
|  | Thr | Threonine |
|  | Trp | Tryptophan |
|  | Tyr | Tryosine |
|  | Val | Valine |
| Biogenic amines | Ac.Orn | Acetylornithine |
|  | ADMA | Asymmetric dimethylarginine |
|  | alpha.AAA | alpha-Aminoadipic acid |
|  | Creatinine | Creatinine |
|  | Kynurenine | Kynurenine |
|  | Met.SO | Methioninesulfoxide |
|  | SDMA | Symmetric dimethylarginine |
|  | Spermidine | Spermidine |
|  | t4.OH.Pro |  |
|  | Taurine | Taurine |
|  | total.DMA |  |
| Lysophosphatidylcholines | lysoPC_a_C16_0 | lysoPhosphatidylcholine acyl C16:0 |
|  | lysoPC_a_C16_1 | lysoPhosphatidylcholine acyl C16:1 |
|  | lysoPC_a_C17_0 | lysoPhosphatidylcholine acyl C17:0 |
|  | lysoPC_a_C18_0 | lysoPhosphatidylcholine acyl C18:0 |
|  | lysoPC_a_C18_1 | lysoPhosphatidylcholine acyl C18:1 |
|  | lysoPC_a_C18_2 | lysoPhosphatidylcholine acyl C18:2 |
|  | lysoPC_a_C20_3 | lysoPhosphatidylcholine acyl C20:3 |
|  | lysoPC_a_C20_4 | lysoPhosphatidylcholine acyl C20:4 |
| Diacylphosphatidylcholines | PC_aa_C28_1 | Phosphatidylcholine diacyl C28:1 |
|  | PC_aa_C30_0 | Phosphatidylcholine diacyl C30:0 |
|  | PC_aa_C32_0 | Phosphatidylcholine diacyl C30:0 |
|  | PC_aa_C32_1 | Phosphatidylcholine diacyl C32:1 |
|  | PC_aa_C32_2 | Phosphatidylcholine diacyl C32:2 |
|  | PC_aa_C32_3 | Phosphatidylcholine diacyl C32:3 |
|  | PC_aa_C34_1 | Phosphatidylcholine diacyl C34:1 |
|  | PC_aa_C34_2 | Phosphatidylcholine diacyl C34:2 |
|  | PC_aa_C34_3 | Phosphatidylcholine diacyl C34:3 |
|  | PC_aa_C34_4 | Phosphatidylcholine diacyl C34:4 |
|  | PC_aa_C36_1 | Phosphatidylcholine diacyl C36:1 |
|  | PC_aa_C36_2 | Phosphatidylcholine diacyl C36:2 |
|  | PC_aa_C36_3 | Phosphatidylcholine diacyl C36:3 |
|  | PC_aa_C36_4 | Phosphatidylcholine diacyl C36:4 |
|  | PC_aa_C36_5 | Phosphatidylcholine diacyl C36:5 |
|  | PC_aa_C36_6 | Phosphatidylcholine diacyl C36:3 |
|  | PC_aa_C38_0 | Phosphatidylcholine diacyl C38:0 |
|  | PC_aa_C38_1 | Phosphatidylcholine diacyl C38:1 |
|  | PC_aa_C38_3 | Phosphatidylcholine diacyl C38:3 |
|  | PC_aa_C38_4 | Phosphatidylcholine diacyl C38:4 |
|  | PC_aa_C38_5 | Phosphatidylcholine diacyl C38:5 |
|  | PC_aa_C38_6 | Phosphatidylcholine diacyl C38:6 |
|  | PC_aa_C40_2 | Phosphatidylcholine diacyl C40:2 |
|  | PC_aa_C40_3 | Phosphatidylcholine diacyl C40:3 |
|  | PC_aa_C40_4 | Phosphatidylcholine diacyl C40:4 |
|  | PC_aa_C40_5 | Phosphatidylcholine diacyl C40:5 |
|  | PC_aa_C40_6 | Phosphatidylcholine diacyl C40:6 |
|  | PC_aa_C42_0 | Phosphatidylcholine diacyl C42:0 |
|  | PC_aa_C42_1 | Phosphatidylcholine diacyl C42:1 |
|  | PC_aa_C42_2 | Phosphatidylcholine diacyl C42:2 |
|  | PC_aa_C42_4 | Phosphatidylcholine diacyl C42:4 |
|  | PC_aa_C42_5 | Phosphatidylcholine diacyl C42:5 |
|  | PC_aa_C42_6 | Phosphatidylcholine diacyl C42:6 |
| Acylalkylphosphatidylcholines | PC_ae_C30_0 | Phosphatidylcholine acyl-alkyl C30:0 |
|  | PC_ae_C32_1 | Phosphatidylcholine acyl-alkyl C32:1 |
|  | PC_ae_C32_2 | Phosphatidylcholine acyl-alkyl C32:2 |
|  | PC_ae_C34_0 | Phosphatidylcholine acyl-alkyl C34:0 |
|  | PC_ae_C34_1 | Phosphatidylcholine acyl-alkyl C34:1 |
|  | PC_ae_C34_2 | Phosphatidylcholine acyl-alkyl C34:2 |
|  | PC_ae_C34_3 | Phosphatidylcholine acyl-alkyl C34:3 |
|  | PC_ae_C36_0 | Phosphatidylcholine acyl-alkyl C36:0 |
|  | PC_ae_C36_1 | Phosphatidylcholine acyl-alkyl C36:1 |
|  | PC_ae_C36_2 | Phosphatidylcholine acyl-alkyl C36:2 |
|  | PC_ae_C36_3 | Phosphatidylcholine acyl-alkyl C36:3 |
|  | PC_ae_C36_4 | Phosphatidylcholine acyl-alkyl C36:4 |
|  | PC_ae_C36_5 | Phosphatidylcholine acyl-alkyl C36:5 |
|  | PC_ae_C38_0 | Phosphatidylcholine acyl-alkyl C38:0 |
|  | PC_ae_C38_1 | Phosphatidylcholine acyl-alkyl C38:1 |
|  | PC_ae_C38_2 | Phosphatidylcholine acyl-alkyl C38:2 |
|  | PC_ae_C38_3 | Phosphatidylcholine acyl-alkyl C38:3 |
|  | PC_ae_C38_4 | Phosphatidylcholine acyl-alkyl C38:4 |
|  | PC_ae_C38_5 | Phosphatidylcholine acyl-alkyl C38:5 |
|  | PC_ae_C38_6 | Phosphatidylcholine acyl-alkyl C38:6 |
|  | PC_ae_C40_1 | Phosphatidylcholine acyl-alkyl C40:1 |
|  | PC_ae_C40_2 | Phosphatidylcholine acyl-alkyl C40:2 |
|  | PC_ae_C40_3 | Phosphatidylcholine acyl-alkyl C40:3 |
|  | PC_ae_C40_4 | Phosphatidylcholine acyl-alkyl C40:4 |
|  | PC_ae_C40_5 | Phosphatidylcholine acyl-alkyl C40:5 |
|  | PC_ae_C40_6 | Phosphatidylcholine acyl-alkyl C40:6 |
|  | PC_ae_C42_1 | Phosphatidylcholine acyl-alkyl C42:1 |
|  | PC_ae_C42_2 | Phosphatidylcholine acyl-alkyl C42:2 |
|  | PC_ae_C42_3 | Phosphatidylcholine acyl-alkyl C42:3 |
|  | PC_ae_C42_4 | Phosphatidylcholine acyl-alkyl C42:4 |
|  | PC_ae_C42_5 | Phosphatidylcholine acyl-alkyl C42:5 |
|  | PC_ae_C44_3 | Phosphatidylcholine acyl-alkyl C44:3 |
|  | PC_ae_C44_4 | Phosphatidylcholine acyl-alkyl C44:4 |
|  | PC_ae_C44_5 | Phosphatidylcholine acyl-alkyl C44:5 |
|  | PC_ae_C44_6 | Phosphatidylcholine acyl-alkyl C44:6 |
| Sphingomyelins | SM (OH) C14_1 | Hydroxysphingomyeline C14:1 |
|  | SM (OH) C16_1 | Hydroxysphingomyeline C16:1 |
|  | SM (OH) C22_1 | Hydroxysphingomyeline C22:1 |
|  | SM (OH) C22_2 | Hydroxysphingomyeline C22:2 |
|  | SM (OH) C24_1 | Hydroxysphingomyeline C24:1 |
|  | SM C16_0 | Sphingomyeline C16:0 |
|  | SM C16_1 | Sphingomyeline C16:1 |
|  | SM C18_0 | Sphingomyeline C18:0 |
|  | SM C18_1 | Sphingomyeline C18:1 |
|  | SM C20_2 | Sphingomyeline C20:2 |
|  | SM C24_0 | Sphingomyeline C24:0 |
|  | SM C24_1 | Sphingomyeline C24:1 |
| Hexoses | H1 | Hexoses |

**Supplementary Table 3** Coefficients of Variance (CV) of all metabolites in the *AbsoluteIDQ^TM^* p180 kit (BIOCRATES Life Sciences AG, Innsbruck, Austria)

| **Metabolite** | **CV of reference samples (%)** |
| --- | --- |
| C0 | 7.76 |
| C2 | 10.40 |
| C3 | 8.93 |
| C3-DC (C4-OH) | 13.20 |
| C3-OH | 42.36 |
| C3:1 | 32.67 |
| C4 | 12.43 |
| C4:1 | 17.20 |
| C5 | 12.89 |
| C5-DC (C6-OH) | 18.65 |
| C5-M-DC | 24.69 |
| C5-OH (C3-DC-M) | 19.70 |
| C5:1 | 18.68 |
| C5:1-DC | 24.27 |
| C6 (C4:1-DC) | 19.15 |
| C6:1 | 21.13 |
| C7-DC | 15.62 |
| C8 | 9.99 |
| C9 | 15.55 |
| C10 | 9.72 |
| C10:1 | 12.04 |
| C10:2 | 12.74 |
| C12 | 13.07 |
| C12-DC | 21.36 |
| C12:1 | 13.64 |
| C14 | 13.16 |
| C14:1 | 14.28 |
| C14:1-OH | 16.37 |
| C14:2 | 15.73 |
| C14:2-OH | 21.46 |
| C16 | 13.22 |
| C16-OH | 19.39 |
| C16:1 | 15.27 |
| C16:1-OH | 19.37 |
| C16:2 | 17.64 |
| C16:2-OH | 18.72 |
| C18 | 13.74 |
| C18:1 | 8.33 |
| C18:1-OH | 19.67 |
| C18:2 | 11.27 |
| Ala | 6.69 |
| Arg | 13.90 |
| Asn | 13.57 |
| Asp | 14.08 |
| Cit | 14.90 |
| Gln | 12.96 |
| Glu | 11.18 |
| Gly | 11.78 |
| His | 12.06 |
| Ile | 9.19 |
| Leu | 10.57 |
| Lys | 12.43 |
| Met | 12.57 |
| Orn | 12.01 |
| Phe | 10.79 |
| Pro | 10.06 |
| Ser | 13.22 |
| Thr | 9.38 |
| Trp | 11.83 |
| Tyr | 10.62 |
| Val | 9.86 |
| Ac-Orn | 21.42 |
| ADMA | 22.60 |
| alpha-AAA | 16.69 |
| c4-OH-Pro | 1033.90 |
| Carnosine | 91.23 |
| Creatinine | 6.09 |
| DOPA | 125.98 |
| Dopamine | 868.04 |
| Histamine | 101.73 |
| Kynurenine | 11.06 |
| Met-SO | 15.96 |
| Nitro-Tyr | #DIV/0! |
| PEA | 34.21 |
| Putrescine | 47.72 |
| SDMA | 23.49 |
| Serotonin | 63.00 |
| Spermidine | 9.85 |
| Spermine | 45.86 |
| t4-OH-Pro | 12.27 |
| Taurine | 7.83 |
| total DMA | 16.84 |
| lysoPC a C14:0 | 11.09 |
| lysoPC a C16:0 | 11.07 |
| lysoPC a C16:1 | 11.11 |
| lysoPC a C17:0 | 11.52 |
| lysoPC a C18:0 | 9.26 |
| lysoPC a C18:1 | 12.80 |
| lysoPC a C18:2 | 12.99 |
| lysoPC a C20:3 | 9.07 |
| lysoPC a C20:4 | 11.79 |
| lysoPC a C24:0 | 13.78 |
| lysoPC a C26:0 | 43.56 |
| lysoPC a C26:1 | 43.53 |
| lysoPC a C28:0 | 32.56 |
| lysoPC a C28:1 | 30.83 |
| PC aa C24:0 | 43.21 |
| PC aa C26:0 | 27.56 |
| PC aa C28:1 | 12.75 |
| PC aa C30:0 | 8.92 |
| PC aa C30:2 | 266.93 |
| PC aa C32:0 | 14.56 |
| PC aa C32:1 | 22.90 |
| PC aa C32:2 | 19.56 |
| PC aa C32:3 | 14.93 |
| PC aa C34:1 | 13.96 |
| PC aa C34:2 | 15.38 |
| PC aa C34:3 | 14.88 |
| PC aa C34:4 | 11.71 |
| PC aa C36:0 | 25.09 |
| PC aa C36:1 | 17.64 |
| PC aa C36:2 | 12.20 |
| PC aa C36:3 | 10.95 |
| PC aa C36:4 | 12.60 |
| PC aa C36:5 | 13.41 |
| PC aa C36:6 | 11.06 |
| PC aa C38:0 | 9.47 |
| PC aa C38:1 | 16.91 |
| PC aa C38:3 | 10.59 |
| PC aa C38:4 | 8.76 |
| PC aa C38:5 | 10.48 |
| PC aa C38:6 | 9.08 |
| PC aa C40:1 | 14.18 |
| PC aa C40:2 | 11.04 |
| PC aa C40:3 | 12.40 |
| PC aa C40:4 | 14.87 |
| PC aa C40:5 | 7.88 |
| PC aa C40:6 | 7.73 |
| PC aa C42:0 | 9.54 |
| PC aa C42:1 | 10.39 |
| PC aa C42:2 | 10.41 |
| PC aa C42:4 | 12.52 |
| PC aa C42:5 | 10.07 |
| PC aa C42:6 | 18.34 |
| PC ae C30:0 | 16.82 |
| PC ae C30:1 | 148.86 |
| PC ae C30:2 | 15.75 |
| PC ae C32:1 | 18.71 |
| PC ae C32:2 | 20.96 |
| PC ae C34:0 | 17.54 |
| PC ae C34:1 | 13.82 |
| PC ae C34:2 | 14.74 |
| PC ae C34:3 | 11.86 |
| PC ae C36:0 | 21.02 |
| PC ae C36:1 | 12.01 |
| PC ae C36:2 | 12.33 |
| PC ae C36:3 | 13.30 |
| PC ae C36:4 | 11.79 |
| PC ae C36:5 | 12.54 |
| PC ae C38:0 | 13.65 |
| PC ae C38:1 | 18.84 |
| PC ae C38:2 | 17.11 |
| PC ae C38:3 | 9.41 |
| PC ae C38:4 | 11.23 |
| PC ae C38:5 | 11.99 |
| PC ae C38:6 | 11.74 |
| PC ae C40:1 | 10.30 |
| PC ae C40:2 | 8.81 |
| PC ae C40:3 | 9.73 |
| PC ae C40:4 | 8.65 |
| PC ae C40:5 | 11.32 |
| PC ae C40:6 | 6.65 |
| PC ae C42:0 | 12.13 |
| PC ae C42:1 | 11.27 |
| PC ae C42:2 | 10.95 |
| PC ae C42:3 | 11.13 |
| PC ae C42:4 | 11.55 |
| PC ae C42:5 | 7.42 |
| PC ae C44:3 | 11.55 |
| PC ae C44:4 | 15.39 |
| PC ae C44:5 | 8.88 |
| PC ae C44:6 | 8.86 |
| SM (OH) C14:1 | 14.93 |
| SM (OH) C16:1 | 14.07 |
| SM (OH) C22:1 | 17.58 |
| SM (OH) C22:2 | 11.11 |
| SM (OH) C24:1 | 19.74 |
| SM C16:0 | 9.23 |
| SM C16:1 | 15.45 |
| SM C18:0 | 8.31 |
| SM C18:1 | 11.05 |
| SM C20:2 | 13.39 |
| SM C22:3 | 97.68 |
| SM C24:0 | 17.30 |
| SM C24:1 | 16.20 |
| SM C26:0 | 29.97 |
| SM C26:1 | 27.50 |
| H1 | 7.14 |

**Supplementary Table 4** Concentrations of the log (+1)-transformed non-standardized and plate-standardized concentrations of all metabolites given as mean (standard deviation).

| **Metabolite** | **Non-standardized concentrations in study sample** | **Plate-standardized concentrations in study sample** |
| --- | --- | --- |
| C0 | 3.562 (0.225) | 0 (0.96) |
| C2 | 2.038 (0.271) | 0 (0.96) |
| C3 | 0.325 (0.089) | 0 (0.96) |
| C3_DC__C4_OH_ | 0.047 (0.018) | 0 (0.96) |
| C4 | 0.197 (0.071) | 0 (0.96) |
| C4_1 | 0.036 (0.006) | 0 (0.96) |
| C5 | 0.134 (0.043) | 0 (0.96) |
| C7_DC | 0.046 (0.017) | 0 (0.96) |
| C8 | 0.202 (0.112) | 0 (0.96) |
| C9 | 0.035 (0.017) | 0 (0.96) |
| C10 | 0.279 (0.141) | 0 (0.96) |
| C10_1 | 0.128 (0.044) | 0 (0.96) |
| C12 | 0.136 (0.076) | 0 (0.96) |
| C12_1 | 0.128 (0.048) | 0 (0.96) |
| C14 | 0.047 (0.021) | 0 (0.96) |
| C14_1 | 0.093 (0.053) | 0 (0.96) |
| C14_1_OH | 0.016 (0.005) | 0 (0.96) |
| C14_2 | 0.031 (0.016) | 0 (0.96) |
| C14_2_OH | 0.009 (0.002) | 0 (0.96) |
| C16 | 0.12 (0.029) | 0 (0.96) |
| C16_1_OH | 0.011 (0.003) | 0 (0.96) |
| C16_2 | 0.011 (0.005) | 0 (0.96) |
| C18 | 0.053 (0.015) | 0 (0.96) |
| C18_1 | 0.134 (0.035) | 0 (0.96) |
| C18_2 | 0.043 (0.013) | 0 (0.96) |
| Ala | 5.859 (0.227) | 0 (0.96) |
| Arg | 4.725 (0.182) | 0 (0.96) |
| Asn | 3.794 (0.162) | 0 (0.96) |
| Asp | 3.203 (0.245) | 0 (0.96) |
| Cit | 3.493 (0.239) | 0 (0.96) |
| Gln | 6.336 (0.163) | 0 (0.96) |
| Glu | 4.172 (0.39) | 0 (0.96) |
| Gly | 5.543 (0.246) | 0 (0.96) |
| His | 4.432 (0.141) | 0 (0.96) |
| Ile | 4.282 (0.238) | 0 (0.96) |
| Leu | 4.932 (0.206) | 0 (0.96) |
| Lys | 5.003 (0.144) | 0 (0.96) |
| Met | 3.2 (0.205) | 0 (0.96) |
| Orn | 4.22 (0.213) | 0 (0.96) |
| Phe | 4.273 (0.159) | 0 (0.96) |
| Pro | 5.195 (0.258) | 0 (0.96) |
| Ser | 4.682 (0.201) | 0 (0.96) |
| Thr | 4.771 (0.2) | 0 (0.96) |
| Trp | 4.256 (0.189) | 0 (0.96) |
| Tyr | 4.263 (0.23) | 0 (0.96) |
| Val | 5.382 (0.179) | 0 (0.96) |
| Ac.Orn | 0.535 (0.279) | 0 (0.96) |
| ADMA | 0.406 (0.081) | 0 (0.96) |
| alpha.AAA | 0.435 (0.124) | 0 (0.96) |
| Creatinine | 4.377 (0.187) | 0 (0.96) |
| Kynurenine | 1.293 (0.183) | 0 (0.96) |
| Met.SO | 0.456 (0.1) | 0 (0.96) |
| SDMA | 0.472 (0.114) | 0 (0.96) |
| Spermidine | 0.087 (0.03) | 0 (0.96) |
| t4.OH.Pro | 2.304 (0.422) | 0 (0.96) |
| Taurine | 4.686 (0.186) | 0 (0.96) |
| total.DMA | 0.69 (0.103) | 0 (0.96) |
| lysoPC_a_C16_0 | 4.126 (0.218) | 0 (0.96) |
| lysoPC_a_C16_1 | 1.197 (0.226) | 0 (0.96) |
| lysoPC_a_C17_0 | 0.785 (0.154) | 0 (0.96) |
| lysoPC_a_C18_0 | 2.878 (0.237) | 0 (0.96) |
| lysoPC_a_C18_1 | 2.728 (0.293) | 0 (0.96) |
| lysoPC_a_C18_2 | 2.979 (0.328) | 0 (0.96) |
| lysoPC_a_C20_3 | 0.989 (0.193) | 0 (0.96) |
| lysoPC_a_C20_4 | 1.759 (0.267) | 0 (0.96) |
| PC_aa_C28_1 | 1.318 (0.181) | 0 (0.96) |
| PC_aa_C30_0 | 1.535 (0.248) | 0 (0.96) |
| PC_aa_C32_0 | 2.572 (0.197) | 0 (0.96) |
| PC_aa_C32_1 | 2.957 (0.422) | 0 (0.96) |
| PC_aa_C32_2 | 1.468 (0.332) | 0 (0.96) |
| PC_aa_C32_3 | 0.335 (0.074) | 0 (0.96) |
| PC_aa_C34_1 | 5.294 (0.238) | 0 (0.96) |
| PC_aa_C34_2 | 5.859 (0.196) | 0 (0.96) |
| PC_aa_C34_3 | 2.764 (0.272) | 0 (0.96) |
| PC_aa_C34_4 | 0.995 (0.222) | 0 (0.96) |
| PC_aa_C36_1 | 3.745 (0.256) | 0 (0.96) |
| PC_aa_C36_2 | 5.307 (0.217) | 0 (0.96) |
| PC_aa_C36_3 | 4.783 (0.215) | 0 (0.96) |
| PC_aa_C36_4 | 5.201 (0.249) | 0 (0.96) |
| PC_aa_C36_5 | 3.243 (0.412) | 0 (0.96) |
| PC_aa_C36_6 | 0.554 (0.171) | 0 (0.96) |
| PC_aa_C38_0 | 1.277 (0.201) | 0 (0.96) |
| PC_aa_C38_1 | 0.824 (0.14) | 0 (0.96) |
| PC_aa_C38_3 | 3.716 (0.253) | 0 (0.96) |
| PC_aa_C38_4 | 4.575 (0.268) | 0 (0.96) |
| PC_aa_C38_5 | 3.919 (0.249) | 0 (0.96) |
| PC_aa_C38_6 | 4.203 (0.289) | 0 (0.96) |
| PC_aa_C40_2 | 0.261 (0.058) | 0 (0.96) |
| PC_aa_C40_3 | 0.444 (0.083) | 0 (0.96) |
| PC_aa_C40_4 | 1.391 (0.206) | 0 (0.96) |
| PC_aa_C40_5 | 2.306 (0.253) | 0 (0.96) |
| PC_aa_C40_6 | 3.103 (0.304) | 0 (0.96) |
| PC_aa_C42_0 | 0.368 (0.09) | 0 (0.96) |
| PC_aa_C42_1 | 0.216 (0.052) | 0 (0.96) |
| PC_aa_C42_2 | 0.198 (0.042) | 0 (0.96) |
| PC_aa_C42_4 | 0.162 (0.031) | 0 (0.96) |
| PC_aa_C42_5 | 0.311 (0.077) | 0 (0.96) |
| PC_aa_C42_6 | 0.263 (0.071) | 0 (0.96) |
| PC_ae_C30_0 | 0.327 (0.08) | 0 (0.96) |
| PC_ae_C32_1 | 1.251 (0.159) | 0 (0.96) |
| PC_ae_C32_2 | 0.513 (0.094) | 0 (0.96) |
| PC_ae_C34_0 | 0.785 (0.142) | 0 (0.96) |
| PC_ae_C34_1 | 2.184 (0.196) | 0 (0.96) |
| PC_ae_C34_2 | 2.351 (0.257) | 0 (0.96) |
| PC_ae_C34_3 | 1.912 (0.266) | 0 (0.96) |
| PC_ae_C36_0 | 0.658 (0.1) | 0 (0.96) |
| PC_ae_C36_1 | 1.957 (0.201) | 0 (0.96) |
| PC_ae_C36_2 | 2.472 (0.246) | 0 (0.96) |
| PC_ae_C36_3 | 1.917 (0.221) | 0 (0.96) |
| PC_ae_C36_4 | 2.756 (0.266) | 0 (0.96) |
| PC_ae_C36_5 | 2.482 (0.252) | 0 (0.96) |
| PC_ae_C38_0 | 1.133 (0.214) | 0 (0.96) |
| PC_ae_C38_1 | 0.454 (0.173) | 0 (0.96) |
| PC_ae_C38_2 | 0.82 (0.18) | 0 (0.96) |
| PC_ae_C38_3 | 1.397 (0.176) | 0 (0.96) |
| PC_ae_C38_4 | 2.429 (0.21) | 0 (0.96) |
| PC_ae_C38_5 | 2.778 (0.221) | 0 (0.96) |
| PC_ae_C38_6 | 2.023 (0.228) | 0 (0.96) |
| PC_ae_C40_1 | 0.901 (0.15) | 0 (0.96) |
| PC_ae_C40_2 | 0.91 (0.148) | 0 (0.96) |
| PC_ae_C40_3 | 0.641 (0.099) | 0 (0.96) |
| PC_ae_C40_4 | 1.119 (0.145) | 0 (0.96) |
| PC_ae_C40_5 | 1.28 (0.156) | 0 (0.96) |
| PC_ae_C40_6 | 1.506 (0.205) | 0 (0.96) |
| PC_ae_C42_1 | 0.307 (0.061) | 0 (0.96) |
| PC_ae_C42_2 | 0.392 (0.075) | 0 (0.96) |
| PC_ae_C42_3 | 0.535 (0.102) | 0 (0.96) |
| PC_ae_C42_4 | 0.504 (0.111) | 0 (0.96) |
| PC_ae_C42_5 | 1.059 (0.133) | 0 (0.96) |
| PC_ae_C44_3 | 0.112 (0.024) | 0 (0.96) |
| PC_ae_C44_4 | 0.281 (0.061) | 0 (0.96) |
| PC_ae_C44_5 | 0.944 (0.167) | 0 (0.96) |
| PC_ae_C44_6 | 0.696 (0.148) | 0 (0.96) |
| SM (OH) C14_1 | 2.001 (0.244) | 0 (0.96) |
| SM (OH) C16_1 | 1.448 (0.211) | 0 (0.96) |
| SM (OH) C22_1 | 2.505 (0.235) | 0 (0.96) |
| SM (OH) C22_2 | 2.412 (0.246) | 0 (0.96) |
| SM (OH) C24_1 | 0.744 (0.14) | 0 (0.96) |
| SM C16_0 | 4.679 (0.197) | 0 (0.96) |
| SM C16_1 | 2.894 (0.209) | 0 (0.96) |
| SM C18_0 | 3.141 (0.221) | 0 (0.96) |
| SM C18_1 | 2.473 (0.242) | 0 (0.96) |
| SM C20_2 | 0.446 (0.094) | 0 (0.96) |
| SM C24_0 | 2.912 (0.219) | 0 (0.96) |
| SM C24_1 | 3.942 (0.205) | 0 (0.96) |
| H1 | 8.497 (0.191) | 0 (0.96) |

**Supplemental Information 1:** Information on determination of the number of clusters

Based on similarities in concentrations of metabolites, individuals were segregated into different clusters. To find the optimal number of clusters we calculated the Calinski-Harabasz (CH) index (1) for 3 – 8 clusters. The CH index is based on the sum of squares between cluster variance and within cluster variance. It is calculated by dividing the between cluster variance per number of clusters by the within cluster variance. Hence, the number of clusters leading to the highest CH index fits best to the data.

Additionally, we determined the optimal number of clusters graphically using the within-cluster sum of squares (WSS) and silhouette method. The WSS plot shows the within-cluster sum of squares on the y axis and each cluster on the x axis. The optimal number of clusters is determined where no great change in the slope appears, meaning an additional cluster does not further decrease within-cluster variance. The silhouette method measures the distance between clusters by examining the distance of each data point to all other datapoints within the cluster and between the clusters. Hence, the silhouette score is the average of all silhouette coefficients, calculated by subtracting the average within cluster distance from the between cluster distance and dividing that by the maximum of both.

Afterwards, to identify an underlying structure in the data the unsupervised machine learning method k-means clustering with Hartigan-Wong algorithm (2) was used. K-means clustering assigns each individual randomly to one cluster and calculates the centroid as the mean concentration. Next, the Euclidean distance of each value to all clusters centroids is calculated and individuals are reassigned to the nearest cluster. This step is done iteratively until the smallest within cluster sum of squares is achieved (1).

**Supplementary Table 5** Calinski-Harabasz index for 3-8 clusters in the main exposure (left ventricle parameters) data set. According to the highest index at 54.60 three clusters fit best to the data.

| **Clusters** | **CH – index** |
| --- | --- |
| 3 | 54.60 |
| 4 | 43.80 |
| 5 | 36.81 |
| 6 | 32.24 |
| 7 | 28.85 |
| 8 | 26.08 |


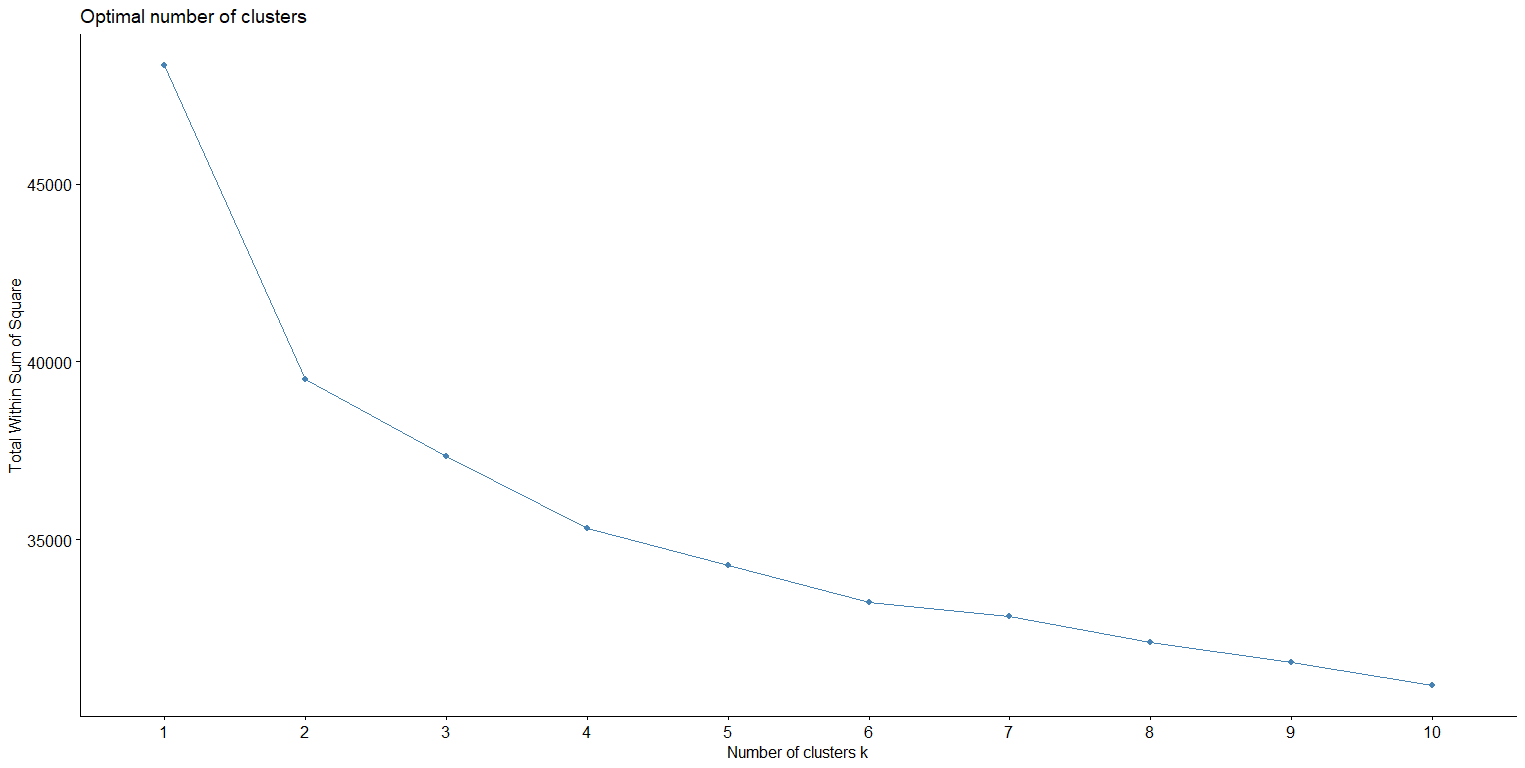


**Supplementary Figure 1** Within cluster sum of squares method. The y axis shows the number of clusters and the y axis the WSS. The optimal number of clusters is where the WSS difference is much anymore. The graph shows potential number of clusters at 3 – 4 cluster.


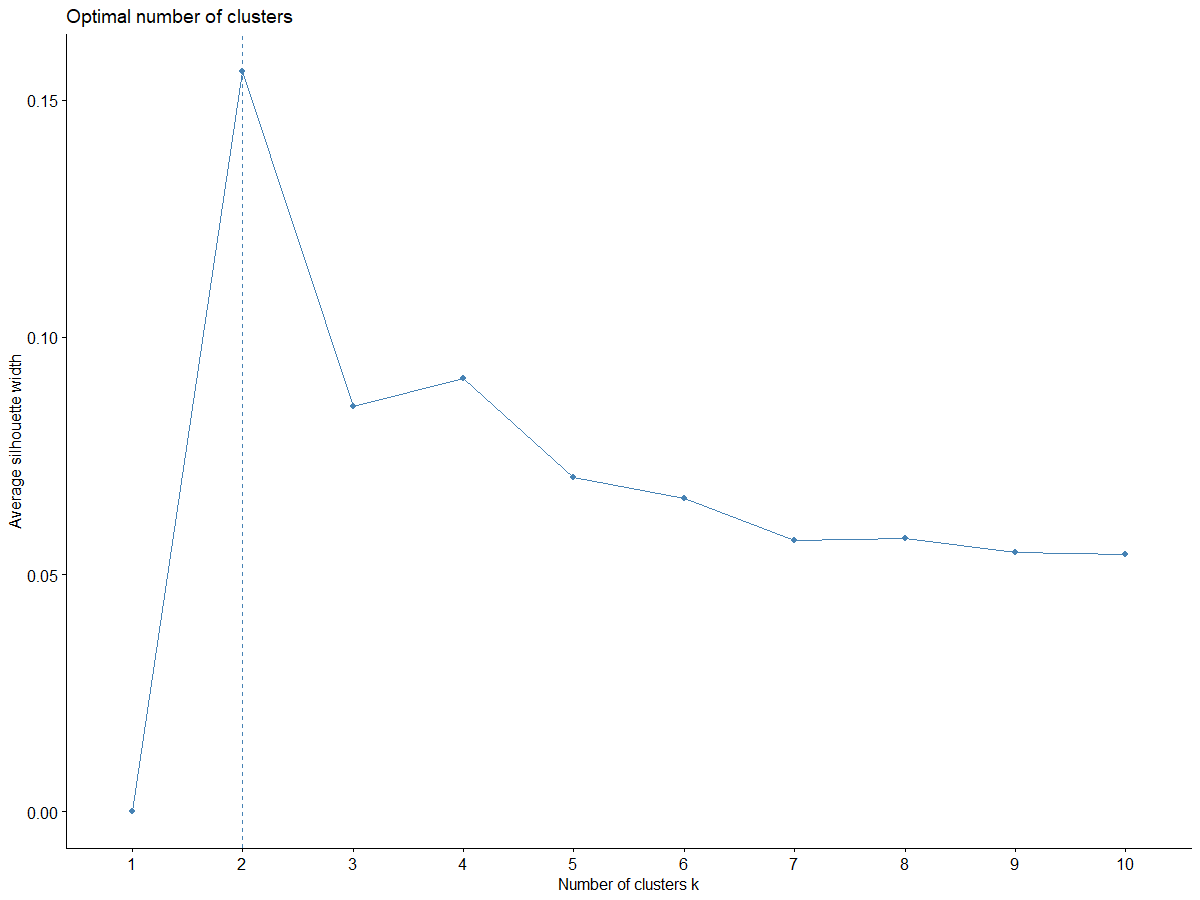


**Supplementary Figure 2** Graphical assessment of the silhouette method with the number of clusters on the x axis and the average silhouette coefficient on the y axis. This method indicates 2 clusters fit best to the data.

**Supplementary Table 6** Description of individuals included in or excluded from the secondary exposure (carotid plaque) data set. Mean (SD) was calculated for continuous variables and frequency (proportion) for categorical values. P-value from t-test or χ^2^test, where applicable.

| **Variable** | **Included**  **(N = 256)** | **Excluded**  **(N = 143)** | **p-value** |
| --- | --- | --- | --- |
| Age (years) | 56.2 (9.1) | 56.7 (9.4) | 0.605 |
| Female sex | 96 (37.5%) | 73 (51.0%) | **0.009** |
| BMI (kg/m^2^) | 27.6 (4.3) | 29.0 (5.7) | **0.006** |
| Regularly physically active | 153 (59.7%) | 84 (58.8%) | 0.810 |
| Smoking status |  |  | 0.838 |
| Currently smoking | 53 (20.7%) | 27 (18.9%) |  |
| Ex - Smoker | 109 (42.6%) | 65 (45.5%) |  |
| Never - Smoker | 94 (36.7%) | 51 (35.7%) |  |
| Alcohol consumption (g/Day) | 17.5 (22.2) | 19.8 (26.3) | 0.369 |
| Diabetes status (based on OGTT) |  |  | **0.001** |
| Normoglycemic | 165 (64.5%) | 77 (53.8%) |  |
| Prediabetes | 51 (19.9%) | 52 (36.4%) |  |
| Diabetes | 40 (15.6%) | 14 (9.8%) |  |
| Fasting glucose (mg/dl) | 105.4 (25.7) | 102.1 (15.4) | 0.162 |
| Hypertension | 82 (32.0%) | 54 (37.8%) | 0.247 |
| Systolic blood pressure (mmHg) | 120.3 (17.0) | 121.3 (16.4) | 0.565 |
| Diastolic blood pressure (mmHg) | 75.1 (10.0) | 75.6 (10.0) | 0.659 |
| Angina pectoris | 19 (7.4%) | 6 (4.3%) | 0.214 |
| Total cholesterol (mg/dl) | 217.3 (36.1) | 219.0 (36.7) | 0.659 |
| HDL cholesterol (mg/dl) | 60.9 (17.4) | 63.6 (18.0) | 0.151 |
| LDL cholesterol (mg/dl) | 140.0 (33.1) | 138.9 (32.7) | 0.764 |
| Triglycerides (mg/dl) | 135.2 (91.2) | 125.4 (72.1) | 0.268 |
| hsCRP | 1.07 (0.52; 2.34) | 1.39 (0.85;2.91) | 0.022 |
| Antidiabetic drugs | 26 (10.2%) | 6 (4.2%) | 0.036 |
| Antihypertensive drugs | 62 (24.2%) | 40 (28.0%) | 0.410 |
| Lipid lowering drug | 29 (11.3%) | 14 (9.8%) | 0.635 |
| Anticoagulant drugs | 5 (2.0%) | n <5 | 0.921 |
| Antiplatelet drugs | 10 (3.9%) | 5 (3.5%) | 0.837 |
| SCORE2, % | 5.2 (3.7) | 5.1 (4.0) | 0.798 |
| **MRI variables** |  |  |  |
| Any plaque present | 54 (21.1%) | n <5 | 0.556 |
| Plaque index |  |  | 0.963 |
| Normal – diffuse thickness | 202 (78.9%) | 7 (87.5%) |  |
| Plaque – complex plaque | 38 (14.8%) | n <5 |  |
| Fibrotic plaque | 15 (6%) | n <5 |  |
| Normalized wall index (%) * | 0.4 (0.0) | 0.4 (0.0) | 0.748 |
| **Metabolites (µmol/L)** |  |  |  |
| Ala | 364.5 (81.5) | 350.6 (77.8) | 0.105 |
| Arg | 112.7 (20.4) | 114.4 (21.1) | 0.438 |
| Asn | 44.4 (7.7) | 42.8 (6.7) | 0.044 |
| Asp | 23.4 (5.8) | 26.0 (7.8) | **< 0.001** |
| Cit | 32.8 (8.3) | 31.7 (8.0) | 0.212 |
| Gln | 574.1 (92.6) | 557.3 (91.8) | 0.090 |
| Glu | 69.0 (26.3) | 69.4 (25.0) | 0.871 |
| Gly | 266.7 (74.2) | 253.0 (62.5) | 0.069 |
| His | 85.0 (11.9) | 81.4 (11.4) | **0.005** |
| Ile | 74.4 (18.3) | 72.3 (18.3) | 0.294 |
| Leu | 141.4 (28.9) | 138.6 (29.6) | 0.371 |
| Lys | 150.7 (20.1) | 146.3 (24.3) | 0.060 |
| Met | 24.1 (4.8) | 23.9 (5.9) | 0.724 |
| Orn | 68.6 (13.5) | 67.4 (15.6) | 0.432 |
| Phe | 71.2 (11.1) | 72.5 (12.6) | 0.297 |
| Pro | 188.2 (48.1) | 180.6 (50.4) | 0.144 |
| Ser | 109.3 (22.3) | 107.9 (21.5) | 0.564 |
| Thr | 120.3 (22.8) | 116.2 (25.7) | 0.108 |
| Trp | 70.7 (13.3) | 70.1 (13.7) | 0.669 |
| Tyr | 71.3 (15.8) | 72.1 (17.8) | 0.653 |
| Val | 220.6 (39.2) | 217.9 (37.7) | 0.509 |
| Ac.Orn | 0.8 (0.7) | 0.8 (0.6) | 0.433 |
| ADMA | 0.5 (0.1) | 0.5 (0.1) | 0.948 |
| alpha.AAA | 0.6 (0.2) | 0.5 (0.2) | 0.247 |
| Creatinine | 81.4 (14.5) | 77.4 (15.1) | **0.010** |
| Kynurenine | 2.7 (0.7) | 2.7 (0.7) | 0.998 |
| Met.SO | 0.6 (0.1) | 0.6 (0.2) | **0.004** |
| SDMA | 0.6 (0.2) | 0.6 (0.2) | **0.003** |
| Spermidine | 0.1 (0.0) | 0.1 (0.0) | 0.635 |
| t4.OH.Pro | 10.3 (5.5) | 9.4 (5.4) | 0.104 |
| Taurine | 110.2 (20.9) | 107.6 (19.3) | 0.242 |
| total.DMA | 1.0 (0.2) | 1.0 (0.2) | 0.100 |
| C0 | 35.4 (8.1) | 35.3 (7.8) | 0.833 |
| C2 | 7.1 (2.4) | 6.9 (2.2) | 0.424 |
| C3 | 0.4 (0.1) | 0.4 (0.1) | 0.281 |
| C3_DC_C4_OH | 0.0 (0.0) | 0.0 (0.0) | 0.514 |
| C4 | 0.2 (0.1) | 0.2 (0.1) | 0.629 |
| C4_1 | 0.0 (0.0) | 0.0 (0.0) | 0.115 |
| C5 | 0.1 (0.1) | 0.1 (0.0) | 0.958 |
| C7_DC | 0.0 (0.0) | 0.0 (0.0) | 0.340 |
| C8 | 0.2 (0.1) | 0.2 (0.3) | 0.594 |
| C9 | 0.0 (0.0) | 0.0 (0.0) | 0.487 |
| C10 | 0.3 (0.2) | 0.3 (0.5) | 0.849 |
| C10_1 | 0.1 (0.0) | 0.1 (0.1) | 0.988 |
| C12 | 0.1 (0.1) | 0.2 (0.2) | 0.817 |
| C12_1 | 0.1 (0.1) | 0.1 (0.1) | 0.682 |
| C14 | 0.0 (0.0) | 0.0 (0.0) | 0.696 |
| C14_1 | 0.1 (0.0) | 0.1 (0.1) | 0.999 |
| C14_1_OH | 0.0 (0.0) | 0.0 (0.0) | 0.213 |
| C14_2 | 0.0 (0.0) | 0.0 (0.0) | 0.816 |
| C14_2_OH | 0.0 (0.0) | 0.0 (0.0) | 0.472 |
| C16 | 0.1 (0.0) | 0.1 (0.0) | 0.357 |
| C16_1_OH | 0.0 (0.0) | 0.0 (0.0) | 0.179 |
| C16_2 | 0.0 (0.0) | 0.0 (0.0) | 0.990 |
| C18 | 0.1 (0.0) | 0.1 (0.0) | **0.008** |
| C18_1 | 0.1 (0.0) | 0.1 (0.0) | 0.266 |
| C18_2 | 0.0 (0.0) | 0.0 (0.0) | 0.194 |
| lysoPC_a_C16_0 | 62.8 (12.3) | 61.4 (14.7) | 0.334 |
| lysoPC_a_C16_1 | 2.4 (0.9) | 2.4 (0.9) | 0.570 |
| lysoPC_a_C17_0 | 1.2 (0.3) | 1.2 (0.3) | 0.222 |
| lysoPC_a_C18_0 | 17.3 (4.0) | 16.9 (4.3) | 0.272 |
| lysoPC_a_C18_1 | 15.2 (4.9) | 14.6 (4.6) | 0.276 |
| lysoPC_a_C18_2 | 20.0 (7.1) | 18.7 (7.1) | 0.088 |
| lysoPC_a_C20_3 | 1.8 (0.6) | 1.7 (0.5) | 0.113 |
| lysoPC_a_C20_4 | 5.2 (1.7) | 4.7 (1.7) | **0.021** |
| PC_aa_C28_1 | 2.8 (0.7) | 2.8 (0.7) | 0.513 |
| PC_aa_C30_0 | 3.8 (1.2) | 3.8 (1.3) | 0.911 |
| PC_aa_C32_0 | 12.5 (2.7) | 12.2 (2.7) | 0.266 |
| PC_aa_C32_1 | 20.0 (9.4) | 21.2 (10.8) | 0.274 |
| PC_aa_C32_2 | 3.7 (1.5) | 3.3 (1.5) | **0.016** |
| PC_aa_C32_3 | 0.4 (0.1) | 0.4 (0.1) | 0.371 |
| PC_aa_C34_1 | 208.3 (50.3) | 201.3 (51.5) | 0.201 |
| PC_aa_C34_2 | 361.7 (66.5) | 345.9 (68.4) | **0.028** |
| PC_aa_C34_3 | 15.7 (4.6) | 15.1 (4.2) | 0.260 |
| PC_aa_C34_4 | 1.8 (0.7) | 1.7 (0.6) | **0.008** |
| PC_aa_C36_1 | 43.3 (11.5) | 42.4 (11.4) | 0.465 |
| PC_aa_C36_2 | 208.1 (43.1) | 200.5 (43.3) | 0.099 |
| PC_aa_C36_3 | 122.9 (25.6) | 118.1 (25.3) | 0.077 |
| PC_aa_C36_4 | 192.1 (46.3) | 177.5 (46.7) | **0.003** |
| PC_aa_C36_5 | 28.2 (13.5) | 25.5 (9.9) | **0.050** |
| PC_aa_C36_6 | 0.8 (0.3) | 0.7 (0.3) | **0.033** |
| PC_aa_C38_0 | 2.7 (0.7) | 2.6 (0.8) | 0.185 |
| PC_aa_C38_1 | 1.3 (0.3) | 1.3 (0.3) | 0.193 |
| PC_aa_C38_3 | 41.7 (10.2) | 40.9 (10.3) | 0.445 |
| PC_aa_C38_4 | 102.2 (26.6) | 95.6 (26.9) | **0.020** |
| PC_aa_C38_5 | 52.6 (13.4) | 48.7 (12.5) | **0.005** |
| PC_aa_C38_6 | 71.6 (19.8) | 65.4 (20.6) | **0.004** |
| PC_aa_C40_2 | 0.3 (0.1) | 0.3 (0.1) | 0.216 |
| PC_aa_C40_3 | 0.6 (0.1) | 0.6 (0.1) | 0.095 |
| PC_aa_C40_4 | 3.2 (0.9) | 3.1 (0.9) | 0.284 |
| PC_aa_C40_5 | 9.6 (2.7) | 9.2 (2.9) | 0.133 |
| PC_aa_C40_6 | 23.0 (7.2) | 21.7 (7.8) | 0.104 |
| PC_aa_C42_0 | 0.5 (0.1) | 0.4 (0.1) | 0.255 |
| PC_aa_C42_1 | 0.2 (0.1) | 0.2 (0.1) | 0.133 |
| PC_aa_C42_2 | 0.2 (0.1) | 0.2 (0.1) | 0.631 |
| PC_aa_C42_4 | 0.2 (0.0) | 0.2 (0.0) | 0.185 |
| PC_aa_C42_5 | 0.4 (0.1) | 0.4 (0.1) | 0.219 |
| PC_aa_C42_6 | 0.3 (0.1) | 0.3 (0.1) | 0.157 |
| PC_ae_C30_0 | 0.4 (0.1) | 0.4 (0.1) | 0.807 |
| PC_ae_C32_1 | 2.5 (0.6) | 2.6 (0.6) | 0.872 |
| PC_ae_C32_2 | 0.7 (0.2) | 0.7 (0.2) | 0.252 |
| PC_ae_C34_0 | 1.2 (0.3) | 1.2 (0.3) | 0.651 |
| PC_ae_C34_1 | 8.1 (1.8) | 8.1 (1.8) | 0.949 |
| PC_ae_C34_2 | 9.9 (2.7) | 9.7 (2.7) | 0.408 |
| PC_ae_C34_3 | 6.0 (1.9) | 5.9 (1.9) | 0.638 |
| PC_ae_C36_0 | 1.0 (0.2) | 0.9 (0.2) | 0.305 |
| PC_ae_C36_1 | 6.3 (1.5) | 6.2 (1.4) | 0.431 |
| PC_ae_C36_2 | 11.3 (3.0) | 10.8 (2.8) | 0.129 |
| PC_ae_C36_3 | 6.0 (1.5) | 5.8 (1.5) | 0.320 |
| PC_ae_C36_4 | 15.6 (4.3) | 14.8 (4.1) | 0.100 |
| PC_ae_C36_5 | 11.6 (3.1) | 10.9 (3.0) | **0.039** |
| PC_ae_C38_0 | 2.3 (0.8) | 2.1 (0.6) | **0.009** |
| PC_ae_C38_1 | 0.6 (0.2) | 0.5 (0.3) | **< 0.001** |
| PC_ae_C38_2 | 1.3 (0.4) | 1.3 (0.4) | 0.603 |
| PC_ae_C38_3 | 3.1 (0.7) | 3.1 (0.7) | 0.929 |
| PC_ae_C38_4 | 10.8 (2.3) | 10.2 (2.3) | **0.019** |
| PC_ae_C38_5 | 15.8 (3.4) | 15.0 (3.7) | 0.056 |
| PC_ae_C38_6 | 6.9 (1.7) | 6.5 (1.7) | **0.042** |
| PC_ae_C40_1 | 1.5 (0.4) | 1.4 (0.3) | **0.001** |
| PC_ae_C40_2 | 1.5 (0.4) | 1.5 (0.4) | 0.380 |
| PC_ae_C40_3 | 0.9 (0.2) | 0.9 (0.2) | 0.614 |
| PC_ae_C40_4 | 2.1 (0.4) | 2.0 (0.5) | 0.148 |
| PC_ae_C40_5 | 2.7 (0.5) | 2.6 (0.6) | 0.309 |
| PC_ae_C40_6 | 3.7 (1.0) | 3.4 (0.9) | **0.013** |
| PC_ae_C42_1 | 0.4 (0.1) | 0.4 (0.1) | 0.233 |
| PC_ae_C42_2 | 0.5 (0.1) | 0.5 (0.1) | 0.303 |
| PC_ae_C42_3 | 0.7 (0.2) | 0.7 (0.2) | 0.167 |
| PC_ae_C42_4 | 0.7 (0.2) | 0.6 (0.2) | 0.237 |
| PC_ae_C42_5 | 1.9 (0.4) | 1.9 (0.4) | 0.359 |
| PC_ae_C44_3 | 0.1 (0.0) | 0.1 (0.0) | 0.792 |
| PC_ae_C44_4 | 0.3 (0.1) | 0.3 (0.1) | 0.351 |
| PC_ae_C44_5 | 1.6 (0.4) | 1.6 (0.5) | 0.152 |
| PC_ae_C44_6 | 1.0 (0.3) | 1.0 (0.3) | 0.163 |
| SM__OH__C14_1 | 6.6 (1.9) | 6.5 (1.8) | 0.656 |
| SM__OH__C16_1 | 3.4 (1.0) | 3.3 (0.9) | 0.422 |
| SM__OH__C22_1 | 11.7 (2.8) | 11.3 (3.0) | 0.205 |
| SM__OH__C22_2 | 10.5 (2.8) | 10.3 (2.8) | 0.491 |
| SM__OH__C24_1 | 1.1 (0.3) | 1.1 (0.3) | 0.458 |
| SM_C16_0 | 109.2 (20.0) | 107.4 (21.6) | 0.401 |
| SM_C16_1 | 17.4 (3.6) | 17.7 (4.2) | 0.547 |
| SM_C18_0 | 22.9 (5.0) | 22.4 (5.7) | 0.411 |
| SM_C18_1 | 11.2 (2.8) | 11.3 (3.4) | 0.746 |
| SM_C20_2 | 0.6 (0.2) | 0.6 (0.2) | 0.132 |
| SM_C24_0 | 18.0 (3.7) | 17.3 (4.2) | 0.117 |
| SM_C24_1 | 52.2 (9.6) | 50.7 (11.6) | 0.164 |
| H1 | 5101.4 (1251.9) | 4907.7 (991.0) | 0.121 |
| *% of total vessel diameter covered by wall |  |  |  |

Metabolite concentrations per cluster

**Supplementary Table 7** Metabolite concentrations (µmol/L) per cluster of the main exposure (left ventricle parameters) data set. ANOVA was used to test for differences between clusters. A p-value ≤ 0.05 was considered significant and is marked bold.

| **Metabolite** | **Cluster 1**  **(N = 116)** | **Cluster 2**  **(N = 106)** | **Cluster 3**  **(N = 138)** | **Total**  **(N = 360)** | **p value** |
| --- | --- | --- | --- | --- | --- |
| Ala | 384.35 (71.74) | 328.66 (74.14) | 359.41 (84.50) | 358.39 (80.37) | **< 0.001** |
| Arg | 119.52 (21.11) | 115.03 (19.96) | 107.53 (19.56) | 113.60 (20.77) | **< 0.001** |
| Asn | 45.31 (8.01) | 44.19 (7.43) | 42.83 (6.82) | 44.03 (7.45) | **0.029** |
| Asp | 25.59 (7.55) | 24.91 (5.73) | 22.94 (5.98) | 24.37 (6.55) | **0.003** |
| Cit | 33.38 (7.44) | 34.13 (7.60) | 31.35 (8.65) | 32.82 (8.04) | **0.018** |
| Gln | 601.92 (97.21) | 573.50 (87.59) | 543.57 (86.32) | 571.18 (93.35) | **< 0.001** |
| Glu | 74.45 (24.37) | 56.64 (20.82) | 73.15 (27.42) | 68.71 (25.79) | **< 0.001** |
| Gly | 258.51 (50.18) | 290.35 (81.53) | 244.58 (67.08) | 262.55 (69.47) | **< 0.001** |
| His | 88.40 (10.21) | 83.37 (11.37) | 80.70 (12.40) | 83.97 (11.85) | **< 0.001** |
| Ile | 81.70 (17.68) | 62.49 (11.85) | 75.04 (18.29) | 73.49 (18.09) | **< 0.001** |
| Leu | 155.23 (28.96) | 123.86 (21.28) | 141.26 (28.50) | 140.64 (29.38) | **< 0.001** |
| Lys | 156.94 (19.61) | 142.69 (20.12) | 148.13 (22.33) | 149.37 (21.54) | **< 0.001** |
| Met | 26.25 (5.54) | 22.58 (4.75) | 23.33 (4.87) | 24.05 (5.28) | **< 0.001** |
| Orn | 73.23 (14.14) | 64.63 (12.00) | 67.61 (15.37) | 68.55 (14.44) | **< 0.001** |
| Phe | 77.97 (11.34) | 68.62 (10.74) | 68.75 (10.61) | 71.68 (11.69) | **< 0.001** |
| Pro | 202.55 (51.59) | 169.47 (45.27) | 183.44 (45.15) | 185.49 (49.00) | **< 0.001** |
| Ser | 107.07 (22.21) | 114.23 (21.72) | 106.94 (21.53) | 109.13 (22.00) | **0.017** |
| Thr | 121.88 (23.32) | 121.83 (23.60) | 115.30 (24.24) | 119.34 (23.91) | **0.041** |
| Trp | 76.22 (13.36) | 68.05 (12.72) | 68.27 (12.88) | 70.77 (13.49) | **< 0.001** |
| Tyr | 80.58 (15.83) | 66.39 (12.94) | 68.72 (16.73) | 71.85 (16.53) | **< 0.001** |
| Val | 236.14 (36.36) | 199.98 (32.14) | 221.76 (38.70) | 219.98 (38.74) | **< 0.001** |
| Ac.Orn | 0.94 (0.76) | 0.75 (0.61) | 0.68 (0.44) | 0.78 (0.62) | **0.003** |
| ADMA | 0.54 (0.13) | 0.49 (0.10) | 0.49 (0.13) | 0.51 (0.12) | **0.002** |
| alpha.AAA | 0.62 (0.20) | 0.44 (0.13) | 0.59 (0.21) | 0.56 (0.20) | **< 0.001** |
| Creatinine | 86.71 (14.20) | 76.17 (14.01) | 77.28 (14.19) | 79.99 (14.85) | **< 0.001** |
| Kynurenine | 2.95 (0.74) | 2.64 (0.70) | 2.55 (0.61) | 2.71 (0.70) | **< 0.001** |
| Met.SO | 0.61 (0.19) | 0.56 (0.17) | 0.58 (0.16) | 0.59 (0.17) | 0.089 |
| SDMA | 0.66 (0.22) | 0.62 (0.18) | 0.57 (0.16) | 0.61 (0.19) | **< 0.001** |
| Spermidine | 0.10 (0.04) | 0.09 (0.03) | 0.08 (0.03) | 0.09 (0.03) | **< 0.001** |
| t4.OH.Pro | 10.95 (4.91) | 8.47 (3.89) | 10.45 (6.67) | 10.03 (5.49) | **0.002** |
| Taurine | 111.49 (18.82) | 112.10 (20.41) | 105.39 (20.92) | 109.33 (20.30) | **0.014** |
| total.DMA | 1.07 (0.23) | 1.01 (0.21) | 0.95 (0.18) | 1.00 (0.21) | **< 0.001** |
| C0 | 39.26 (7.42) | 31.82 (6.91) | 34.14 (7.65) | 35.11 (7.94) | **< 0.001** |
| C2 | 8.27 (2.48) | 6.14 (1.73) | 6.51 (2.00) | 6.97 (2.28) | **< 0.001** |
| C3 | 0.44 (0.12) | 0.33 (0.10) | 0.39 (0.14) | 0.39 (0.13) | **< 0.001** |
| C3_DC__C4_OH_ | 0.05 (0.02) | 0.04 (0.01) | 0.05 (0.02) | 0.05 (0.02) | **< 0.001** |
| C4 | 0.25 (0.10) | 0.20 (0.08) | 0.21 (0.08) | 0.22 (0.09) | **< 0.001** |
| C4_1 | 0.04 (0.01) | 0.04 (0.01) | 0.04 (0.01) | 0.04 (0.01) | **< 0.001** |
| C5 | 0.17 (0.04) | 0.12 (0.06) | 0.14 (0.04) | 0.14 (0.05) | **< 0.001** |
| C7_DC | 0.06 (0.02) | 0.04 (0.01) | 0.04 (0.01) | 0.05 (0.02) | **< 0.001** |
| C8 | 0.32 (0.35) | 0.20 (0.07) | 0.18 (0.06) | 0.23 (0.22) | **< 0.001** |
| C9 | 0.04 (0.02) | 0.04 (0.02) | 0.03 (0.01) | 0.04 (0.02) | **< 0.001** |
| C10 | 0.48 (0.54) | 0.29 (0.11) | 0.26 (0.09) | 0.34 (0.33) | **< 0.001** |
| C10_1 | 0.17 (0.07) | 0.13 (0.04) | 0.12 (0.03) | 0.14 (0.05) | **< 0.001** |
| C12 | 0.21 (0.20) | 0.13 (0.04) | 0.12 (0.03) | 0.15 (0.13) | **< 0.001** |
| C12_1 | 0.18 (0.08) | 0.12 (0.03) | 0.12 (0.03) | 0.14 (0.06) | **< 0.001** |
| C14 | 0.06 (0.04) | 0.04 (0.01) | 0.04 (0.01) | 0.05 (0.02) | **< 0.001** |
| C14_1 | 0.14 (0.11) | 0.08 (0.03) | 0.08 (0.03) | 0.10 (0.07) | **< 0.001** |
| C14_1_OH | 0.02 (0.01) | 0.02 (0.00) | 0.01 (0.00) | 0.02 (0.00) | **< 0.001** |
| C14_2 | 0.04 (0.02) | 0.03 (0.01) | 0.03 (0.01) | 0.03 (0.02) | **< 0.001** |
| C14_2_OH | 0.01 (0.00) | 0.01 (0.00) | 0.01 (0.00) | 0.01 (0.00) | **< 0.001** |
| C16 | 0.15 (0.03) | 0.12 (0.02) | 0.11 (0.03) | 0.13 (0.03) | **< 0.001** |
| C16_1_OH | 0.01 (0.00) | 0.01 (0.00) | 0.01 (0.00) | 0.01 (0.00) | **< 0.001** |
| C16_2 | 0.01 (0.01) | 0.01 (0.00) | 0.01 (0.00) | 0.01 (0.00) | **< 0.001** |
| C18 | 0.06 (0.02) | 0.05 (0.01) | 0.05 (0.01) | 0.05 (0.02) | **< 0.001** |
| C18_1 | 0.17 (0.04) | 0.13 (0.03) | 0.13 (0.03) | 0.14 (0.04) | **< 0.001** |
| C18_2 | 0.05 (0.01) | 0.04 (0.01) | 0.04 (0.01) | 0.04 (0.01) | **< 0.001** |
| lysoPC_a_C16_0 | 68.61 (12.96) | 62.65 (12.77) | 56.88 (11.72) | 62.36 (13.35) | **< 0.001** |
| lysoPC_a_C16_1 | 2.78 (1.01) | 2.31 (0.61) | 2.14 (0.74) | 2.40 (0.85) | **< 0.001** |
| lysoPC_a_C17_0 | 1.25 (0.32) | 1.40 (0.34) | 1.05 (0.27) | 1.22 (0.34) | **< 0.001** |
| lysoPC_a_C18_0 | 18.46 (4.03) | 18.27 (4.19) | 15.46 (3.61) | 17.25 (4.16) | **< 0.001** |
| lysoPC_a_C18_1 | 16.26 (5.27) | 15.37 (4.76) | 13.60 (4.16) | 14.98 (4.84) | **< 0.001** |
| lysoPC_a_C18_2 | 20.28 (6.48) | 21.15 (8.05) | 18.30 (6.79) | 19.78 (7.18) | **0.005** |
| lysoPC_a_C20_3 | 1.97 (0.59) | 1.66 (0.49) | 1.61 (0.52) | 1.74 (0.56) | **< 0.001** |
| lysoPC_a_C20_4 | 5.71 (1.79) | 4.71 (1.45) | 4.68 (1.49) | 5.02 (1.65) | **< 0.001** |
| PC_aa_C28_1 | 2.97 (0.62) | 3.26 (0.67) | 2.31 (0.46) | 2.80 (0.71) | **< 0.001** |
| PC_aa_C30_0 | 4.24 (1.17) | 4.17 (1.23) | 3.11 (0.92) | 3.79 (1.22) | **< 0.001** |
| PC_aa_C32_0 | 13.93 (2.35) | 12.96 (2.19) | 10.53 (1.90) | 12.34 (2.60) | **< 0.001** |
| PC_aa_C32_1 | 24.87 (11.37) | 18.94 (7.43) | 16.97 (8.55) | 20.09 (9.85) | **< 0.001** |
| PC_aa_C32_2 | 3.96 (1.48) | 4.05 (1.74) | 2.90 (1.13) | 3.58 (1.54) | **< 0.001** |
| PC_aa_C32_3 | 0.42 (0.09) | 0.47 (0.11) | 0.33 (0.07) | 0.40 (0.11) | **< 0.001** |
| PC_aa_C34_1 | 233.51 (52.34) | 201.88 (43.67) | 180.56 (39.26) | 203.90 (50.17) | **< 0.001** |
| PC_aa_C34_2 | 384.89 (62.19) | 374.81 (65.49) | 316.82 (55.48) | 355.83 (68.06) | **< 0.001** |
| PC_aa_C34_3 | 17.21 (4.04) | 16.60 (4.55) | 13.09 (3.70) | 15.45 (4.47) | **< 0.001** |
| PC_aa_C34_4 | 2.06 (0.63) | 1.82 (0.61) | 1.49 (0.51) | 1.77 (0.63) | **< 0.001** |
| PC_aa_C36_1 | 48.06 (12.07) | 43.88 (10.25) | 37.29 (9.28) | 42.70 (11.46) | **< 0.001** |
| PC_aa_C36_2 | 219.56 (40.35) | 220.53 (40.09) | 182.11 (38.73) | 205.49 (43.65) | **< 0.001** |
| PC_aa_C36_3 | 134.11 (23.66) | 124.79 (25.09) | 107.59 (21.51) | 121.20 (25.86) | **< 0.001** |
| PC_aa_C36_4 | 215.68 (44.73) | 179.09 (34.81) | 166.41 (42.11) | 186.02 (46.02) | **< 0.001** |
| PC_aa_C36_5 | 32.68 (13.88) | 26.87 (10.71) | 22.09 (9.76) | 26.91 (12.30) | **< 0.001** |
| PC_aa_C36_6 | 0.88 (0.34) | 0.85 (0.33) | 0.61 (0.23) | 0.77 (0.32) | **< 0.001** |
| PC_aa_C38_0 | 2.78 (0.67) | 3.05 (0.81) | 2.26 (0.57) | 2.66 (0.76) | **< 0.001** |
| PC_aa_C38_1 | 1.39 (0.30) | 1.44 (0.30) | 1.13 (0.27) | 1.30 (0.32) | **< 0.001** |
| PC_aa_C38_3 | 47.39 (9.98) | 40.70 (8.22) | 36.91 (9.76) | 41.40 (10.37) | **< 0.001** |
| PC_aa_C38_4 | 115.78 (27.68) | 95.41 (19.52) | 88.89 (24.83) | 99.48 (26.94) | **< 0.001** |
| PC_aa_C38_5 | 59.36 (13.56) | 50.79 (9.72) | 43.88 (10.80) | 50.90 (13.16) | **< 0.001** |
| PC_aa_C38_6 | 77.46 (20.79) | 71.36 (19.63) | 59.27 (15.59) | 68.69 (20.13) | **< 0.001** |
| PC_aa_C40_2 | 0.33 (0.07) | 0.33 (0.08) | 0.26 (0.06) | 0.30 (0.08) | **< 0.001** |
| PC_aa_C40_3 | 0.62 (0.11) | 0.61 (0.14) | 0.48 (0.11) | 0.56 (0.14) | **< 0.001** |
| PC_aa_C40_4 | 3.66 (0.90) | 2.97 (0.57) | 2.74 (0.87) | 3.11 (0.89) | **< 0.001** |
| PC_aa_C40_5 | 11.15 (2.85) | 8.91 (1.76) | 8.23 (2.62) | 9.37 (2.78) | **< 0.001** |
| PC_aa_C40_6 | 25.79 (8.11) | 22.47 (6.46) | 19.29 (5.82) | 22.32 (7.32) | **< 0.001** |
| PC_aa_C42_0 | 0.46 (0.11) | 0.55 (0.14) | 0.37 (0.09) | 0.45 (0.13) | **< 0.001** |
| PC_aa_C42_1 | 0.25 (0.05) | 0.29 (0.07) | 0.20 (0.05) | 0.24 (0.07) | **< 0.001** |
| PC_aa_C42_2 | 0.23 (0.05) | 0.25 (0.05) | 0.19 (0.03) | 0.22 (0.05) | **< 0.001** |
| PC_aa_C42_4 | 0.20 (0.03) | 0.19 (0.03) | 0.15 (0.03) | 0.18 (0.04) | **< 0.001** |
| PC_aa_C42_5 | 0.42 (0.12) | 0.38 (0.10) | 0.32 (0.10) | 0.37 (0.11) | **< 0.001** |
| PC_aa_C42_6 | 0.34 (0.10) | 0.32 (0.09) | 0.26 (0.08) | 0.30 (0.10) | **< 0.001** |
| PC_ae_C30_0 | 0.40 (0.10) | 0.47 (0.11) | 0.32 (0.08) | 0.39 (0.11) | **< 0.001** |
| PC_ae_C32_1 | 2.71 (0.43) | 2.91 (0.54) | 2.11 (0.39) | 2.54 (0.57) | **< 0.001** |
| PC_ae_C32_2 | 0.71 (0.13) | 0.79 (0.15) | 0.56 (0.11) | 0.68 (0.16) | **< 0.001** |
| PC_ae_C34_0 | 1.31 (0.30) | 1.39 (0.30) | 1.00 (0.21) | 1.21 (0.32) | **< 0.001** |
| PC_ae_C34_1 | 8.51 (1.37) | 9.18 (1.70) | 6.81 (1.30) | 8.06 (1.77) | **< 0.001** |
| PC_ae_C34_2 | 10.01 (2.33) | 11.66 (2.39) | 8.28 (2.30) | 9.83 (2.71) | **< 0.001** |
| PC_ae_C34_3 | 6.18 (1.58) | 7.28 (1.89) | 4.89 (1.44) | 6.01 (1.90) | **< 0.001** |
| PC_ae_C36_0 | 1.03 (0.18) | 1.02 (0.17) | 0.81 (0.14) | 0.94 (0.20) | **< 0.001** |
| PC_ae_C36_1 | 6.57 (1.23) | 7.14 (1.36) | 5.22 (1.03) | 6.22 (1.45) | **< 0.001** |
| PC_ae_C36_2 | 11.21 (2.40) | 13.48 (2.59) | 9.43 (2.31) | 11.20 (2.93) | **< 0.001** |
| PC_ae_C36_3 | 6.17 (1.30) | 6.85 (1.38) | 5.12 (1.35) | 5.97 (1.52) | **< 0.001** |
| PC_ae_C36_4 | 16.94 (4.40) | 15.67 (3.55) | 13.61 (4.01) | 15.29 (4.25) | **< 0.001** |
| PC_ae_C36_5 | 12.85 (2.91) | 11.96 (2.87) | 9.61 (2.42) | 11.35 (3.06) | **< 0.001** |
| PC_ae_C38_0 | 2.42 (0.75) | 2.44 (0.80) | 1.78 (0.50) | 2.18 (0.75) | **< 0.001** |
| PC_ae_C38_1 | 0.62 (0.27) | 0.67 (0.29) | 0.52 (0.18) | 0.60 (0.26) | **< 0.001** |
| PC_ae_C38_2 | 1.33 (0.38) | 1.54 (0.45) | 1.11 (0.30) | 1.31 (0.41) | **< 0.001** |
| PC_ae_C38_3 | 3.29 (0.62) | 3.53 (0.63) | 2.62 (0.55) | 3.10 (0.71) | **< 0.001** |
| PC_ae_C38_4 | 11.52 (2.16) | 11.46 (1.91) | 9.13 (1.89) | 10.59 (2.29) | **< 0.001** |
| PC_ae_C38_5 | 17.01 (3.38) | 16.21 (2.91) | 13.62 (3.16) | 15.48 (3.49) | **< 0.001** |
| PC_ae_C38_6 | 7.34 (1.65) | 7.46 (1.67) | 5.72 (1.34) | 6.76 (1.75) | **< 0.001** |
| PC_ae_C40_1 | 1.66 (0.38) | 1.59 (0.38) | 1.27 (0.28) | 1.49 (0.39) | **< 0.001** |
| PC_ae_C40_2 | 1.62 (0.34) | 1.73 (0.30) | 1.25 (0.28) | 1.51 (0.37) | **< 0.001** |
| PC_ae_C40_3 | 0.94 (0.15) | 1.06 (0.15) | 0.76 (0.14) | 0.91 (0.19) | **< 0.001** |
| PC_ae_C40_4 | 2.24 (0.36) | 2.36 (0.38) | 1.77 (0.34) | 2.09 (0.44) | **< 0.001** |
| PC_ae_C40_5 | 2.85 (0.45) | 2.96 (0.45) | 2.22 (0.43) | 2.64 (0.55) | **< 0.001** |
| PC_ae_C40_6 | 3.78 (0.81) | 4.20 (0.93) | 3.00 (0.67) | 3.60 (0.95) | **< 0.001** |
| PC_ae_C42_1 | 0.40 (0.09) | 0.38 (0.07) | 0.32 (0.07) | 0.36 (0.09) | **< 0.001** |
| PC_ae_C42_2 | 0.53 (0.10) | 0.54 (0.10) | 0.40 (0.08) | 0.48 (0.11) | **< 0.001** |
| PC_ae_C42_3 | 0.76 (0.15) | 0.83 (0.17) | 0.60 (0.12) | 0.72 (0.18) | **< 0.001** |
| PC_ae_C42_4 | 0.69 (0.15) | 0.79 (0.19) | 0.55 (0.14) | 0.66 (0.19) | **< 0.001** |
| PC_ae_C42_5 | 1.98 (0.32) | 2.15 (0.37) | 1.67 (0.32) | 1.91 (0.39) | **< 0.001** |
| PC_ae_C44_3 | 0.13 (0.03) | 0.13 (0.03) | 0.10 (0.02) | 0.12 (0.03) | **< 0.001** |
| PC_ae_C44_4 | 0.34 (0.07) | 0.38 (0.08) | 0.28 (0.06) | 0.33 (0.08) | **< 0.001** |
| PC_ae_C44_5 | 1.71 (0.42) | 1.82 (0.44) | 1.36 (0.34) | 1.61 (0.45) | **< 0.001** |
| PC_ae_C44_6 | 1.08 (0.29) | 1.22 (0.31) | 0.84 (0.20) | 1.03 (0.31) | **< 0.001** |
| SM__OH__C14_1 | 6.62 (1.49) | 8.10 (1.74) | 5.46 (1.28) | 6.61 (1.84) | **< 0.001** |
| SM__OH__C16_1 | 3.40 (0.83) | 3.97 (0.85) | 2.84 (0.71) | 3.35 (0.92) | **< 0.001** |
| SM__OH__C22_1 | 12.15 (2.47) | 13.41 (2.48) | 9.68 (2.19) | 11.57 (2.84) | **< 0.001** |
| SM__OH__C22_2 | 10.60 (2.34) | 12.79 (2.37) | 8.63 (1.92) | 10.49 (2.77) | **< 0.001** |
| SM__OH__C24_1 | 1.20 (0.28) | 1.28 (0.25) | 0.94 (0.24) | 1.12 (0.30) | **< 0.001** |
| SM_C16_0 | 114.49 (15.77) | 121.59 (16.86) | 93.95 (17.02) | 108.71 (20.42) | **< 0.001** |
| SM_C16_1 | 18.38 (3.26) | 19.69 (3.45) | 14.99 (2.94) | 17.46 (3.78) | **< 0.001** |
| SM_C18_0 | 24.42 (4.79) | 24.20 (5.09) | 20.06 (4.57) | 22.69 (5.21) | **< 0.001** |
| SM_C18_1 | 11.86 (2.89) | 12.27 (2.91) | 9.85 (2.61) | 11.21 (2.99) | **< 0.001** |
| SM_C20_2 | 0.60 (0.15) | 0.64 (0.15) | 0.49 (0.11) | 0.57 (0.15) | **< 0.001** |
| SM_C24_0 | 19.72 (3.33) | 18.97 (3.28) | 15.35 (3.56) | 17.82 (3.93) | **< 0.001** |
| SM_C24_1 | 56.07 (8.77) | 55.29 (8.41) | 44.90 (9.00) | 51.56 (10.20) | **< 0.001** |
| H1 | 5348.20 (1190.11) | 4677.10 (708.95) | 4942.25 (1245.02) | 4994.98 (1124.04) | **< 0.001** |


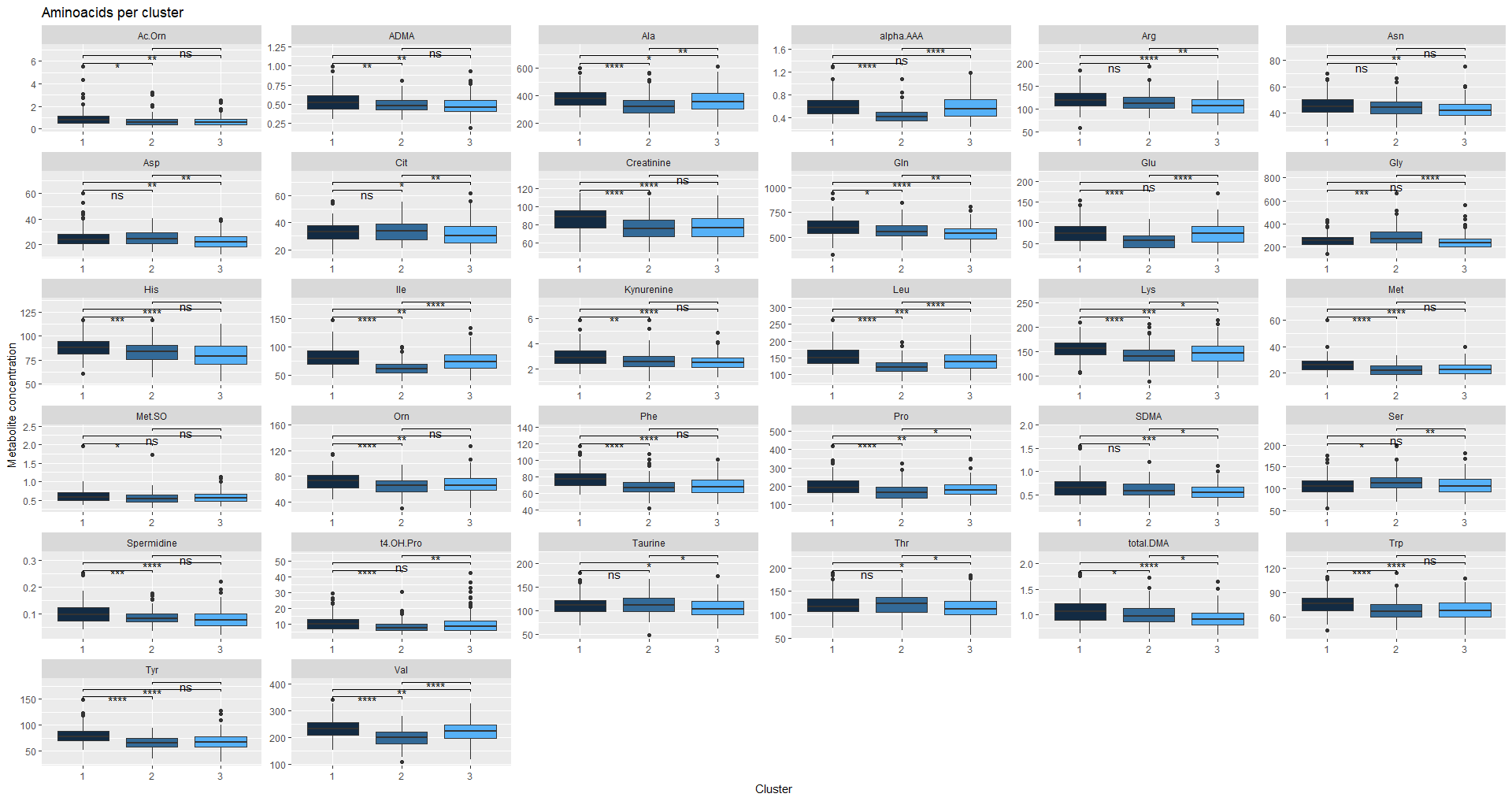

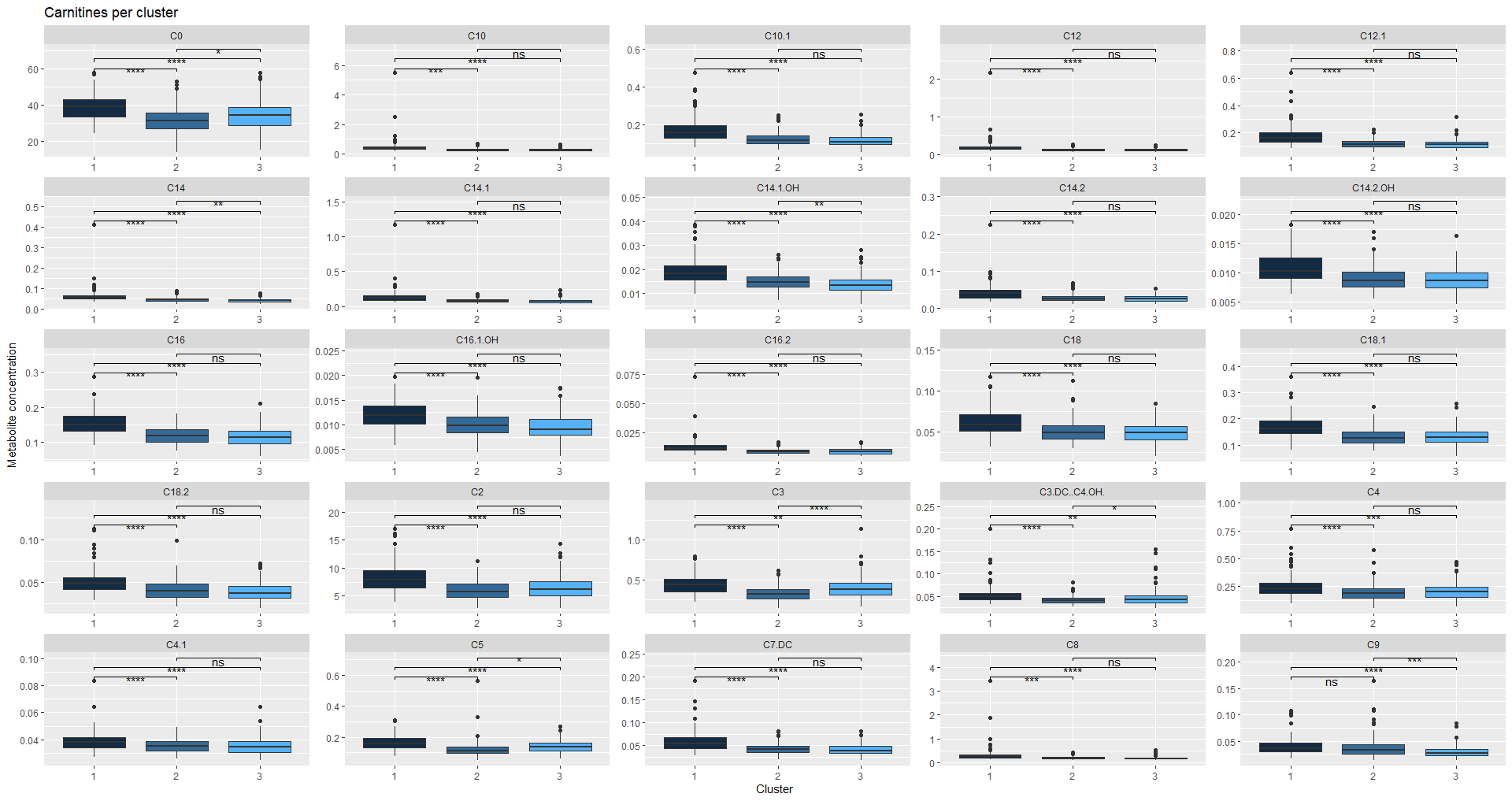


**Supplementary Figure 3B** Boxplots showing the carnitine and acylcarnitine concentrations (µmol/L) in the three clusters of the main exposure (left ventricle parameters) data set. The x axis shows the clusters and the y axis the metabolite concentrations in different scales. Significant differences are indicated as * for a p-value ≤ 0.05, ** for a p-value ≤ 0.01, *** for a p-value ≤ 0.001 and ns for a p-value > 0.05.

**Supplementary Figure 3A** Boxplots showing the amino acids and biogenic amine concentrations (µmol/L) in the three clusters of the main exposure (left ventricle parameters) data set. The x axis shows the clusters and the y axis the metabolite concentrations in different scales. Significant differences are indicated as * for a p-value ≤ 0.05, ** for a p-value ≤ 0.01, *** for a p-value ≤ 0.001 and ns for a p-value > 0.05.

**Supplementary Figures 3A -E** show the concentrations of metabolites (µmol/L) per cluster. The x axis shows the clusters and the y-axis shows the concentration in the scale of the metabolite. 3A) Aminoacids, 3B) carnitine and acylcarnitines 3C) acylalkylphosphatidylcholine, 3D) lysophosphatidylcholine and diacylphosphatidylcholine, 3E) sphingomyelin and hexoses


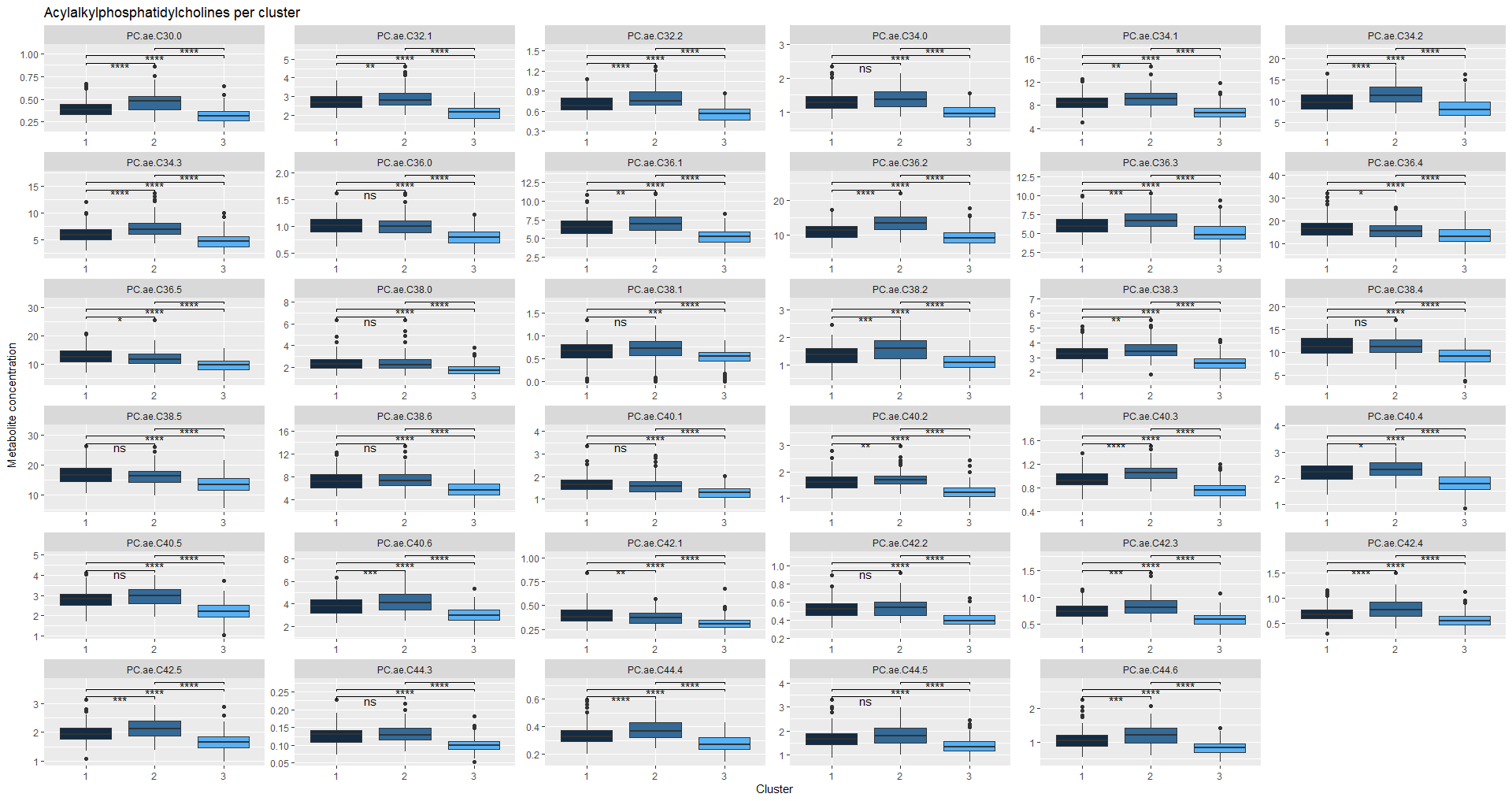

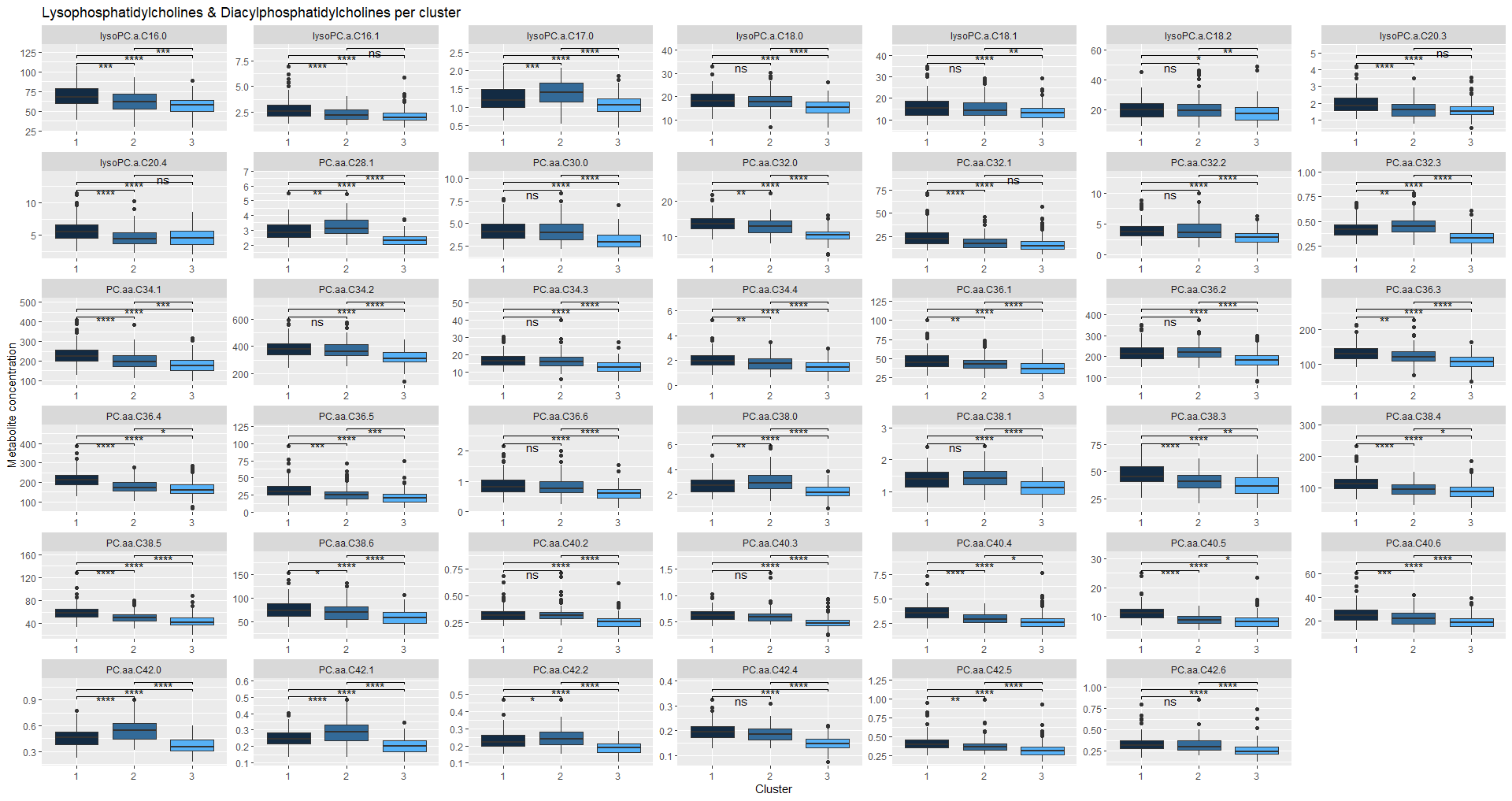


**Supplementary Figure 3C** Boxplots showing the acylalkylphosphatidylcholine concentrations (µmol/L) in the three clusters of the main exposure (left ventricle parameters) data set. The x axis shows the clusters and the y axis the metabolite concentrations in different scales. Significant differences are indicated as * for a p-value ≤ 0.05, ** for a p-value ≤ 0.01, *** for a p-value ≤ 0.001 and ns for a p-value > 0.05.

**Supplementary Figure 3D** Boxplots showing the lysophosphatidylcholine and diacylphosphatidylcholine concentrations (µmol/L) in the three clusters of the main exposure (left ventricle parameters) data set. The x axis shows the clusters and the y axis the metabolite concentrations in different scales. Significant differences are indicated as * for a p-value ≤ 0.05, ** for a p-value ≤ 0.01, *** for a p-value ≤ 0.001 and ns for a p-value > 0.05.


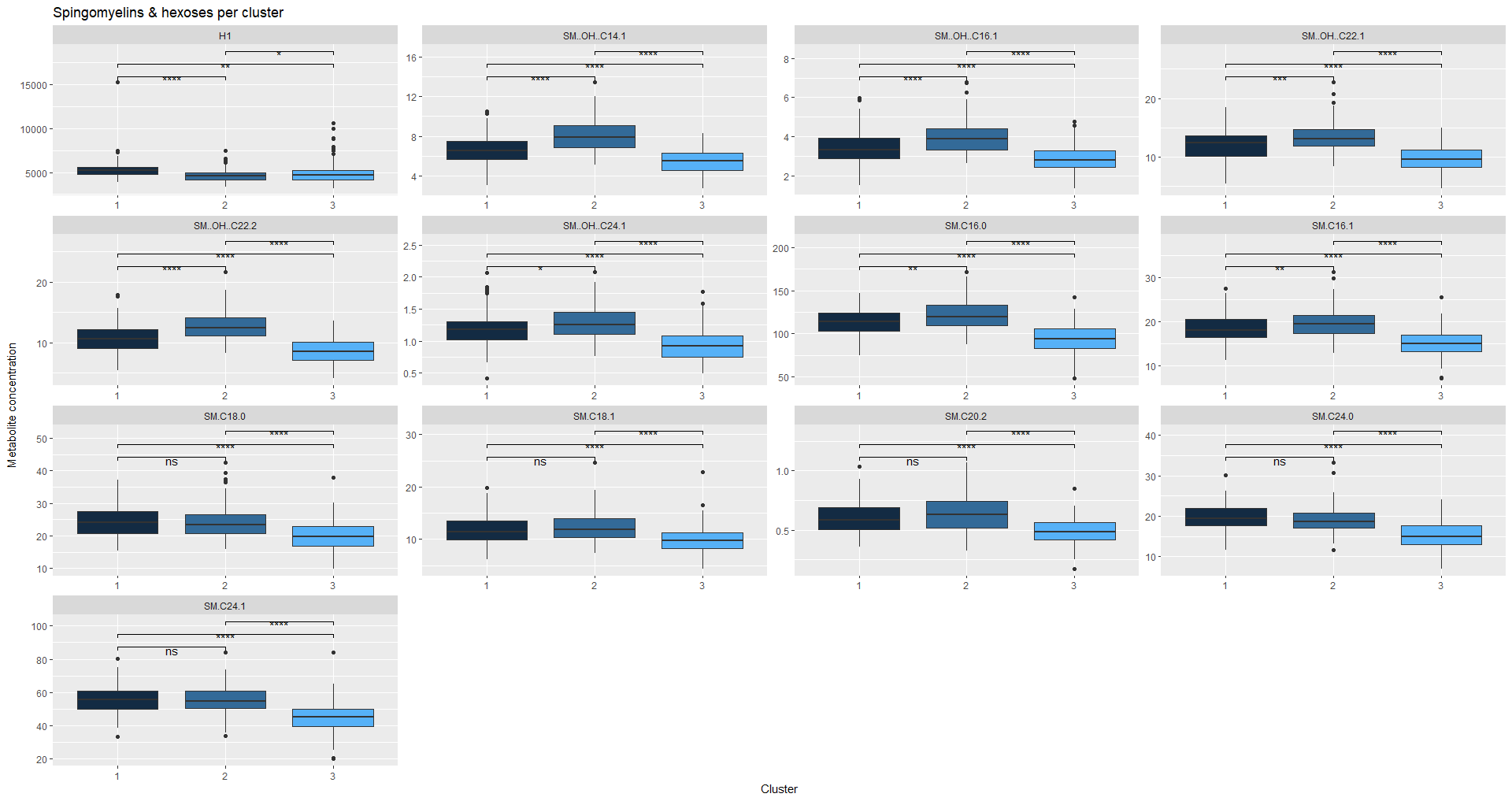


**Supplementary Figure 3E** Boxplots showing the sphingomyelin and hexoses concentrations (µmol/L) in the three clusters of the main exposure (left ventricle parameters) data set. The x axis shows the clusters and the y axis the metabolite concentrations in different scales. Significant differences are indicated as * for a p-value ≤ 0.05, ** for a p-value ≤ 0.01, *** for a p-value ≤ 0.001 and ns for a p-value > 0.05.

**Supplementary Table 8** Pathway analyses in the main exposure data set.

|  | **Hits / total compounds** | **Raw p-value** | **-log10(p)** | **Impact** | **Metabolites included** |
| --- | --- | --- | --- | --- | --- |
| **Pathways in Cluster 1** | | | | | |
| **Aminoacyl-tRNA biosynthesis** | **7/48** | **3.356e-19** | **18.474** | **0** | glutamine, alanine,  asparagine, valine, leucine, isoleucine, proline |
| **Valine, leucine, and isoleucine degradation** | **3/40** | **1.8997e-18** | **17.721** | **0** | valine, leucine, isoleucine |
| **Valine, leucine, and isoleucine biosynthesis** | **3/8** | **1.8997e-18** | **17.721** | **0** | valine, leucine, isoleucine |
| Pantothenate and CoA biosynthesis | 1/19 | 1.7035e-13 | 12.769 | 0 |  |
| **Alanine aspartate and glutamate metabolism** | 2/28 | 3.4841e-09 | 8.4579 | **0.114** | alanine, glutamine |
| Glycolysis / Gluconeogenesis | 1/26 | 6.9848e-09 | 8.1558 | < 0.001 | D-glucose |
| Selenocompound metabolism | 1/20 | 7.7493e-09 | 8.1107 | 0 | alanine |
| Lysine degradation | 1/25 | 1.8816e-07 | 6.7255 | 0 | lysine |
| Biotin metabolism | 1/10 | 1.8816e-07 | 6.7255 | 0 | lysine |
| Arginine and proline metabolism | 1/38 | 2.4927e-07 | 6.6033 | 0.078 | proline |
| **Glycerophospholipid metabolism** | 2/36 | 0.00010745 | 3.9688 | **0.112** | phosphatidylcholine, 2-lysophosphatidylcholine |
| Arachidonic acid metabolism | 1/36 | 0.0013016 | 2.8855 | 0 | phosphatidylcholine |
| Linoleic acid metabolism | 1/5 | 0.0013016 | 2.8855 | 0 | phosphatidylcholine |
| alpha-Linolenic acid metabolism | 1/13 | 0.0013016 | 2.8855 | 0 | phosphatidylcholine |
| Arginine biosynthesis | 1/14 | 0.028752 | 1.5413 | 0 | glutamine |
| Purine metabolism | 1/65 | 0.028752 | 1.5413 | 0 | glutamine |
| Pyrimidine metabolism | 1/39 | 0.028752 | 1.5413 | 0 | glutamine |
| D-Glutamine and D-glutamate metabolism | 1/6 | 0.028752 | 1.5413 | 0 | Glutamine |
| Glyoxylate and dicarboxylate metabolism | 1/32 | 0.028752 | 1.5413 | 0 | Glutamine |
| Nitrogen metabolism | 1/6 | 0.028752 | 1.5413 | 0 | Glutamine |
| **Pathways in Cluster 3** | | | | | |
| **Arachidonic acid metabolism** | **1/36** | **3.5206e-17** | **16.453** | **0** | phosphatidylcholine |
| **Linoleic acid metabolism** | **1/5** | **3.5206e-17** | **16.453** | **0** | phosphatidylcholine |
| **alpha-Linolenic acid metabolism** | **1/13** | **3.5206e-17** | **16.453** | **0** | phosphatidylcholine |
| **Glycerophospholipid metabolism** | **2/36** | **4.4055e-13** | **12.356** | **0.112** | phosphatidylcholine, 2-lysophosphatidylcholine |
| Valine, leucine, and isoleucine degradation | 3/40 | 7.655e-08 | 7.1161 | 0 | valine, leucine, isoleucine |
| Valine, leucine, and isoleucine biosynthesis | 3/8 | 7.655e-08 | 7.1161 | 0 | valine, leucine, isoleucine |
| Aminoacyl-tRNA biosynthesis | 7/48 | 4.9452e-07 | 6.3058 | 0 | glutamine, alanine,  asparagine, valine, leucine, isoleucine, proline |
| Pantothenate and CoA biosynthesis | 1/19 | 1.2342e-05 | 4.9086 | 0 |  |
| **Alanine, aspartate, and glutamate metabolism** | **2/28** | **0.0011785** | **2.9287** | **0.114** | alanine, glutamine |
| Selenocompound metabolism | 1/20 | 0.0048755 | 2.312 | 0 | alanine |
| Arginine biosynthesis | 1/14 | 0.0068491 | 2.1644 | 0 | glutamine |
| Purine metabolism | 1/65 | 0.0068491 | 2.1644 | 0 | glutamine |
| Pyrimidine metabolism | 1/39 | 0.0068491 | 2.1644 | 0 | glutamine |
| D-Glutamine and D-glutamate metabolism | 1/6 | 0.0068491 | 2.1644 | 0 | glutamine |
| Glyoxylate and dicarboxylate metabolism | 1/32 | 0.0068491 | 2.1644 | 0 | glutamine |
| Nitrogen metabolism | 1/6 | 0.0068491 | 2.1644 | 0 | glutamine |
| Arginine and proline metabolism | 1/38 | 0.010065 | 1.9972 | 0.078 | proline |
| Lysine degradation | 1/25 | 0.056445 | 1.2484 | 0 | lysine |
| Biotin metabolism | 1/10 | 0.056445 | 1.2484 | 0 | lysine |
| Glycolysis / Gluconeogenesis | 1/26 | 0.10673 | 0.97171 | < 0.001 | D-glucose |

**Supplementary Table 9** Clinical characteristics, imaging markers, and metabolite concentrations according to metabolite profile clusters in the secondary data set (carotid plaque). Continuous variables are presented as mean (SD) and categorical variables as frequency (proportion). Significance is defined as a p-value ≤ 0.05 and tested using ANOVA or χ2 Test, where applicable.

| **Variable** | **Cluster 1**  **(N = 80)** | **Cluster 2**  **(N = 98)** | **Cluster 3**  **(N = 76)** | **p value** |
| --- | --- | --- | --- | --- |
| Age (years) | 55.8 (9.1) | 54.5 (9.0) | 58.2 (8.9) | **0.026** |
| Female sex | 55 (68.8%) | 29 (29.6%) | 12 (15.8%) | **< 0.001** |
| BMI (kg/m^2^) | 26.1 (4.0) | 28.4 (4.4) | 28.1 (4.2) | **< 0.001** |
| Regularly physically active | 52 (65.0%) | 51 (52.0%) | 49 (64.5%) | 0.132 |
| Smoking status |  |  |  | 0.626 |
| Currently smoking | 18 (22.5%) | 19 (19.4%) | 16 (21.1%) |  |
| Ex - Smoker | 29 (36.2%) | 47 (48.0%) | 31 (40.8%) |  |
| Never - Smoker | 33 (41.2%) | 32 (32.7%) | 29 (38.2%) |  |
| Alcohol consumption (g/Day) | 10.9 (12.9) | 15.9 (22.4) | 26.9 (26.6) | **< 0.001** |
| Diabetes status (based on OGTT) |  |  |  | **0.005** |
| Normoglycemic | 62 (77.5%) | 62 (63.3%) | 41 (53.9%) |  |
| Prediabetes | 11 (13.8%) | 16 (16.3%) | 24 (31.6%) |  |
| Diabetes | 7 (8.8%) | 20 (20.4%) | 11 (14.5%) |  |
| Fasting glucose (mg/dl) | 98.2 (14.7) | 110.2 (30.7) | 106.8 (26.6) | **0.007** |
| Hypertension | 16 (20.0%) | 36 (36.7%) | 28 (36.8%) | **0.028** |
| Systolic blood pressure (mmHg) | 115.1 (15.5) | 120.9 (17.7) | 124.9 (16.1) | **0.001** |
| Diastolic blood pressure (mmHg) | 73.0 (9.3) | 75.6 (11.2) | 76.9 (8.7) | **0.042** |
| Angina pectoris | 7 (8.8%) | n < 5 | 8 (10.5%) | 0.242 |
| Total cholesterol (mg/dl) | 236.0 (35.4) | 197.2 (29.2) | 223.8 (32.7) | **< 0.001** |
| HDL cholesterol (mg/dl) | 71.5 (18.9) | 52.9 (12.6) | 60.2 (15.7) | **< 0.001** |
| LDL cholesterol (mg/dl) | 150.9 (35.2) | 127.7 (28.7) | 144.1 (31.6) | **< 0.001** |
| Triglycerides (mg/dl) | 108.5 (64.2) | 135.2 (77.1) | 163.9 (120.9) | **< 0.001** |
| Antidiabetic drugs | 5 (6.2%) | 16 (16.3%) | 5 (6.6%) | **0.040** |
| Antihypertensive drugs | 14 (17.5%) | 27 (27.6%) | 20 (26.3%) | 0.252 |
| Lipid lowering drug | n < 5 | 18 (18.4%) | 7 (9.2%) | **0.016** |
| Anticoagulant drugs | n < 5 | n < 5 | n < 5 | 0.688 |
| Antiplatelet drugs | n < 5 | 5 (5.1%) | n < 5 | 0.117 |
| hsCRP | 0.1 (0.39;2.34) | 1.14 (0.67;2.34) | 1.09 (0.60;2.36) | 0.395 |
| SCORE2 | 4.1 (3.4) | 5.0 (3.3) | 6.6 (4.0) | **< 0.001** |
| **MRI variables** |  |  |  |  |
| Any plaque present | 17 (21.2%) | 18 (18.4%) | 19 (25.0%) | 0.570 |
| Plaque index |  |  |  | 0.719 |
| Normal – diffuse thickness | 63 (78.8%) | 80 (81.6%) | 57 (75.0%) |  |
| Plaque – complex plaque | 12 (15.0%) | 14 (14.3%) | 12 (15.8%) |  |
| Fibrotic plaque | 5 (6.2%) | n < 5 | 7 (9.2%) |  |
| Normalized wall index | 0.4 (0.0) | 0.4 (0.0) | 0.4 (0.0) | 0.496 |
|  |  |  |  |  |
| **Metabolites (µmol/L)** |  |  |  |  |
| Ala | 341.78 (82.77) | 369.60 (83.06) | 383.01 (73.86) | **0.005** |
| Arg | 114.10 (21.31) | 107.18 (18.50) | 118.43 (20.05) | **< 0.001** |
| Asn | 44.94 (7.93) | 43.21 (7.02) | 45.45 (8.29) | 0.128 |
| Asp | 24.16 (5.63) | 22.57 (5.43) | 23.80 (6.38) | 0.156 |
| Cit | 33.40 (8.45) | 32.47 (8.98) | 32.70 (7.53) | 0.754 |
| Gln | 580.82 (87.47) | 549.24 (87.62) | 597.63 (97.80) | **0.002** |
| Glu | 60.37 (23.74) | 74.44 (28.23) | 70.67 (24.51) | **0.001** |
| Gly | 297.45 (86.08) | 248.33 (68.83) | 258.79 (56.99) | **< 0.001** |
| His | 84.75 (11.11) | 81.85 (12.38) | 89.21 (10.79) | **< 0.001** |
| Ile | 62.87 (12.96) | 77.05 (17.62) | 82.96 (18.14) | **< 0.001** |
| Leu | 123.81 (21.81) | 144.25 (27.45) | 155.94 (28.19) | **< 0.001** |
| Lys | 145.25 (19.66) | 150.05 (20.12) | 157.31 (18.01) | **< 0.001** |
| Met | 22.88 (4.58) | 23.64 (4.81) | 25.91 (4.54) | **< 0.001** |
| Orn | 65.39 (12.17) | 68.26 (13.90) | 72.38 (13.52) | **0.005** |
| Phe | 68.13 (10.61) | 68.94 (10.09) | 77.16 (10.77) | **< 0.001** |
| Pro | 173.83 (44.42) | 189.73 (44.06) | 202.27 (53.14) | **< 0.001** |
| Ser | 115.72 (21.58) | 106.61 (21.14) | 105.89 (22.60) | **0.006** |
| Thr | 124.71 (24.52) | 118.14 (22.93) | 118.84 (20.03) | 0.123 |
| Trp | 68.29 (12.70) | 69.17 (13.37) | 75.10 (12.68) | **0.002** |
| Tyr | 66.63 (13.48) | 68.79 (16.27) | 79.33 (14.56) | **< 0.001** |
| Val | 199.26 (34.20) | 225.38 (38.17) | 236.46 (36.39) | **< 0.001** |
| Ac.Orn | 0.81 (0.81) | 0.65 (0.37) | 1.06 (0.85) | **< 0.001** |
| ADMA | 0.49 (0.12) | 0.49 (0.12) | 0.53 (0.13) | **0.029** |
| alpha.AAA | 0.46 (0.15) | 0.61 (0.22) | 0.61 (0.18) | **< 0.001** |
| Creatinine | 76.34 (12.57) | 80.93 (14.73) | 87.70 (13.90) | **< 0.001** |
| Kynurenine | 2.58 (0.56) | 2.65 (0.73) | 2.95 (0.78) | **0.002** |
| Met.SO | 0.54 (0.12) | 0.58 (0.17) | 0.58 (0.14) | 0.069 |
| SDMA | 0.63 (0.18) | 0.60 (0.17) | 0.67 (0.23) | 0.052 |
| Spermidine | 0.09 (0.03) | 0.08 (0.03) | 0.11 (0.04) | **< 0.001** |
| t4.OH.Pro | 8.53 (3.97) | 10.91 (6.47) | 11.60 (4.96) | **< 0.001** |
| Taurine | 113.03 (20.42) | 106.21 (21.12) | 112.39 (20.71) | 0.053 |
| total.DMA | 1.01 (0.20) | 0.97 (0.18) | 1.06 (0.22) | **0.026** |
| C0 | 32.99 (7.71) | 34.29 (7.40) | 39.60 (7.90) | **< 0.001** |
| C2 | 6.32 (1.87) | 6.52 (2.01) | 8.66 (2.57) | **< 0.001** |
| C3 | 0.36 (0.12) | 0.41 (0.15) | 0.43 (0.12) | **0.001** |
| C3_DC__C4_OH_ | 0.04 (0.02) | 0.05 (0.02) | 0.06 (0.02) | **< 0.001** |
| C4 | 0.20 (0.08) | 0.21 (0.08) | 0.25 (0.11) | **< 0.001** |
| C4_1 | 0.04 (0.01) | 0.04 (0.01) | 0.04 (0.01) | **0.042** |
| C5 | 0.12 (0.06) | 0.15 (0.04) | 0.16 (0.04) | **< 0.001** |
| C7_DC | 0.04 (0.01) | 0.04 (0.01) | 0.06 (0.02) | **< 0.001** |
| C8 | 0.20 (0.09) | 0.20 (0.11) | 0.30 (0.21) | **< 0.001** |
| C9 | 0.04 (0.02) | 0.03 (0.01) | 0.04 (0.02) | **< 0.001** |
| C10 | 0.30 (0.14) | 0.29 (0.13) | 0.45 (0.28) | **< 0.001** |
| C10_1 | 0.12 (0.04) | 0.12 (0.04) | 0.17 (0.05) | **< 0.001** |
| C12 | 0.13 (0.04) | 0.12 (0.04) | 0.20 (0.09) | **< 0.001** |
| C12_1 | 0.12 (0.03) | 0.12 (0.04) | 0.18 (0.07) | **< 0.001** |
| C14 | 0.04 (0.01) | 0.04 (0.01) | 0.06 (0.02) | **< 0.001** |
| C14_1 | 0.09 (0.03) | 0.08 (0.03) | 0.14 (0.06) | **< 0.001** |
| C14_1_OH | 0.01 (0.00) | 0.01 (0.00) | 0.02 (0.01) | **< 0.001** |
| C14_2 | 0.03 (0.01) | 0.03 (0.01) | 0.04 (0.02) | **< 0.001** |
| C14_2_OH | 0.01 (0.00) | 0.01 (0.00) | 0.01 (0.00) | **< 0.001** |
| C16 | 0.12 (0.03) | 0.12 (0.03) | 0.16 (0.03) | **< 0.001** |
| C16_1_OH | 0.01 (0.00) | 0.01 (0.00) | 0.01 (0.00) | **< 0.001** |
| C16_2 | 0.01 (0.00) | 0.01 (0.00) | 0.01 (0.00) | **< 0.001** |
| C18 | 0.05 (0.01) | 0.05 (0.01) | 0.06 (0.02) | **< 0.001** |
| C18_1 | 0.14 (0.03) | 0.13 (0.03) | 0.17 (0.04) | **< 0.001** |
| C18_2 | 0.04 (0.01) | 0.04 (0.01) | 0.05 (0.01) | **< 0.001** |
| lysoPC_a_C16_0 | 64.22 (12.63) | 57.40 (10.58) | 68.19 (11.48) | **< 0.001** |
| lysoPC_a_C16_1 | 2.31 (0.61) | 2.09 (0.65) | 2.90 (1.06) | **< 0.001** |
| lysoPC_a_C17_0 | 1.43 (0.35) | 1.07 (0.27) | 1.23 (0.32) | **< 0.001** |
| lysoPC_a_C18_0 | 18.66 (4.35) | 15.60 (3.38) | 18.18 (3.71) | **< 0.001** |
| lysoPC_a_C18_1 | 15.47 (4.35) | 13.66 (4.04) | 16.92 (5.92) | **< 0.001** |
| lysoPC_a_C18_2 | 20.55 (7.02) | 19.04 (7.20) | 20.84 (6.94) | 0.188 |
| lysoPC_a_C20_3 | 1.67 (0.48) | 1.64 (0.49) | 2.02 (0.62) | **< 0.001** |
| lysoPC_a_C20_4 | 4.96 (1.47) | 4.76 (1.48) | 5.91 (1.97) | **< 0.001** |
| PC_aa_C28_1 | 3.30 (0.68) | 2.37 (0.45) | 2.89 (0.66) | **< 0.001** |
| PC_aa_C30_0 | 4.22 (1.32) | 3.13 (0.88) | 4.29 (1.13) | **< 0.001** |
| PC_aa_C32_0 | 13.47 (2.60) | 10.70 (1.80) | 13.97 (2.24) | **< 0.001** |
| PC_aa_C32_1 | 19.30 (7.81) | 16.50 (7.94) | 25.56 (10.27) | **< 0.001** |
| PC_aa_C32_2 | 4.09 (1.76) | 3.03 (1.18) | 4.15 (1.43) | **< 0.001** |
| PC_aa_C32_3 | 0.47 (0.11) | 0.34 (0.07) | 0.42 (0.09) | **< 0.001** |
| PC_aa_C34_1 | 211.78 (46.93) | 183.11 (35.59) | 238.81 (52.34) | **< 0.001** |
| PC_aa_C34_2 | 381.14 (66.91) | 327.11 (52.66) | 385.77 (64.40) | **< 0.001** |
| PC_aa_C34_3 | 16.75 (4.82) | 13.37 (3.71) | 17.58 (4.09) | **< 0.001** |
| PC_aa_C34_4 | 1.93 (0.65) | 1.53 (0.48) | 2.15 (0.68) | **< 0.001** |
| PC_aa_C36_1 | 45.34 (10.66) | 37.55 (8.44) | 48.82 (12.48) | **< 0.001** |
| PC_aa_C36_2 | 223.54 (41.79) | 186.84 (38.18) | 219.65 (40.09) | **< 0.001** |
| PC_aa_C36_3 | 128.32 (26.72) | 109.80 (19.04) | 134.68 (24.55) | **< 0.001** |
| PC_aa_C36_4 | 193.03 (38.61) | 170.40 (39.04) | 220.37 (47.45) | **< 0.001** |
| PC_aa_C36_5 | 30.99 (13.99) | 22.13 (10.13) | 33.22 (14.08) | **< 0.001** |
| PC_aa_C36_6 | 0.93 (0.39) | 0.62 (0.22) | 0.89 (0.31) | **< 0.001** |
| PC_aa_C38_0 | 3.21 (0.78) | 2.30 (0.57) | 2.72 (0.59) | **< 0.001** |
| PC_aa_C38_1 | 1.52 (0.32) | 1.16 (0.27) | 1.35 (0.29) | **< 0.001** |
| PC_aa_C38_3 | 42.68 (8.99) | 37.38 (9.34) | 46.59 (10.34) | **< 0.001** |
| PC_aa_C38_4 | 103.26 (20.65) | 90.48 (22.90) | 116.88 (29.44) | **< 0.001** |
| PC_aa_C38_5 | 55.34 (10.40) | 44.40 (9.34) | 60.84 (14.68) | **< 0.001** |
| PC_aa_C38_6 | 78.21 (20.09) | 60.96 (14.79) | 78.38 (19.37) | **< 0.001** |
| PC_aa_C40_2 | 0.35 (0.09) | 0.26 (0.07) | 0.32 (0.06) | **< 0.001** |
| PC_aa_C40_3 | 0.64 (0.16) | 0.49 (0.11) | 0.61 (0.11) | **< 0.001** |
| PC_aa_C40_4 | 3.14 (0.64) | 2.76 (0.70) | 3.71 (0.97) | **< 0.001** |
| PC_aa_C40_5 | 9.55 (1.81) | 8.27 (2.06) | 11.41 (3.09) | **< 0.001** |
| PC_aa_C40_6 | 24.39 (6.37) | 19.64 (5.42) | 26.02 (8.20) | **< 0.001** |
| PC_aa_C42_0 | 0.56 (0.14) | 0.38 (0.09) | 0.45 (0.11) | **< 0.001** |
| PC_aa_C42_1 | 0.29 (0.06) | 0.21 (0.05) | 0.25 (0.05) | **< 0.001** |
| PC_aa_C42_2 | 0.25 (0.06) | 0.19 (0.03) | 0.22 (0.04) | **< 0.001** |
| PC_aa_C42_4 | 0.20 (0.03) | 0.15 (0.03) | 0.20 (0.03) | **< 0.001** |
| PC_aa_C42_5 | 0.41 (0.11) | 0.32 (0.09) | 0.42 (0.12) | **< 0.001** |
| PC_aa_C42_6 | 0.35 (0.10) | 0.26 (0.07) | 0.34 (0.10) | **< 0.001** |
| PC_ae_C30_0 | 0.46 (0.11) | 0.33 (0.08) | 0.40 (0.10) | **< 0.001** |
| PC_ae_C32_1 | 2.92 (0.53) | 2.14 (0.40) | 2.68 (0.42) | **< 0.001** |
| PC_ae_C32_2 | 0.80 (0.14) | 0.56 (0.12) | 0.69 (0.11) | **< 0.001** |
| PC_ae_C34_0 | 1.42 (0.31) | 1.00 (0.22) | 1.31 (0.32) | **< 0.001** |
| PC_ae_C34_1 | 9.31 (1.73) | 6.86 (1.23) | 8.46 (1.39) | **< 0.001** |
| PC_ae_C34_2 | 11.69 (2.30) | 8.48 (2.30) | 9.85 (2.44) | **< 0.001** |
| PC_ae_C34_3 | 7.24 (1.89) | 5.03 (1.56) | 6.00 (1.59) | **< 0.001** |
| PC_ae_C36_0 | 1.06 (0.19) | 0.82 (0.15) | 1.03 (0.18) | **< 0.001** |
| PC_ae_C36_1 | 7.31 (1.44) | 5.29 (1.05) | 6.52 (1.29) | **< 0.001** |
| PC_ae_C36_2 | 13.49 (2.63) | 9.70 (2.41) | 11.17 (2.55) | **< 0.001** |
| PC_ae_C36_3 | 6.90 (1.41) | 5.23 (1.33) | 6.08 (1.32) | **< 0.001** |
| PC_ae_C36_4 | 16.62 (4.23) | 13.84 (3.70) | 16.76 (4.26) | **< 0.001** |
| PC_ae_C36_5 | 12.85 (3.28) | 9.83 (2.35) | 12.68 (2.50) | **< 0.001** |
| PC_ae_C38_0 | 2.66 (0.97) | 1.82 (0.49) | 2.44 (0.66) | **< 0.001** |
| PC_ae_C38_1 | 0.72 (0.24) | 0.54 (0.17) | 0.67 (0.21) | **< 0.001** |
| PC_ae_C38_2 | 1.50 (0.45) | 1.12 (0.31) | 1.30 (0.40) | **< 0.001** |
| PC_ae_C38_3 | 3.58 (0.67) | 2.64 (0.54) | 3.19 (0.59) | **< 0.001** |
| PC_ae_C38_4 | 11.97 (2.00) | 9.32 (1.76) | 11.48 (2.10) | **< 0.001** |
| PC_ae_C38_5 | 17.16 (3.19) | 13.85 (2.76) | 16.81 (3.20) | **< 0.001** |
| PC_ae_C38_6 | 7.99 (1.76) | 5.83 (1.33) | 7.23 (1.37) | **< 0.001** |
| PC_ae_C40_1 | 1.68 (0.41) | 1.31 (0.25) | 1.71 (0.39) | **< 0.001** |
| PC_ae_C40_2 | 1.78 (0.33) | 1.27 (0.29) | 1.58 (0.35) | **< 0.001** |
| PC_ae_C40_3 | 1.06 (0.15) | 0.76 (0.14) | 0.92 (0.13) | **< 0.001** |
| PC_ae_C40_4 | 2.41 (0.35) | 1.81 (0.33) | 2.22 (0.35) | **< 0.001** |
| PC_ae_C40_5 | 3.07 (0.45) | 2.25 (0.39) | 2.80 (0.42) | **< 0.001** |
| PC_ae_C40_6 | 4.44 (0.93) | 3.07 (0.64) | 3.73 (0.77) | **< 0.001** |
| PC_ae_C42_1 | 0.39 (0.07) | 0.32 (0.06) | 0.41 (0.09) | **< 0.001** |
| PC_ae_C42_2 | 0.55 (0.10) | 0.41 (0.07) | 0.53 (0.10) | **< 0.001** |
| PC_ae_C42_3 | 0.84 (0.18) | 0.61 (0.13) | 0.76 (0.16) | **< 0.001** |
| PC_ae_C42_4 | 0.79 (0.19) | 0.57 (0.15) | 0.68 (0.16) | **< 0.001** |
| PC_ae_C42_5 | 2.18 (0.35) | 1.70 (0.31) | 1.95 (0.32) | **< 0.001** |
| PC_ae_C44_3 | 0.13 (0.02) | 0.10 (0.02) | 0.13 (0.03) | **< 0.001** |
| PC_ae_C44_4 | 0.37 (0.08) | 0.29 (0.06) | 0.34 (0.07) | **< 0.001** |
| PC_ae_C44_5 | 1.85 (0.44) | 1.39 (0.34) | 1.70 (0.41) | **< 0.001** |
| PC_ae_C44_6 | 1.23 (0.30) | 0.86 (0.21) | 1.07 (0.30) | **< 0.001** |
| SM__OH__C14_1 | 8.14 (1.81) | 5.57 (1.33) | 6.38 (1.65) | **< 0.001** |
| SM__OH__C16_1 | 4.03 (0.89) | 2.91 (0.76) | 3.27 (0.86) | **< 0.001** |
| SM__OH__C22_1 | 13.62 (2.35) | 10.03 (2.20) | 11.75 (2.56) | **< 0.001** |
| SM__OH__C22_2 | 13.07 (2.40) | 8.82 (1.98) | 10.17 (2.28) | **< 0.001** |
| SM__OH__C24_1 | 1.30 (0.25) | 0.97 (0.26) | 1.16 (0.30) | **< 0.001** |
| SM_C16_0 | 123.58 (17.15) | 96.82 (16.49) | 110.35 (16.55) | **< 0.001** |
| SM_C16_1 | 19.96 (3.33) | 15.17 (2.64) | 17.75 (3.30) | **< 0.001** |
| SM_C18_0 | 24.84 (4.94) | 20.66 (4.37) | 23.71 (4.68) | **< 0.001** |
| SM_C18_1 | 12.52 (2.72) | 9.95 (2.33) | 11.47 (2.77) | **< 0.001** |
| SM_C20_2 | 0.67 (0.15) | 0.49 (0.10) | 0.60 (0.15) | **< 0.001** |
| SM_C24_0 | 19.37 (3.32) | 15.96 (3.48) | 19.15 (3.30) | **< 0.001** |
| SM_C24_1 | 57.75 (8.77) | 46.46 (7.95) | 53.91 (8.53) | **< 0.001** |
| H1 | 4798.54 (880.90) | 5148.81 (1375.80) | 5354.22 (1376.51) | **0.019** |

**Supplementary Table 10** Jaccard clusters indices for the clusters for the k-means clusters of the main exposure and secondary exposure data set, as well as for the hierarchical clustering of the main exposure data set.

| **Clusters** | **Jaccard index** |
| --- | --- |
| K-Means: Main exposure | |
| 1 | 0.679 |
| 2 | 0.691 |
| 3 | 0.864 |
| K-Means: Secondary exposure | |
| 1 | 0.660 |
| 2 | 0.816 |
| 3 | 0.695 |
| Hierarchical clustering: main exposure | |
| 1 | 0.497 |
| 2 | 0.521 |
| 3 | 0.716 |

**Supplementary Table 11** Comparison of k-means clusters with agglomerative hierarchical clusters; Ward algorithm. The intersection of individuals in the same clusters are shown as absolute numbers. The percentages refer to the proportion of individuals from the k-means clustering who were also assigned to a group by the hierarchical clustering (intersection / N of k-means clusters).

|  | **K-means Clustering** | | | |
| --- | --- | --- | --- | --- |
| **Agglomerative Hierarchical** | Cluster 1 | Cluster 2 | Cluster 3 | Total |
| Cluster 1 | 31 (26.7%) | 30 (49.1%) | 0 (0%) | 61 |
| Cluster 2 | **71 (61.2%)** | **69 (65.1%)** | 6 (4.3%) | 146 |
| Cluster 3 | 14 (12.1%) | 7 (6.6%) | **132 (95.7%)** | 153 |
| Total | 116 | 106 | 138 | 360 |

**Supplementary Table 12** Results of the multinomial logistic regression additionally adjusted for hsCRP for the main data set. Cluster 2 was used as reference. A p-value ≤0.05 is considered as significant. RR = Relative risk; CI = Confidence interval

|  | **Cluster** | **RR ratios** | **95% CI** | **P-value** |
| --- | --- | --- | --- | --- |
| **MRI marker** |  |  |  |  |
| End-diastolic volume in LV (ml) | **1** | **0.569** | **0.399, 0.812** | **0.0019** |
|  | **3** | **0.635** | **0.453, 0.891** | **0.0086** |
| End-systolic volume in LV (ml) | 1 | 0.745 | 0.527, 1.053 | 0.0952 |
|  | 3 | 0.923 | 0.68, 1.263 | 0.6309 |
| Stroke volume (ml) | **1** | **0.537** | **0.373, 0.771** | **0.0008** |
|  | **3** | **0.509** | **0.354, 0.73** | **0.0002** |
| Cardiac output of LV (ml/min) | **1** | **0.750** | **0.568, 0.991** | **0.0427** |
|  | **3** | **0.643** | **0.49, 0.845** | **0.0015** |
| Ejection fraction of LV (%) | 1 | 0.996 | 0.714, 1.389 | 0.98 |
|  | 3 | 0.803 | 0.576, 1.119 | 0.1952 |
| Peak ejection rate (ml/s) | 1 | 0.748 | 0.554, 1.01 | 0.0583 |
|  | 3 | 0.742 | 0.546, 1.007 | 0.0557 |
| Cardiac mass, diastolic (g) | 1 | 0.875 | 0.607, 1.263 | 0.477 |
|  | 3 | 0.863 | 0.593, 1.257 | 0.443 |
| Cardiac mass, systolic (g) | 1 | 0.941 | 0.63, 1.405 | 0.7668 |
|  | 3 | 0.990 | 0.655, 1.494 | 0.9604 |
| Late Gadolinium Enhancement | 1 | 0.768 | 0.176, 3.356 | 0.7261 |
|  | 3 | 1.428 | 0.32, 6.375 | 0.6409 |
| Early diastolic filling rate (ml/s) | **1** | **0.519** | **0.375, 0.719** | **0.0001** |
|  | **3** | **0.590** | **0.434, 0.802** | **0.0008** |
| Late diastolic filling rate (ml/s) | 1 | 1.009 | 0.737, 1.382 | 0.9559 |
|  | 3 | 0.927 | 0.674, 1.274 | 0.6391 |
| All segments (mm/m^2^) | 1 | 0.988 | 0.698, 1.4 | 0.9472 |
|  | 3 | 1.021 | 0.714, 1.461 | 0.9076 |
| Basal segments (mm/m^2^) | 1 | 1.017 | 0.73, 1.417 | 0.9201 |
|  | 3 | 0.996 | 0.705, 1.407 | 0.9818 |
| Mid segments (mm/m^2^) | 1 | 1.109 | 0.778, 1.582 | 0.5667 |
|  | 3 | 1.144 | 0.792, 1.653 | 0.473 |
| Apical segments (mm/m^2^) | 1 | 0.821 | 0.595, 1.134 | 0.2319 |
|  | 3 | 0.903 | 0.654, 1.246 | 0.5351 |
| Lateral segments (mm/m^2^) | 1 | 0.902 | 0.65, 1.25 | 0.5346 |
|  | 3 | 0.843 | 0.603, 1.177 | 0.3159 |
| Septal segments (mm/m^2^) | 1 | 1.120 | 0.782, 1.604 | 0.5351 |
|  | 3 | 1.150 | 0.793, 1.666 | 0.4612 |
| Anterior segments (mm/m^2^) | 1 | 0.949 | 0.67, 1.346 | 0.7709 |
|  | 3 | 1.033 | 0.723, 1.476 | 0.8579 |
| Inferior segments (mm/m2) | 1 | 1.029 | 0.734, 1.442 | 0.8689 |
|  | 3 | 1.172 | 0.834, 1.647 | 0.361 |
| **Other markers** |  |  |  |  |
| SCORE2 | 1 | 2.158 | 1.121, 4.154 | 0.0213 |
|  | 3 | 3.424 | 1.616, 7.257 | 0.0013 |
| Hypertension | 1 | 2.064 | 0.974, 4,377 | 0.05867 |
|  | **3** | **2.619** | **1.218, 5,631** | **0.0137** |
| Angina Pectoris | 1 | 0.765 | 0.237, 2.466 | 0.654 |
|  | 3 | 0.278 | 0.077, 1.011 | 0.0519 |

**Supplementary Table 13** Results of the multinomial logistic regression for the secondary exposure. All models were adjusted for age, sex, diabetes, hypertension, total cholesterol, and smoking status. Reference = Cluster 1. RR = relative risk; CI = confidence interval

| **MRI variable** | **Cluster** | **RR ratio** | **95% CI** | **P value** |
| --- | --- | --- | --- | --- |
| Plaque normalized wall index | 2 | 4.89 | 0.378; 63.257 | 0.22 |
|  | 3 | 8.88 | 0.547; 144.23 | 0.12 |
| Plaque index 3 | 2 | 1.12 | 0.39; 3.16 | 0.84 |
|  | 3 | 0.90 | 0.30; 2.71 | 0.85 |
| Plaque index 5 | 2 | 0.28 | 0.05;1.66 | 0.16 |
|  | 3 | 0.69 | 0.17;2.84 | 0.61 |
| Any plaque present | 2 | 0.81 | 0.32; 2.07 | 0.66 |
|  | 3 | 0.87 | 0.34; 2.21 | 0.76 |

*Results of multivariable linear regression*

**Supplementary Table 14** Significant results of multivariable linear regression for the main data set. Adjustment for age, sex, diabetes status, systolic blood pressure, and smoking status. Significance was defined as a Bonferroni corrected p-value ≤ 0.05

| **Exposure** | **Metabolite** | **Sub pathway** | **Effect estimate** | **95% CI** | **P value** |
| --- | --- | --- | --- | --- | --- |
| **MRI-derived** |  |  |  |  |  |
| Stroke volume | PC.aa.C42.2 | Phosphatidylcholine metabolism | 0.024 | 0.013; 0.034 | 0.002 |
| Stroke volume | PC.ae.C34.3 | Phosphatidylcholine metabolism | 0.021 | 0.011; 0.031 | 0.012 |
| Stroke volume | C5 | Fatty acid metabolism | -0.025 | -0.035; -0.014 | < 0.001 |
| Stroke volume | PC.ae.C40.6 | Phosphatidylcholine metabolism | 0.021 | 0.011; 0.031 | 0.008 |
| Stroke volume | PC.aa.C38.0 | Phosphatidylcholine metabolism | 0.02 | 0.01; 0.03 | 0.024 |
| Early diastolic filling rate | C5 | Fatty acid metabolism | -0.002 | -0.003; -0.001 | 0.005 |
| Early diastolic filling rate | lysoPC.a.C17.0 | Phosphatidylcholine metabolism | 0.002 | 0.001; 0.003 | 0.016 |
| Early diastolic filling rate | PC.aa.C42.2 | Phosphatidylcholine metabolism | 0.002 | 0.001; 0.003 | 0.005 |
| End diastolic volume | lysoPC.a.C17.0 | Phosphatidylcholine metabolism | 0.014 | 0.007; 0.021 | 0.009 |
| End diastolic volume | lysoPC.a.C18.1 | Phosphatidylcholine metabolism | 0.013 | 0.006; 0.02 | 0.025 |
| Peak ejection rate | lysoPC.a.C17.0 | Phosphatidylcholine metabolism | 0.001 | 0.001; 0.002 | 0.0358 |
| All segments | Ala | Alanine and aspartate  metabolism | -0.362 | -0.519; -0.204 | 0.001 |
| Apical | Ala | Alanine and aspartate  metabolism | -0.28 | -0.412; -0.148 | 0.005 |
| Apical | SDMA | Arginine and proline metabolism | -0.294 | -0.432; -0.156 | 0.005 |
| Inferior | Creatinine | Arginine and proline  metabolism-creatinine pathway | -0.263 | -0.394; -0.133 | 0.013 |
| Inferior | Ala | Alanine and aspartate  metabolism | -0.337 | -0.483; -0.192 | 0.001 |
| Inferior | SDMA | Arginine and proline metabolism | -0.294 | -0.448; -0.14 | 0.030 |
| Lateral | Ala | Alanine and aspartate  metabolism | -0.318 | -0.458; -0.177 | 0.002 |
| Basal | Ala | Alanine and aspartate  metabolism | -0.263 | -0.402; -0.125 | 0.031 |
| Septal | Ala | Alanine and aspartate  metabolism | -0.3 | -0.459; -0.141 | 0.035 |
| **Non-MRI derived** |  |  |  |  |  |
| Hypertension | lysoPC.a.C17.0 | Phosphatidylcholine metabolism | -0.458 | -0,681; -0,235 | 0.010 |
| Hypertension | PC.ae.C42.2 | Phosphatidylcholine metabolism | -0.398 | -0,608; -0,189 | 0.033 |
| SCORE2 | PC.ae.C38.0 | Phosphatidylcholine metabolism | -0.132 | -0.195; -0.069 | 0.007 |

**Supplemental information 2** hsCRP as an additional covariable in the linear regression

Supplementary Table 10 shows the results for the linear regression additionally adjusted for hsCRP. Compared to the 23 significant associations in the main analysis, 19 associations were statistically significant. For stroke volume and early diastolic filling rate, the associations remained the same. End diastolic volume showed one less association and slightly smaller effect sizes. Peak ejection fraction was no longer significantly associated. Associations between the left ventricular wall thickness and metabolites remained the same in the SA after adjusting for hsCRP, indicating robust associations.

**Supplementary Table 15** Results of the full adjusted linear regression with hsCRP as additional covariable. Significance is defined as a p-value ≤ 0.05

| **MRI variable** | **Metabolite** | **Estimate** | **95% CI** | **P value** |
| --- | --- | --- | --- | --- |
| Stroke volume | C5 | -0.024 | -0.034; -0.013 | 0.001 |
| Stroke volume | PC.aa.C42.2 | 0.023 | 0.012; 0.033 | 0.005 |
| Stroke volume | PC.ae.C40.6 | 0.020 | 0.010; 0.030 | 0.020 |
| Stroke volume | PC.ae.C34.3 | 0.020 | 0.010; 0.031 | 0.020 |
| Stroke volume | PC.aa.C38.0 | 0.020 | 0.009; 0.030 | 0.035 |
| Early diastolic filling rate | PC.aa.C42.2 | 0.002 | 0.001; 0.003 | 0.011 |
| Early diastolic filling rate | C5 | -0.002 | -0.003; -0.001 | 0.010 |
| Early diastolic filling rate | lysoPC.a.C17.0 | 0.002 | 0.001; 0.002 | 0.049 |
| End diastolic volume | lysoPC.a.C17.0 | 0.012 | 0.006; 0.019 | 0.047 |
| All segments | Ala | -0.357 | -0.515; -0.199 | 0.002 |
| Apical segment | SDMA | -0.292 | -0.430; -0.154 | 0.006 |
| Apical segment | Ala | -0.279 | -0.411; -0.147 | 0.006 |
| Basal segment | Ala | -0.261 | -0.399; -0.123 | 0.036 |
| Inferior segment | SDMA | -0.292 | -0.447; -0.135 | 0.032 |
| Inferior segment | Creatinine | -0.262 | -0.392; -0.131 | 0.014 |
| Inferior segment | Ala | -0,336 | -0.482; -0.191 | 0.001 |
| Lateral segment | Ala | -0.313 | -0.454; -0.172 | 0.002 |
| Septal segment | Ala | -0,297 | -0.456; -0.138 | 0.041 |
| SCORE2 | PC.ae.C38.0 | -0,122 | -0.184; -0.060 | 0.019 |

References

1. Maulik U, Bandyopadhyay S. Performance evaluation of some clustering algorithms and validity indices. IEEE Transactions on Pattern Analysis and Machine Intelligence. 2002;24(12):1650-4.

2. Hartigan JA, Wong MA. Algorithm AS 136: A K-Means Clustering Algorithm. Journal of the Royal Statistical Society Series C (Applied Statistics). 1979;28(1):100-8.
